# Homologation of the Fischer Indolization: A Quinoline Synthesis via Homo‐Diaza‐Cope Rearrangement

**DOI:** 10.1002/anie.202005798

**Published:** 2020-09-07

**Authors:** Gabriela Guillermina Gerosa, Sebastian Armin Schwengers, Rajat Maji, Chandra Kanta De, Benjamin List

**Affiliations:** ^1^ Max-Planck-Institut für Kohlenforschung Kaiser-Wilhelm-Platz 1 45470 Mülheim an der Ruhr Germany

**Keywords:** annulation, cyclopropane, DFT calculations, Fischer indolization, quinolines, rearrangements

## Abstract

We disclose a new Brønsted acid promoted quinoline synthesis, proceeding via homo‐diaza‐Cope rearrangement of *N*‐aryl‐*N*′‐cyclopropyl hydrazines. Our strategy can be considered a homologation of Fischer's classical indole synthesis and delivers 6‐membered N‐heterocycles, including previously inaccessible pyridine derivatives. This approach can also be used as a pyridannulation methodology toward constructing polycyclic polyheteroaromatics. A computational analysis has been employed to probe plausible activation modes and to interrogate the role of the catalyst.

## Graphical abstract

A new pyridannulation methodology proceeds via homo‐diaza‐Cope rearrangement of *N*‐aryl‐*N*′‐cyclopropyl hydrazines. It can be considered a homologation of Fischer's classical indole synthesis and delivers 6‐membered N‐heterocycles. It complements existing approaches toward polycyclic quinoline derivatives for biological and material science applications.

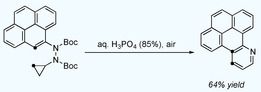

Among *N*‐heterocycles, quinoline derivatives occupy a place of particular relevance in chemical synthesis,^[1]^ medicinal chemistry,^[2]^ material science,^[3]^ and industry.^[4]^ Quinolines can generally be obtained by using several very reliable and powerful reactions of aniline derivatives, including those named after Skraup, Doebner‐von Miller, Friedländer, Pfitzinger, Conrad‐Limpach, and Combes.[^5^, ^6^] In recent years, our group has utilized the Fischer indole synthesis as a design platform for several new enantioselective processes, including a catalytic asymmetric Fischer indolization itself.^[7]^ Inspired by these investigations, we wondered if a homologous variant of this powerful transformation could be designed. The key steps of the Fischer indole synthesis involves a diaza‐Cope rearrangement of an ene‐hydrazine to an iminoethyl aniline, followed by cyclization and aromatization with loss of ammonia (Figure 1 A). We envisioned that homologation to a cyclopropyl hydrazine, should lead, upon rearrangement, to an iminopropyl aniline, which could cyclize and oxidatively aromatize to the corresponding quinoline derivative in an overall pyridannulation (Figure 1 B). Here we report on the successful realization of this conceptual design.

**Figure 1:**
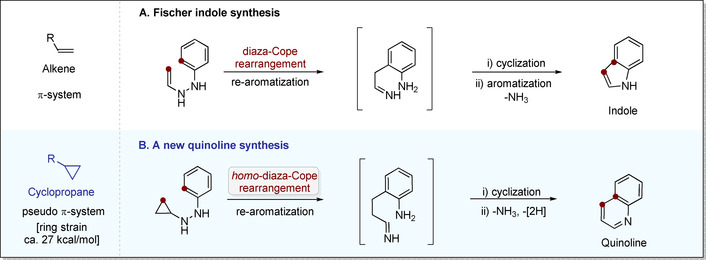
Outline of this study.

Considering the significant π‐character and pseudo‐sp^2^ hybridization, bonding and chemical properties of cyclopropane derivatives are often compared to those of the corresponding alkenes.^[8]^ This analogy has also been applied to pericyclic reactions, where double bonds have been replaced with cyclopropanes, leading to homologous versions of the parent pericyclic reaction.[^8b^, ^8c^] However, while nitrogen‐substituted cyclopropane derivatives display interesting reactivity, which manifests in ring strain driven ring‐opening reactions^[8d]^ and rearrangements,[^8e^, ^8f^, ^8g^] to the best of our knowledge, the homo‐sigmatropic rearrangement principle has not yet been applied to the 3,4‐diaza‐Cope rearrangement.

We initiated our studies by evaluating the reactivity of a toluene solution of bis‐Boc‐protected *N*‐aryl‐*N*′‐cyclopropylhydrazine **1 a** in the presence of a variety of acids (Table 1). Hydrochloric acid (in ether) at room temperature proved to be of insufficient reactivity to promote the desired reaction (entry 1). When we used aluminum chloride instead, full conversion of hydrazine **1 a** was observed but only decomposition of the starting material to several unidentified products was observed (entry 2). Interestingly, with triflic acid full consumption of the starting material was observed and 1,2,3,4‐tetrahydrobenzo[f]quinoline (**3 a**) was obtained in 10 % yield next to other unidentified compounds (entry 3). Remarkably, when the reaction was performed with *p*‐toluenesulfonic acid at elevated temperature, a promising mixture of products **2 a** and **3 a** was obtained (entry 4). Trifluoroacetic acid (entry 5) and phosphoric acid (85 %; entry 6) were also found to be suitable, albeit mixtures of product **2 a** and **3 a** were again obtained in moderate yields. H_3_PO_4_ (85 %) was found to be a superior acid in terms of combined chemical yields of **2 a** and **3 a** (68 %) and was selected for further optimizations.

**Table 1:** Reaction optimization^[a]^ 
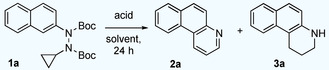

Entry	Acid	Solvent	*T*.(°C)	Yield **2 a**;**3 a** (**%**)^[b]^
1	HCl (ether)	PhMe	rt	–;–
2^[c]^	AlCl_3_	PhMe	rt	–;–
3^[c]^	TfOH	PhMe	rt	0;10
4	*p*‐TSA	PhMe	110	33;17
5	TFA	PhMe	110	42;18
6	H_3_PO_4_ (85 %)	PhMe	110	43;26
7^[d]^	H_3_PO_4_ (85 %)	1,2‐DCB	170	63;10
8^[d,e]^	H_3_PO_4_ (85 %)	1,2‐DCB	170	76;0

[a] Reactions were performed on 0.15 mmol scale under argon at 0.025 m concentration using 10 equiv of acid. [b] Yields were determined by ^1^H NMR spectroscopy using 1,3,5‐trimethoxybenzene as an internal standard. [c] Reactions were run for 2 h. [d] Reactions were performed under air. [e] Reaction was performed at 0.015 m concentration.

We reasoned that toward increasing the yield of the desired product **2 a**, an effective dehydrogenative aromatization of an anticipated dihydropyridine intermediate would be required. Indeed, when we heated the reaction mixture to 170 °C under air for 24 h in 1,2‐dichlobenzene, we could observe an increase in selectivity toward product **2 a** (entry 7). Gratifyingly, under these conditions, benzo[f]quinoline **2 a** was formed exclusively in good yield (76 %) when the reaction was diluted from 0.025 m to 0.015 m (entry 8).

With the optimized conditions in hand, we next explored the scope and generality of our new pyridannulation methodology (Scheme 1). Substrates **1** were readily obtained from the corresponding aryl bromides or aryl triflates.^[9]^ Our method was found to be well‐suited for aryl hydrazines with both electron‐donating and electron‐withdrawing groups. Irrespective of the nature and position of the substituent, both substrates **1 b** and **1 c** gave the desired products **2 b** and **2 c** in good yields. Similarly, the isomer of product **2 a**, benzo[h]quinoline **2 e** could also be obtained in acceptable yields. When polycyclic hydrazines were exposed to the optimized reaction conditions, the formation of higher quinolines with four rings (**2 d, 2 f**–**h**) and five rings (**2 i**) was observed in good yields. Even quinolines themselves can be obtained as illustrated with product **2 j**. Furthermore, symmetric, doubly‐annulated products **2 k** and **2 l** could be obtained as well, even though in these cases the yields were significantly reduced. Our approach enables access to heteropolycycles **2 b**, **2 c** and **2 i**, which were previously unknown.

**Scheme 1:**
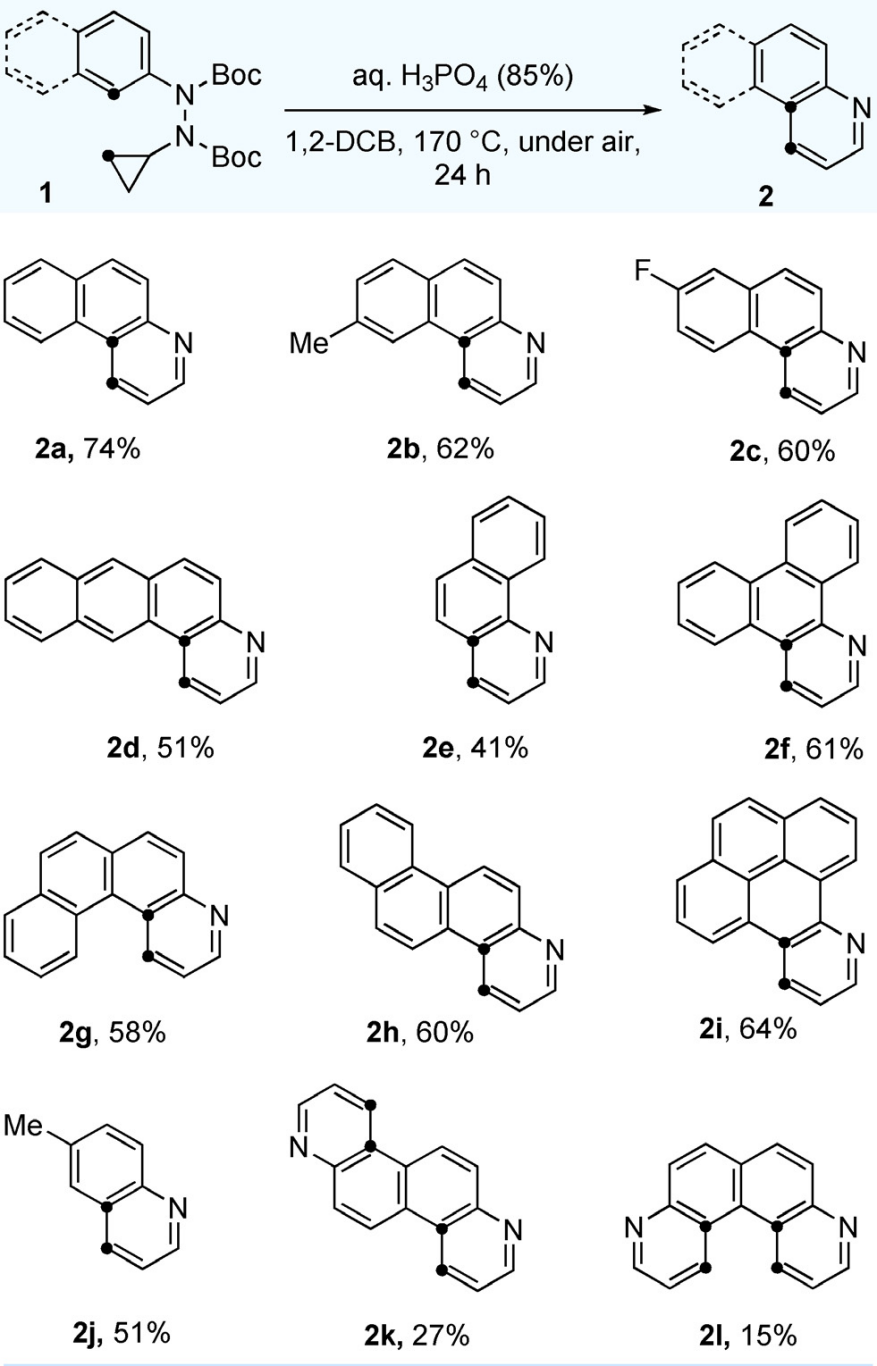
The reactions were carried out on a 0.3 mmol scale at a concentration of 0.015 M using 10 equivalents of acid.

The proposed mechanism of our new quinoline synthesis is illustrated in Figure 2. First, *N*‐Boc deprotection of **1 a** with acid occurs to generate doubly (or mono) protonated hydrazine salt **A**. This species then engages in the corresponding pseudo‐sigmatropic rearrangement to form iminium ion **B**. A tautomerizing rearomatization then furnishes intermediate **C**. Subsequently, cyclization to piperidine **D** proceeds, followed by loss of ammonia to furnish intermediate **E**. Depending on the reaction conditions, this intermediate can either undergo disproportionation (i) to form products **2 a** and **3 a**, or is oxidized (ii) by air to furnish product **2 a**.^[10]^ Furthermore, intermediate **E** can react in a Mannich type dimerization via tautomerization to the corresponding imine, followed by C−C‐bond formation and air oxidation to form product **4**. Indeed, when the reaction was performed in the presence of *p*‐TSA (Table 1, entry 4), a significant amount of dimer **4** was obtained (20 %), indirectly supporting the existence of intermediate **E**.

**Figure 2:**
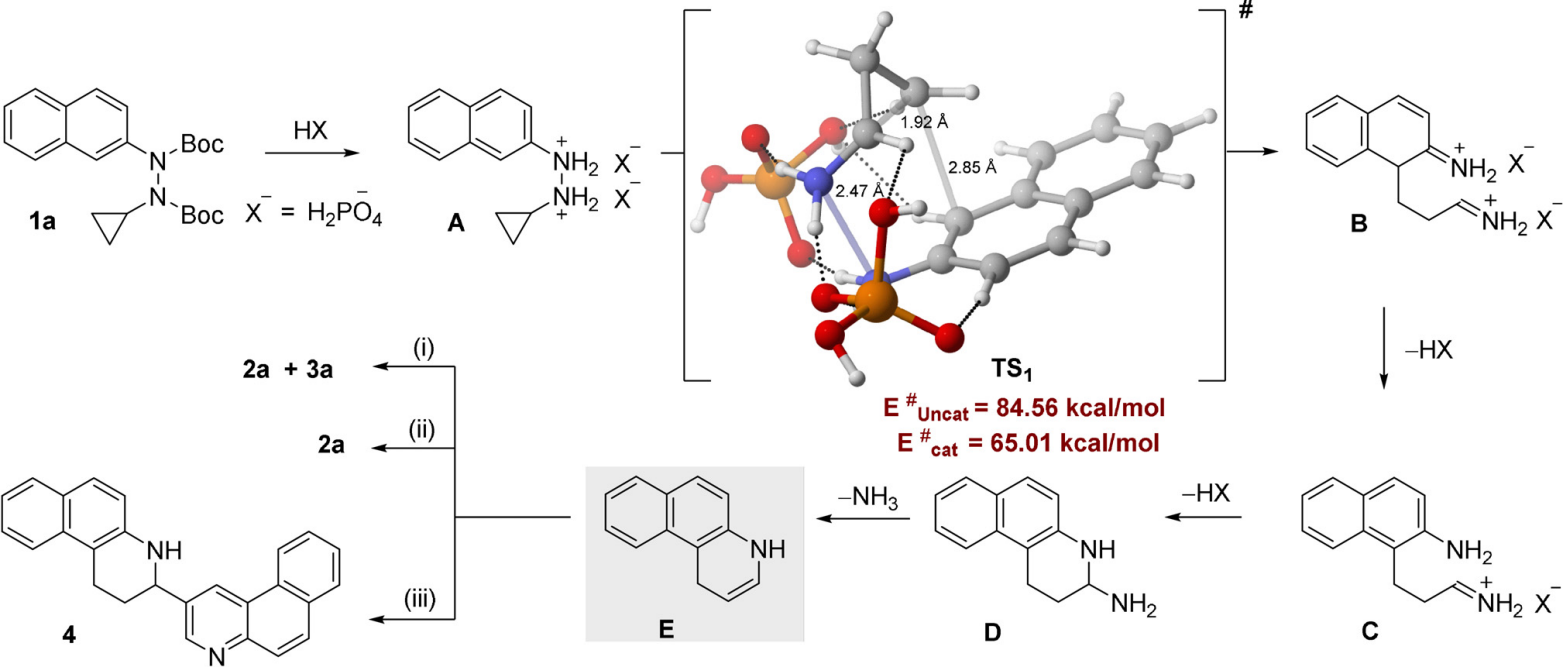
Proposed reaction pathway along with DFT computed activation energies (kcal mol^−1^) for the key transition states at the B3LYP‐D3/def2‐TZVP//PBE‐D3/def2‐SVP level of theory.

Towards a mechanistic understanding of our reaction, we employed DFT calculations using the ORCA suite (see SI).^[11]^ The activation barrier associated with the critical uncatalyzed pseudo‐sigmatropic C−C‐bond forming step is 84.56 kcal mol^−1^ at the B3LYP‐D3/def2‐TZVP//PBE‐D3/def2‐SVP level of theory.^[12]^ Notably, the high activation barrier is consistent with the breaking of a sigma bond and the dearomatization in the transition state (TS). Furthermore, the overall transformation is highly exothermic (−56.85 kcal mol^−1^) in nature, highlighting the importance of the rearomatization in driving the process. While computing the key transition states in the presence of the catalyst (H_3_PO_4_), we considered several modes of activation. Mono‐protonation of the hydrazine with a single molecule of H_3_PO_4_ within the TS leads to two distinct arrangements based on the site of the protonation (see SI). Alternatively, a highly organized di‐protonated transition state via the involvement of two catalyst molecules (TS_1_ in Figure 2) can be envisioned.[^7d^, ^13^] Indeed, di‐protonated TS_1_ is significantly more stable (>5 kcal mol^−1^) than the two mono‐protonated counterparts. Such an observation is intriguing given the entropic penalty associated with this TS. A closer inspection of the competing transition states revealed several additional non‐covalent interactions in TS_1_,^[14]^ which are absent in the alternative transition states (see SI), underscoring the importance of enthalpy in overriding the entropic bias. Furthermore, our effort to quantify CH‐O interaction using AIM^[15]^ indicates the difference is largely electrostatic in nature.^[16]^ Notably all these additional non‐covalent stabilizations also contribute significantly to the 20 kcal mol^−1^ reduction of the activation barrier as compared to the uncatalyzed reaction.

We have designed and developed a new approach to quinolines. Starting from the corresponding *N*‐aryl‐*N*′‐cyclopropylhydrazines and using phosphoric acid as mediator under aerobic conditions, various valuable heterocyclic products are obtained. We propose that our reaction takes place via a homo‐Fischer‐indolization‐type mechanism, featuring a pseudo‐pericyclic homo‐diaza‐Cope rearrangement as the key step. Contrary to the conventional mono‐protonated mechanism,[^7e^, ^17^] our computational analysis has identified a favorable di‐protonated pathway and offers a basis for the non‐linear effect observed in an analogous asymmetric benzidine rearrangement. Beyond the conceptual advancement, our pyridannulation methodology could complement existing approaches toward the synthesis of polycyclic quinoline derivatives for biological and material science applications, with the potential benefit of avoiding difficult to obtain and highly toxic aniline derivatives as intermediates.

## Conflict of interest

The authors declare no conflict of interest.

## Supporting information

As a service to our authors and readers, this journal provides supporting information supplied by the authors. Such materials are peer reviewed and may be re‐organized for online delivery, but are not copy‐edited or typeset. Technical support issues arising from supporting information (other than missing files) should be addressed to the authors.

Supplementary
