# Supplementary material for: Homologation of the Fischer Indolization: A Quinoline Synthesis via Homo‐Diaza‐Cope Rearrangement
Source: Angew Chem Int Ed Engl. 2020 Sep 7;59(46):20485–8. doi: 10.1002/anie.202005798 (PMC7693176; doi:10.1002/anie.202005798)
Supplement: Supplementary file 1 — Supplementary [file ANIE-59-20485-s001.pdf]

Angewandte Chemie
Eine Zeitschrift der Gesellschaft Deutscher Chemiker

Supporting Information

Homologation of the Fischer Indolization: A Quinoline Synthesis via Homo-Diaza-Cope Rearrangement

Gabriela Guillermina Gerosa, Sebastian Armin Schwengers, Rajat Maji, Chandra Kanta De, and Benjamin List**

anie_202005798_sm_miscellaneous_information.pdf

Supporting Information

Content

1. General Information and Instrumentation……………………………………………………………………………………..2

2. Synthesis and characterization of di-tert-butyl 1-cyclopropylhydrazine-1,2-dicarboxylate (**5**)…………………………………..3

2.1. Procedure……..3

2.2. Analytical data……...3

3. Synthesis and characterization of starting materials (**1**)………………………………………………………………………..3

3.1. General procedure A: cross-coupling reaction………………………………………………………………………….3

3.2. General procedure B: double cross-coupling reaction……………………………………………………………………4

3.3. Analytical data ……………………………………………………………………………………………………….....4

4. Synthesis and characterization of quinoline derivatives (**2**)……………………………………………………………………..8

4.1. General procedure……………………………………………………………………………………………............8

4.2. Analytical data ……………………………………………………………………….…………………………………8

5. Intermediate……....12

5.1. Procedure……....12

5.2. Analytical data……………………………………………………………………………….…………………………12

6. NMR spectra……………………………………………………………………………………………………….……....13

7. Computational Studies…………………………………………………………………………………………………….40

7.1. Method……….40

7.2. Discussion……...40

8. References…….……….51

1. General Information and Instrumentation

Unless otherwise stated, all reagents were purchased from commercial suppliers and used without further purification. All solvents used in the reactions were distilled from appropriate drying agents prior to use. Reactions were monitored by thin layer chromatography (TLC) on silica gel pre-coated plastic sheets (0.2 mm, Macherey-Nagel). Visualization was accomplished by irradiation with UV light at 254 nm and/or p-anisaldehyde stain. Preparative thin-layer chromatography (Prep.TLC) was performed on silica gel pre-coated glass plates SIL G-25 UV254 and SIL G-100 UV254 with 0.25 mm and 1.0 mm SiO_2 layers

(Macherey-Nagel Column chromatography was performed on Merck silica gel (60, particle size 0.040–0.063 mm). ^{1}H and ^{13}C NMR spectra were recorded on a Bruker AV-500 AV-400 or DPX-300 spectrometer in deuterated solvents. Proton chemical shifts are reported in ppm (δ) relative to the solvent resonance employed as the internal standard ($CDCl_3$ δ 7.26 ppm). Data are reported as follows: chemical shift, multiplicity (s = singlet, d = doublet, t = triplet, q = quartet, p = pentet, s = sextet, h = heptet, m = multiplet, br = broad), coupling constants (Hz) and integration. ^{13}C chemical shifts are reported in ppm with the solvent resonance as the internal standard. High-resolution mass spectra were determined on a Bruker APEX III FTMS (7 T magnet). Transition state structures were verified by the presence of a single imaginary vibrational frequency.

2. Synthesis and characterization of di-tert-butyl 1-cyclopropylhydrazine-1,2-dicarboxylate (5)

2.1. Procedure[1]

To a solution of cyclopropylmagnesium bromide (0.7 M in THF, 14.9 mL) in THF (8 mL) was added di-tert-butyl azadicarboxylate (2 g, 8,7 mmol) at -78° C under argon. The mixture was stirred at -78° C for 1 hour. The reaction mixture was quenched by addition of saturated NH_4Cl (10 mL) at 0° C and then diluted with H_2O (50 mL) and extracted with EtOAc (2x50 mL). The combined organic phase was dried with Na_2SO_4, filtered, and concentrated under reduced pressure. The residue was purified by column chromatography on silica gel (hexanes/EtOAc = 9:1; *v/v*) to give di-tert-butyl 1-cyclopropylhydrazine-1,2-dicarboxylate (**5**) in 72% yield as white solid.

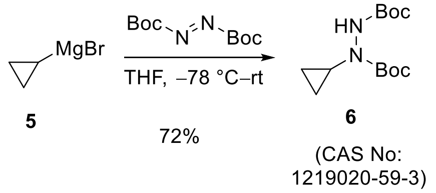

2.2. Analytical data

Signal broadening and additional splitting could be observed due to the presence of rotamers.

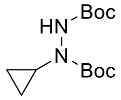

^{1}H NMR (501 MHz, Chloroform-*d*) δ 6.51–5.99 (m, 1H), 3.03–2.76 (m, 1H), 1.50–1.39 (m, 18H), 0.77–0.62 (m, 4H).

^{13}C NMR (126 MHz, Chloroform-*d*) δ 156.18, 155.66, 81.28, 81.16, 31.97, 28.33, 7.43.

HRMS (ESI) *m/z* calculated for $C_{13}H_{24}N_2O_4Na_1$ $[M+Na]^+$: 295.162826; found: 295.163150.

3. Synthesis and characterization of starting materials (1)

3.1. General procedure A: cross-coupling reaction

A flame-dried Schlenk flask was charged with a magnetic stir-bar, cyclopropylhydrazine **6** (1.0 equiv.), aryl bromide or aryl triflate **7** (1.2 equiv.), Cs_2CO_3 (1.4 equiv.), and $Pd(OAc)_2$ (0.15 equiv.) under argon. Then toluene (0.5 M) was added to the schlenk flask. After degassing, P(*t*-Bu)$_3$ (0.5 equiv.) was added to the mixture, which was stirred at 100 °C. After 24 h, the reaction mixture was cooled to room temperature and H_2O was added. The mixture was extracted with EtOAc (3x20 mL). The organic phase was dried with Na_2SO_4, filtered, and concentrated under reduced pressure. The residue was purified by column chromatography on silica gel (hexanes/EtOAc = 9:1; *v/v*).

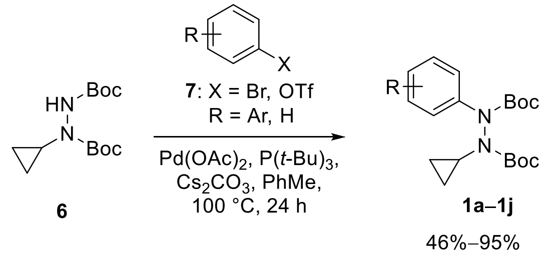

3.2. General procedure B: double cross-coupling reaction

A flame-dried Schlenk flask was charged with a magnetic stir-bar, cyclopropylhydrazine **5** (3.0 equiv.), aryl dibromide **7** (1.0 equiv.), Cs_2CO_3 (3.0 equiv.), and $Pd(OAc)_2$ (0.15 equiv.) under argon. Then toluene (0.5 M) was added to the schlenk flask. After degassing, P(*t*-Bu)$_3$ (0.5 equiv.) was added to the mixture, which was stirred at 100 °C. After 24 h, the reaction mixture was cooled to room temperature and H_2O was added. The mixture was extracted with EtOAc (3x20 mL). The organic phase was dried with Na_2SO_4, filtered, and concentrated under reduced pressure. The residue was purified by column chromatography on silica gel (hexanes/EtOAc = 8.5:1.5; *v/v*).

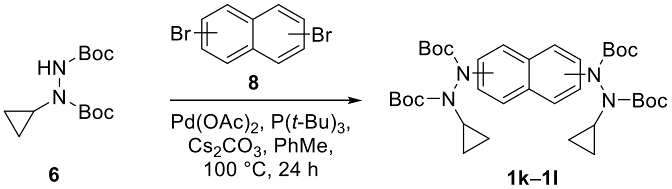

6 **1k–1l**

15%–27%

3.3. Analytical data:

di-*tert*-butyl 1-cyclopropyl-2-(naphthalen-2-yl)hydrazine-1,2-dicarboxylate (1a): Following the general procedure A, 93% yield as yellow oil. *Signal broadening and additional splitting could be observed due to the presence of rotamers.*

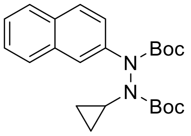

1H NMR (501 MHz, Chloroform-d) δ 7.86–7.72 (m, 4H), 7.53 (s, 1H), 7.49–7.38 (m, 2H), 3.08–2.92 (m, 1H), 1.60–1.46 (m, 18H), 0.87–0.73 (m, 2H), 0.72–0.59 (m, 2H).

^{13}C NMR (126 MHz, Chloroform-*d*) δ 153.20, 133.55, 131.22, 128.24, 128.08, 127.95, 127.57, 126.38, 126.15, 125.59, 82.16, 81.71, 81.51, 60.52, 32.50, 32.27, 31.91, 29.84, 28.40, 14.34, 8.27, 7.18, 6.29.

HRMS (ESI) *m/z* calculated for $C_{23}H_{30}N_2O_4Na_1$ $[M+Na]^+$: 421.209776; found: 421.209440.

di-*tert*-butyl 1-cyclopropyl-2-(7-methylnaphthalen-2-yl)hydrazine-1,2-dicarboxylate (1b): Following the general procedure A, 76% yield as yellow oil. *Signal broadening and additional splitting could be observed due to the presence of rotamers.*

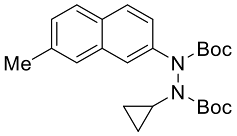

^{1}H NMR (501 MHz, Chloroform-*d*) δ 7.70–7.54 (m, 3H), 7.51–7.28 (m, 2H), 7.23–7.11 (m, 1H), 3.00–2.81 (m, 1H), 2.41 (s, 3H), 1.58–1.27 (m, 18H), 0.84–0.64 (m, 2H), 0.64–0.48 (m, 2H).

^{13}C NMR (126 MHz, Chloroform-*d*) δ 153.22, 136.05, 135.77, 133.77, 129.50, 127.90, 127.38, 127.04, 126.92, 82.04, 81.63, 81.42, 32.46, 31.88, 28.49, 28.41, 28.40, 21.82, 8.23, 7.15, 6.29.

HRMS (ESI) *m/z* calculated for $C_{24}H_{32}N_2O_4Na_1$ $[M+Na]^+$: 435.225426; found: 435.225310.

di-*tert*-butyl 1-cyclopropyl-2-(6-fluoronaphthalen-2-yl)hydrazine-1,2-dicarboxylate (1c): Following the general procedure A, 82% yield as yellow oil. *Signal broadening and additional splitting could be observed due to the presence of rotamers.*

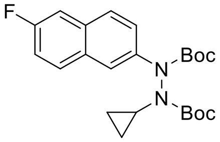

^{1}H NMR (501 MHz, Chloroform-*d*) δ 7.89–7.65 (m, 3H), 7.54 (s, 1H), 7.45–7.36 (m, 1H), 7.26–7.18 (m, 1H), 3.10–2.89 (m, 1H), 1.75–1.39 (m, 18H), 0.87–0.72 (m, 2H), 0.72–0.59 (m, 2H).

^{13}C NMR (126 MHz, Chloroform-*d*) δ 161.56, 159.61, 153.18, 131.84, 130.48, 130.27, 130.20, 127.56, 123.44, 122.64, 120.56, 119.63, 116.90, 116.45, 110.79, 110.63, 82.25, 81.76, 81.58, 32.50, 31.91, 28.47, 28.41, 28.38, 8.27, 7.17, 6.97, 6.31.

HRMS (ESI) *m/z* calculated for $C_{23}H_{29}F_1N_2O_4Na_1$ $[M+Na]^+$: 439.200355; found: 439.200280.

di-*tert*-butyl 1-(anthracen-2-yl)-2-cyclopropylhydrazine-1,2-dicarboxylate (1d): Following the general procedure A, 71% yield as yellow oil. *Signal broadening and additional splitting could be observed due to the presence of rotamers.*

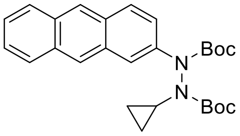

^{1}H NMR (501 MHz, Chloroform-*d*) δ 8.41–8.31 (m, 2H), 8.03–7.86 (m, 4H), 7.57 (s, 1H), 7.50–7.38 (m, 2H), 3.12–2.96 (m, 1H), 1.67–1.47 (m, 18H), 0.93–0.76 (m, 2H), 0.76–0.61 (m, 2H).

^{13}C NMR (126 MHz, Chloroform-*d*) δ 156.04, 153.19, 138.99, 132.12, 132.02, 131.62, 129.91, 129.70, 122.68, 82.24, 81.75, 81.56, 32.54, 32.24, 31.95, 28.50, 28.44, 28.40, 8.33, 7.23, 6.96, 6.32.

HRMS (ESI) *m/z* calculated for $C_{27}H_{32}N_2O_4Na_1$ $[M+Na]^+$: 471.225427; found: 471.225510.

di-*tert*-butyl 1-cyclopropyl-2-(naphthalen-1-yl)hydrazine-1,2-dicarboxylate (1e): Following the general procedure A, 52% yield as yellow oil. *Signal broadening and additional splitting could be observed due to the presence of rotamers.*

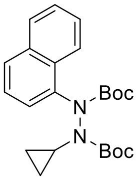

^{1}H NMR (501 MHz, Chloroform-*d*) δ 8.03 (s, 1H), 7.89–7.60 (m, 3H), 7.58–7.36 (m, 3H), 3.13–2.81 (m, 1H), 1.66–1.49 (m, 14H), 1.25 (s, 4H), 0.94–0.22 (m, 4H).

^{13}C NMR (126 MHz, Chloroform-*d*) δ 134.27, 133.97, 130.56, 130.42, 128.25, 128.10, 127.74, 126.38, 125.83, 124.00, 123.88, 122.42, 81.75, 81.54, 32.46, 31.90, 28.57, 28.49, 28.44, 28.03, 7.95, 6.29.

HRMS (ESI) *m/z* calculated for $C_{23}H_{30}N_2O_4Na_1$ $[M+Na]^+$: 421.209776; found: 421.209560.

di-*tert*-butyl 1-cyclopropyl-2-(phenanthren-9-yl)hydrazine-1,2-dicarboxylate (1f): Following the general procedure A, 53% yield as yellow oil. *Signal broadening and additional splitting could be observed due to the presence of rotamers.*

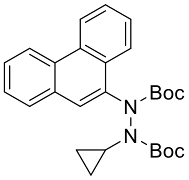

^{1}H NMR (501 MHz, Chloroform-*d*) δ 8.74–8.61 (m, 2H), 8.22–7.95 (m, 2H), 7.92–7.76 (m, 1H), 7.72–7.52 (m, 4H), 3.20–2.84 (m, 1H), 1.80–1.48 (m, 13H), 1.24 (s, 5H), 0.99–0.25 (m, 4H).

^{13}C NMR (126 MHz, Chloroform-*d*) δ 131.90, 130.81, 129.94, 129.05, 126.26, 124.72, 122.92, 122.81, 81.77, 32.51, 32.07, 28.56, 28.47, 28.05, 9.21, 8.31, 7.85, 6.42.

HRMS (ESI) *m/z* calculated for $C_{27}H_{32}N_2O_4Na_1$ $[M+Na]^+$: 471.225426 ; found: 471.225400.

di-*tert*-butyl 1-cyclopropyl-2-(phenanthren-3-yl)hydrazine-1,2-dicarboxylate (1g): Following the general procedure A, 66% yield as yellow oil. *Signal broadening and additional splitting could be observed due to the presence of rotamers.*

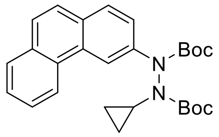

^{1}H NMR (501 MHz, Chloroform-*d*) δ 8.66–8.58 (m, 2H), 7.97–7.82 (m, 2H), 7.76–7.61 (m, 4H), 7.60–7.53 (m, 1H), 3.12–2.95 (m, 1H), 1.63–1.48 (m, 18H), 0.93–0.75 (m, 2H), 0.75–0.60 (m, 2H).

^{13}C NMR (126 MHz, Chloroform-*d*) δ 155.74, 153.15, 132.40, 131.91, 130.16, 128.69, 127.54, 127.29, 127.14, 126.96, 126.85, 126.48, 82.25, 81.73, 81.57, 32.54, 31.95, 28.50, 28.41, 8.34, 7.25, 6.88, 6.21.

HRMS (ESI) *m/z* calculated for $C_{27}H_{32}N_2O_4Na_1$ $[M+Na]^+$: 471.225426; found: 471.225440.

di-*tert*-butyl 1-cyclopropyl-2-(phenanthren-2-yl)hydrazine-1,2-dicarboxylate (1h): Following the general procedure A, 95% yield as yellow oil. *Signal broadening and additional splitting could be observed due to the presence of rotamers.*

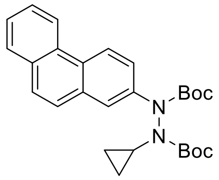

^{1}H NMR (501 MHz, Chloroform-*d*) δ 8.79–8.63 (m, 1H), 8.63–8.55 (m, 1H), 7.92–7.86 (m, 1H), 7.86–7.80 (m, 1H), 7.73–7.68 (m, 2H), 7.68–7.56 (m, 3H), 3.17–2.97 (m, 1H), 1.64–1.52 (m, 18H), 0.95–0.76 (m, 2H), 0.75–0.60 (m, 2H).

^{13}C NMR (126 MHz, Chloroform-*d*) δ 153.22, 140.54, 132.33, 130.59, 129.53, 128.77, 126.84, 126.65, 126.43, 122.64, 82.22, 81.73, 81.56, 32.54, 31.99, 28.46, 28.44, 8.36, 7.35, 7.01, 6.22.

HRMS (ESI) *m/z* calculated for $C_{27}H_{32}N_2O_4Na_1$ $[M+Na]^+$: 471.225427; found: 471.225460.

di-*tert*-butyl 1-cyclopropyl-2-(pyren-4-yl)hydrazine-1,2-dicarboxylate (1i): Following the general procedure A, 46% yield as yellow oil. *Signal broadening and additional splitting could be observed due to the presence of rotamers.*

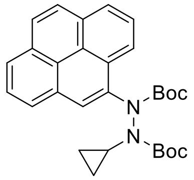

^{1}H NMR (501 MHz, Chloroform-*d*) δ 8.52–8.29 (m, 2H), 8.25–7.94 (m, 7H), 3.25–2.93 (m, 1H), 1.76–1.55 (m, 13H), 1.32–1.18 (m, 5H), 1.02–0.22 (m, 4H).

^{13}C NMR (126 MHz, Chloroform-*d*) δ 131.44, 131.31, 131.12, 127.81, 127.26, 127.17, 126.19, 126.04, 125.63, 125.47, 124.20, 123.32, 81.87, 70.72, 68.09, 32.67, 32.20, 32.06, 29.84, 28.60, 28.49, 28.06, 25.74, 9.19, 8.29, 6.51.

HRMS (ESI) *m/z* calculated for $C_{29}H_{32}N_2O_4Na_1$ $[M+Na]^+$: 495.225427; found: 495.225450.

di-tert-butyl 1-cyclopropyl-2-(p-tolyl)hydrazine-1,2-dicarboxylate (1j): Following the general procedure A, 80% yield as yellow oil. *Signal broadening and additional splitting could be observed due to the presence of rotamers.*

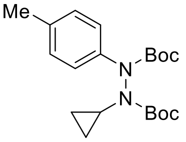

^{1}H NMR (501 MHz, Chloroform-d) δ 7.23–7.10 (m, 2H), 7.07–6.97 (m, 2H), 2.85 (m, 1H), 2.24 (s, 3H), 1.61–1.24 (m, 18H), 0.77–0.48 (m, 4H).

^{13}C NMR (126 MHz, Chloroform-*d*) δ 153.09, 134.92, 129.02, 123.05, 122.45, 81.54, 81.29, 81.07, 32.14, 31.55, 29.66, 28.19, 20.83, 7.89, 6.76, 6.38.

HRMS (ESI) *m/z* calculated for $C_{20}H_{30}N_2O_4Na_1$ $[M+Na]^+$: 385.209776; found: 385.209680.

tetra-tert-butyl 2,2'-(naphthalene-2,6-diyl)bis(1-cyclopropylhydrazine-1,2-dicarboxylate) (1k): Following the general procedure B, 77% yield as yellow oil. *Signal broadening and additional splitting could be observed due to the presence of rotamers.*

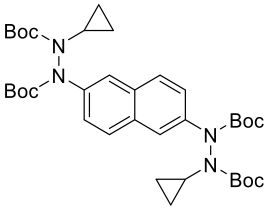

^{1}H NMR (501 MHz, Chloroform-d) δ 7.83–7.63 (m, 4H), 7.50 (s, 2H), 3.11–2.80 (m, 2H), 1.62–1.43 (m, 36H), 0.85–0.58 (m, 8H).

^{13}C NMR (126 MHz, Chloroform-*d*) δ 153.04, 131.03, 128.03, 82.03, 81.57, 81.38, 32.35, 31.75, 29.70, 28.35, 28.26, 14.12, 8.12, 7.04, 6.14.

HRMS (API) *m/z* calculated for $C_{36}H_{52}N_4O_8Na_1$ $[M+Na]^+$: 691.367734; found: 691.367500.

tetra-tert-butyl 2,2'-(naphthalene-2,7-diyl)bis(1-cyclopropylhydrazine-1,2-dicarboxylate) (1l): Following the general procedure B, 72% yield as yellow oil. *Signal broadening and additional splitting could be observed due to the presence of rotamers.*

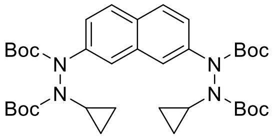

^{1}H NMR (501 MHz, Chloroform-d) δ 7.83–7.59 (m, 4H), 7.58–7.34 (m, 2H), 3.06–2.88 (m, 2H), 1.69–1.38 (m, 36H), 0.89–0.71 (m, 4H), 0.70 – 0.54 (m, 4H).

^{13}C NMR (126 MHz, Chloroform-*d*) δ 153.03, 133.55, 127.68, 82.03, 81.54, 32.35, 31.79, 28.28, 8.13, 7.03, 6.15.

HRMS (ESI) *m/z* calculated for $C_{36}H_{52}N_4O_8Na_1$ $[M+Na]^+$: 691.367734; found: 691.367430.

4. Synthesis and characterization of quinoline derivatives (2)

4.1. General procedure:

A round bottom flask, equipped with a magnetic stir-bar and reflux condenser was charged with stating material **1** (1.0 equiv.). 1,2-DCB (0.015 M) and $H_3PO_{4(aq)}$ (85%) was added and the reaction mixture refluxed for 24 h at 170 °C under an air filled balloon (1 atm). After cooling to room temperature, the organic phase was carefully decanted and the residue treated with NaOH (1 N) and DCM. After stirring for 10 min the mixture was transferred to a separation funnel and extracted with DCM (5x7 ml). The combined organic phase was dried over Na_2SO_4, concentrated to dryness followed by purification by flash column chromatography on silica gel.

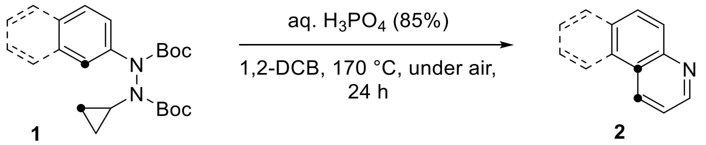

1 **2**

4.2. Analytical data:

benzo[f]quinoline (2a): Prepared according to the general procedure (DCM/EtOAc = 4:1; *v/v*), 74% yield as white solid.

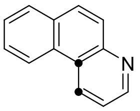

^{1}H NMR (501 MHz, Chloroform-*d*) δ 8.98–8.95 (m, 1H), 8.94 (d, *J* = 8.4, 1H), 8.61 (d, *J* = 8.1 Hz, 1H), 8.02–7.96 (m, 2H), 7.94 (d, *J* = 7.6 Hz, 1H), 7.67 (dt, *J* = 21.8, 7.2 Hz, 2H), 7.55 (dd, *J* = 8.3, 4.3 Hz, 1H).

^{13}C NMR (126 MHz, Chloroform-*d*) δ 149.87, 148.37, 131.82, 130.99, 130.81, 129.78, 128.84, 128.35, 127.45, 127.23, 125.56, 122.72, 121.44.

HRMS (ESI) *m/z* calculated for $C_{13}H_{10}N_1$ $[M+H]^+$: 180.080774; found: 180.080750.

9-methylbenzo[f]quinoline (2b): Prepared according to the general procedure (DCM/EtOAc = 4:1; *v/v*), 62% yield as pale yellow solid.

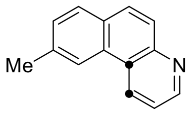

^{1}H NMR (501 MHz, Chloroform-*d*) δ 9.01 (dd, *J* = 8.3, 1.5 Hz, 1H), 8.95 (dd, *J* = 4.5, 1.6 Hz, 1H), 8.41 (s, 1H), 8.00 (dd, *J* = 9.1 Hz, 2H), 7.85 (d, *J* = 8.1 Hz, 1H), 7.59 (dd, *J* = 8.3, 4.4 Hz, 1H), 7.51 (dd, *J* = 8.0, 1.6 Hz, 1H), 2.64 (s, 3H).

^{13}C NMR (126 MHz, Chloroform-*d*) δ 148.55, 147.48, 137.54, 131.86, 131.63, 129.81, 129.59, 129.55, 128.82, 126.19, 125.56, 122.49, 121.23, 22.25.

HRMS (GC-EI) *m/z* calculated for $C_{14}H_{11}N_1$ [M]: 193.088599; found: 193.088650.

8-fluorobenzo[f]quinoline (2c): Prepared according to the general procedure (DCM/EtOAc = 4:1; *v/v*), 60% yield as pale yellow solid.

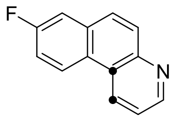

^{1}H NMR (501 MHz, Chloroform-*d*) δ 8.95 (dd, *J* = 4.4, 1.6 Hz, 1H), 8.88 (dd, *J* = 8.2, 1.5 Hz, 1H), 8.59 (dd, *J* = 9.1, 5.2 Hz, 1H), 8.04 (d, *J* = 9.1 Hz, 1H), 7.91 (d, *J* = 9.1 Hz, 1H), 7.60–7.55 (m, 2H), 7.44 (ddd, *J* = 9.0, 8.2, 2.7 Hz, 1H).

^{19}F NMR (471 MHz, Chloroform-*d*) δ –113.52 (dd, *J* = 7.9, 7.5 Hz), –113.52 (dd, *J* = 15.3, 9.1 Hz).

^{13}C NMR (126 MHz, Chloroform-*d*) δ 162.84, 160.87, 149.54, 147.66, 133.32, 133.25, 130.81, 130.28, 130.25, 129.45, 126.31, 126.30, 125.47, 125.11, 125.04, 121.81, 116.43, 116.24, 113.12, 112.96.

HRMS (GC-EI) *m/z* calculated for $C_{13}H_8N_1F_1$ [M]: 197.063527; found: 197.063550.

naphtho[2,3-f]quinoline (2d): Prepared according to the general procedure (DCM/EtOAc = 4:1; *v/v*), 51% yield as pale yellow solid.

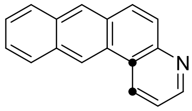

^{1}H NMR (501 MHz, Chloroform-*d*) δ 9.01–8.95 (m, 2H), 8.91 (m, 1H), 8.33 (s, 1H), 8.11–8.05 (m, 1H), 8.03–8.00 (m, 1H), 7.97 (d, *J* = 9.3 Hz, 1H), 7.84 (d, *J* = 9.2 Hz, 1H), 7.60–7.54 (m, 2H), 7.52 (dd, *J* = 8.2, 4.4 Hz, 1H).

^{13}C NMR (126 MHz, Chloroform-*d*) δ 149.38, 148.77, 132.26, 131.96, 131.55, 130.70, 129.87, 128.34, 128.16, 127.96, 127.78, 127.24, 126.32, 126.27, 125.95, 121.80, 121.46.

HRMS (GC-EI) *m/z* calculated for $C_{17}H_{11}N_1$ [M]: 229.088599; found: 229.088790.

benzo[h]quinoline (2e): Prepared according to the general procedure (hexane/EtOAc = 4:1; *v/v*), 41% yield as pale yellow solid.

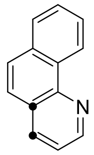

^{1}H NMR (501 MHz, Chloroform-*d*) δ 9.31 (d, *J* = 7.9 Hz, 1H), 9.01 (dd, *J* = 4.4, 1.8 Hz, 1H), 8.18 (dd, *J* = 8.0, 1.8 Hz, 1H), 7.98–7.87 (m, 1H), 7.82 (d, *J* = 8.8 Hz, 1H), 7.77–7.70 (m, 2H), 7.69 (d, *J* = 8.8 Hz, 1H), 7.53 (dd, *J* = 8.0, 4.4 Hz, 1H).

^{13}C NMR (126 MHz, Chloroform-*d*) δ 148.91, 146.67, 136.03, 133.76, 131.59, 128.37, 127.96, 127.94, 127.24, 126.57, 125.47, 124.52, 121.93.

HRMS (GC-EI) *m/z* calculated for $C_{13}H_9N_1$ [M]: 179.072949; found: 179.072940.

dibenzo[f,h]quinoline (2f): Prepared according to the general procedure (hexane/EtOAc = 4:1; *v/v*), 61% yield as pale yellow solid.

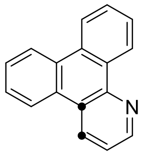

^{1}H NMR (501 MHz, Chloroform-*d*) δ 9.37–9.31 (m, 1H), 8.98 (dd, *J* = 4.3, 1.6 Hz, 1H), 8.87 (dd, *J* = 8.3, 1.6 Hz, 1H), 8.69–8.66 (m, 1H), 8.65–8.61 (m, 1H), 8.59–8.55 (m, 1H), 7.79–7.72 (m, 2H), 7.72–7.65 (m, 2H), 7.58 (dd, *J* = 8.2, 4.4 Hz, 1H).

^{13}C NMR (126 MHz, Chloroform-*d*) δ 148.95, 146.60, 131.45, 131.01, 130.95, 129.96, 129.03, 128.83, 128.03, 127.67, 127.50, 125.52, 124.65, 123.58, 123.39, 122.71, 122.28.

HRMS (GC-EI) *m/z* calculated for $C_{17}H_{11}N_1$ [M]: 229.088599; found: 229.088810.

naphtho[1,2-f]quinoline (2g): Prepared according to the general procedure (DCM/EtOAc = 4:1; *v/v*), 58% yield as off-white solid. The desired product was isolated as a mixture of regioisomers (ratio: 9:1).

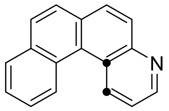

^{1}H NMR (501 MHz, Chloroform-*d*) δ 9.37 (d, *J* = 8.3 Hz, 1H), 9.00 (dd, *J* = 4.2, 1.6 Hz, 1H), 8.91 (d, *J* = 8.3 Hz, 1H), 8.17 (d, *J* = 8.7 Hz, 1H), 8.08–8.02 (m, 2H), 7.94 (d, *J* = 8.6 Hz, 1H), 7.85 (d, *J* = 8.8 Hz, 1H), 7.70 (td, *J* = 7.7, 1.6 Hz, 1H), 7.66 (td, *J* = 7.7, 1.7 Hz 1H), 7.59–7.54 (m, 1H).

^{13}C NMR (126 MHz, Chloroform-*d*) δ 151.10, 149.02, 148.85, 136.11, 135.79, 133.63, 130.97, 130.87, 130.14, 128.98, 128.70, 128.41, 127.45, 126.78, 126.42, 125.57, 120.49.

HRMS (GC-EI) *m/z* calculated for $C_{17}H_{11}N_1$ [M]: 229.088599; found: 229.088800.

naphtho[2,1-f]quinoline (2h): Prepared according to the general procedure (DCM/EtOAc = 4:1; *v/v*), 60% yield as off-white solid.

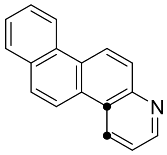

^{1}H NMR (501 MHz, Chloroform-*d*) δ 9.00–8.98 (m, 2H), 8.90 (d, *J* = 9.3 Hz, 1H), 8.73 (d, *J* = 8.3 Hz, 1H), 8.52 (d, *J* = 8.9 Hz, 1H), 8.26 (d, *J* = 9.3 Hz, 1H), 7.98 (dd, *J* = 8.7 Hz, 2H), 7.71 (ddd, *J* = 8.3, 6.8, 1.4 Hz, 1H), 7.68–7.63 (m, 1H), 7.59–7.55 (m, 1H).

^{13}C NMR (126 MHz, Chloroform-*d*) δ 149.42, 147.34, 132.43, 131.88, 130.31, 128.75, 128.33, 128.25, 127.92, 127.80, 127.24, 127.03, 125.68, 125.52, 123.25, 121.46, 120.51.

HRMS (GC-EI) *m/z* calculated for $C_{17}H_{11}N_1$ [M]: 229.088599; found: 229.088970.

phenanthro[4,5-fgh]quinoline (2i): Prepared according to the general procedure (hexane/EtOAc = 4:1; *v/v*), 64% yield as pale yellow solid.

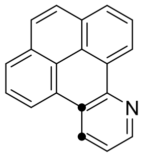

^{1}H NMR (501 MHz, Chloroform-*d*) δ 9.47 (dd, *J* = 7.7, 1.2 Hz, 1H), 9.00 (dd, *J* = 4.3, 1.6 Hz, 1H), 8.89 (dd, *J* = 8.2, 1.6 Hz, 1H), 8.63 (d, *J* = 7.7 Hz, 1H), 8.20 (d, *J* = 7.7 Hz, 1H), 8.10 (dd, *J* = 7.8, 1.0 Hz, 1H), 8.07 (dd, *J* = 7.7 Hz, 1H), 7.99 (d, *J* = 8.9 Hz, 1H), 7.94 (d, *J* = 8.8, 6.4 Hz, 1H), 7.92 (dd, *J* = 7.7 Hz, 1H), 7.56 (dd, *J* = 8.2, 4.4 Hz, 1H).

^{13}C NMR (126 MHz, Chloroform-*d*) δ 149.15, 147.05, 131.65, 131.33, 131.19, 129.98, 128.19, 127.67, 127.59, 127.12, 126.77, 126.58, 126.17, 125.69, 125.18, 124.04, 122.83, 122.44, 120.32.

HRMS (GC-EI) *m/z* calculated for $C_{19}H_{11}N_1$ [M]: 253.088599; found: 253.088730.

6-methylquinoline (2j): Prepared according to the general procedure (hexane/EtOAc = 4:1; *v/v*), 51% yield as pale yellow solid.

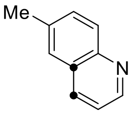

^{1}H NMR (501 MHz, Chloroform-*d*) δ 8.85 (dd, *J* = 4.2, 1.8 Hz, 1H), 8.09–8.05 (m, 1H), 8.00 (d, *J* = 8.5 Hz, 1H), 7.59–7.57 (m, 1H), 7.55 (dd, *J* = 8.6, 2.0 Hz, 1H), 7.36 (dd, *J* = 8.2, 4.2 Hz, 1H), 2.54 (s, 3H).

^{13}C NMR (126 MHz, Chloroform-d) δ 149.51, 146.88, 136.40, 135.39, 131.76, 129.09, 128.33, 126.58, 121.07, 21.59.

HRMS (ESI) *m/z* calculated for $C_{10}H_{10}N_1$ $[M+H]^+$: 144.080774; found: 144.080940.

quinolino[6,5-f]quinoline (2k): Prepared according to the general procedure (EtOAc), 27% yield as pale yellow solid.

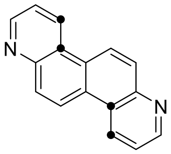

^{1}H NMR (501 MHz, Chloroform-*d*) δ 9.11–9.00 (m, 4H), 8.86 (d, *J* = 9.2 Hz, 2H), 8.31 (d, *J* = 9.2 Hz, 2H), 7.66 (dd, *J* = 8.3, 4.4 Hz, 2H).

^{13}C NMR (126 MHz, Chloroform-d) δ 150.40 (2C), 147.95 (2C), 131.35 (2C), 129.24 (2C), 127.81 (2C), 125.36 (2C), 124.42 (2C), 121.80 (2C).

HRMS (GC-EI) *m/z* calculated for $C_{16}H_{10}N_2$ [M]: 230.083847; found: 230.084020.

quinolino[5,6-f]quinoline (2l): Prepared according to the general procedure (EtOAc), 15% yield as pale yellow solid.

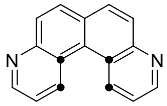

^{1}H NMR (501 MHz, Chloroform-*d*) δ 9.28–9.20 (m, 2H), 9.06 (dd, *J* = 4.3, 1.5 Hz, 2H), 8.23 (d, *J* = 8.8 Hz, 2H), 8.14 (d, *J* = 8.8 Hz, 2H), 7.63 (dd, *J* = 8.6, 4.2 Hz, 2H).

^{13}C NMR (126 MHz, Chloroform-d) δ 149.54 (2C), 148.93 (2C), 135.10 (2C), 130.82 (2C), 130.60 (2C), 129.62 (2C), 126.47 (2C), 125.26 (2C).

HRMS (ESI) *m/z* calculated for $C_{16}H_{11}N_2$ $[M+H]^+$: 231.091672; found: 231.091710.

5. Intermediate

5.1. Procedure:

A round-bottom flask was charged with a magnetic stir-bar and starting material **1a** (1.0 equiv.). Then toluene (0,025M) and *p*-TSA (10.0 equiv.) were added. The reaction mixture was stirred at 110 °C under argon for 24h. After cooling to room temperature, the reaction mixture was treated with 1 N NaOH and EtOAc. After 10 min stirring at room temperature, the mixture was extracted EtOAc (5x7 mL). The organic phase was dried with Na_2SO_4, filtered, and concentrated under reduced pressure. The residue was purified by column chromatography on silica gel (hexanes/EtOAc = 4.1; *v/v*) to give 1',2',3',4'-tetrahydro-2,3'-bibenzo[f]quinoline (**4**) in 20% yield as yellow solid.

5.2. Analytical data:

1',2',3',4'-tetrahydro-2,3'-bibenzo[f]quinoline (**4**):

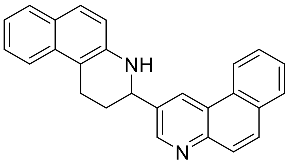

^{1}H NMR (501 MHz, Chloroform-*d*) δ 9.06–9.02 (m, 2H), 8.66–8.61 (m, 1H), 8.05 (d, *J* = 9.0 Hz, 1H), 8.00 (d, *J* = 9.1 Hz, 1H), 7.95 (dd, *J* = 7.4, 1.8 Hz, 1H), 7.78 (d, *J* = 8.4 Hz, 1H), 7.74 (dd, *J* = 8.1, 1.3 Hz, 1H), 7.72–7.65 (m, 2H), 7.62 (d, *J* = 8.7 Hz, 1H), 7.47 (ddd, *J* = 8.3, 6.8, 1.3 Hz, 1H), 7.30–7.26 (m, 1H), 6.94 (d, *J* = 8.7 Hz, 1H), 4.80 (dd, *J* = 9.7, 3.1 Hz, 1H), 4.35 (s, 1H), 3.30–3.11 (m, 2H), 2.50–2.41 (m, 1H), 2.40-2.28 (m, 1H).

^{13}C NMR (126 MHz, Chloroform-d) δ 148.53, 141.58, 137.49, 133.23, 131.87, 131.58, 131.16, 129.40, 128.81, 128.51, 128.18, 127.65, 127.60, 127.27, 126.52, 125.42, 123.13, 122.78, 122.14, 121.48, 118.11, 111.54, 54.21, 31.47, 29.70, 22.49.

HRMS (ESI) *m/z* calculated for $C_{26}H_{21}N_2$ $[M+H]^+$: 361.169922; found: 361.169890.

6. NMR spectra

^{1}H and ^{13}C NMR traces of 6:

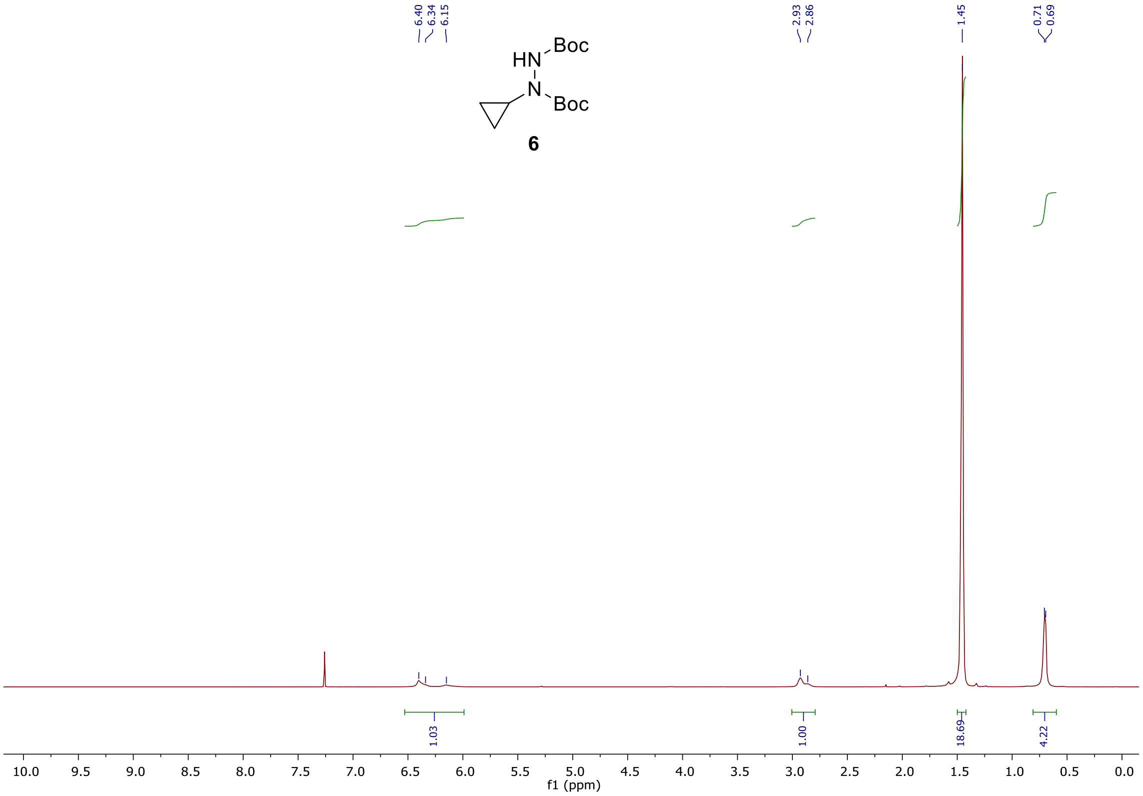

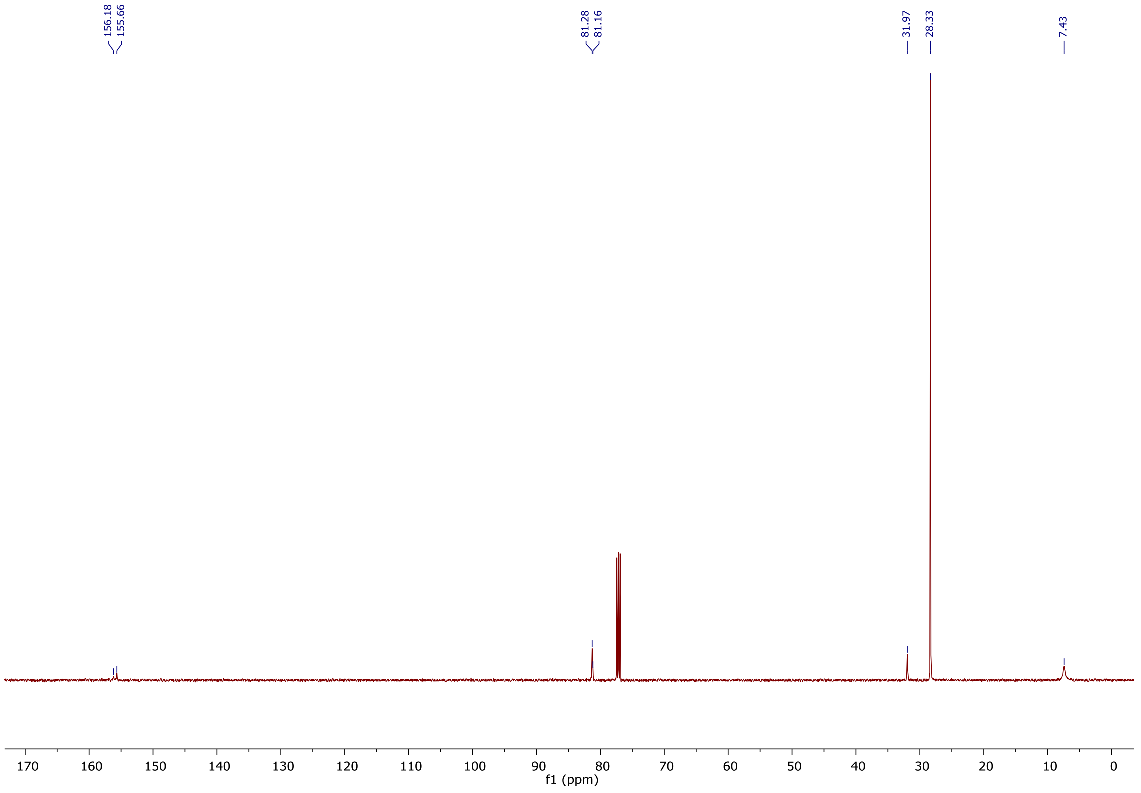

^{1}H and ^{13}C NMR traces of 1a:

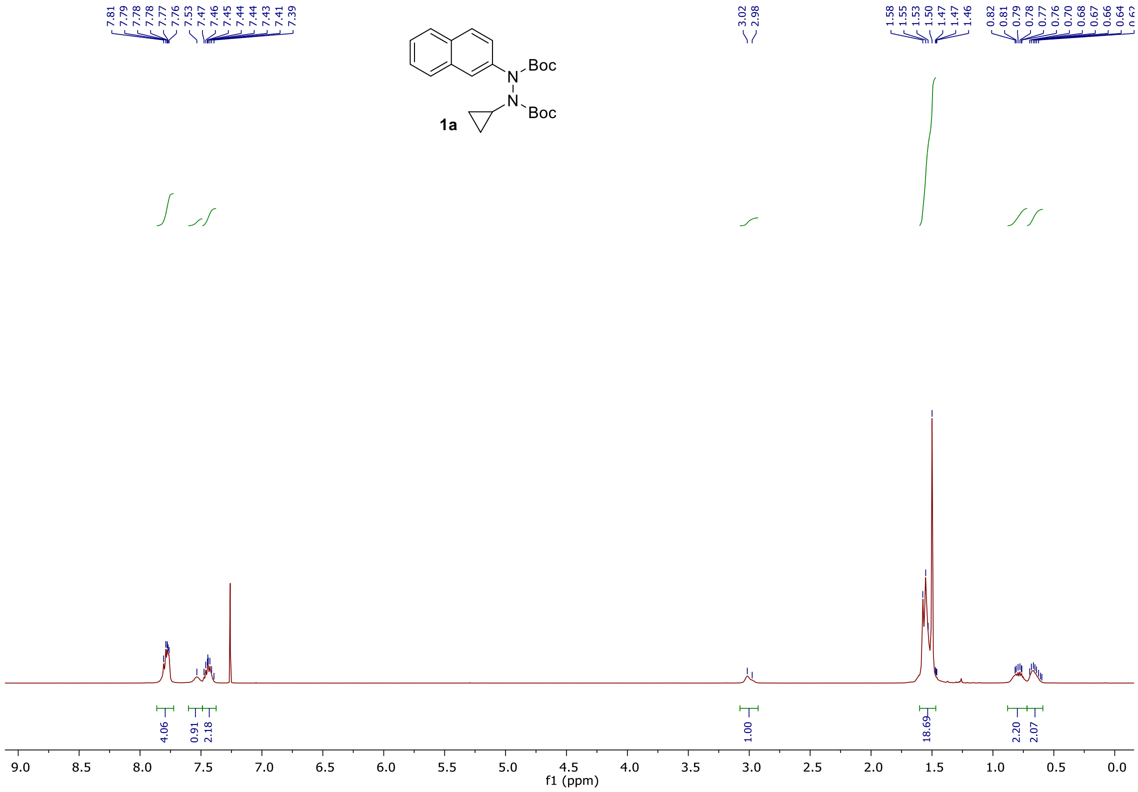

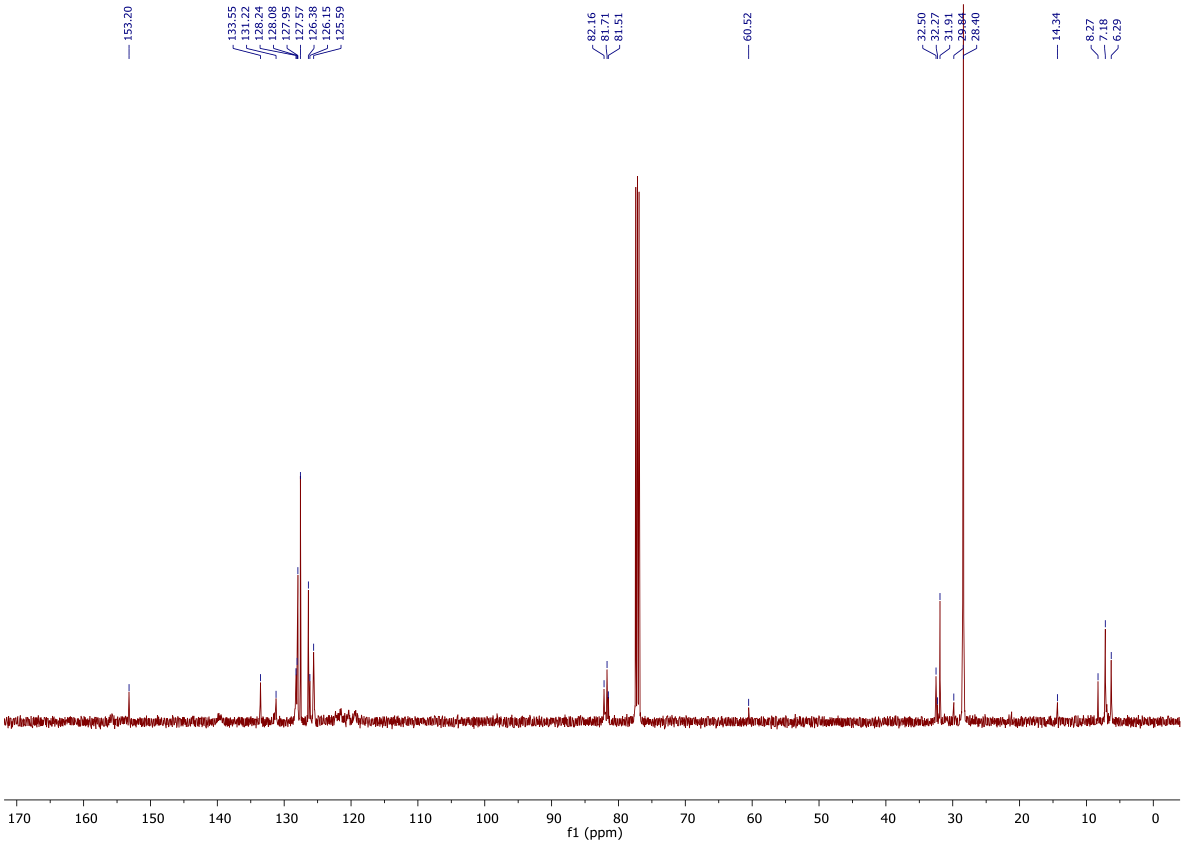

^{1}H and ^{13}C NMR traces of 1b:

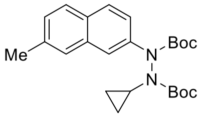

1b

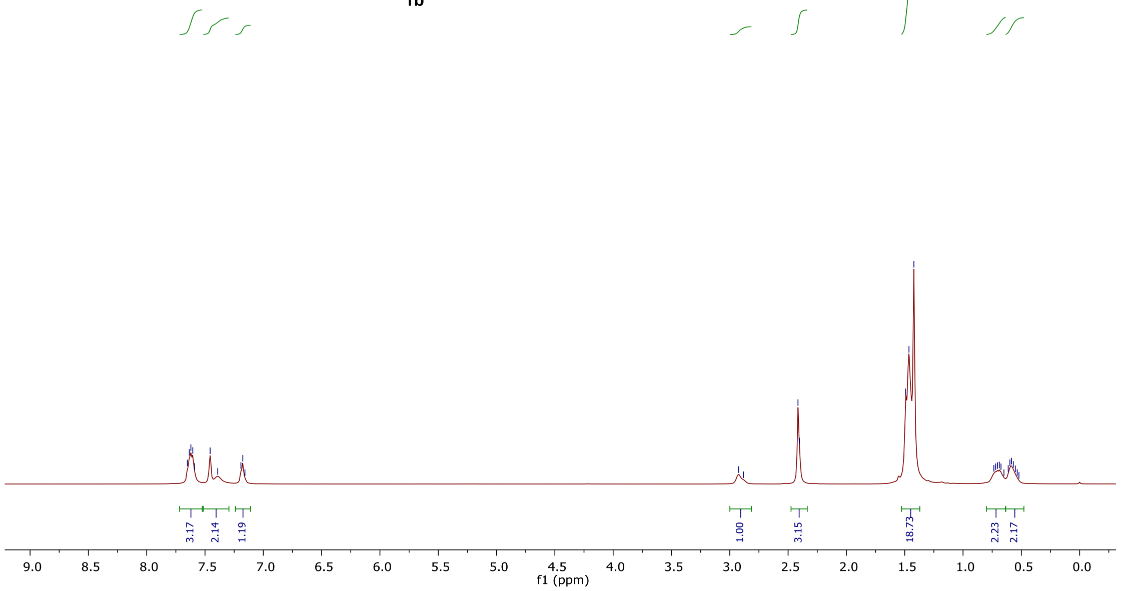

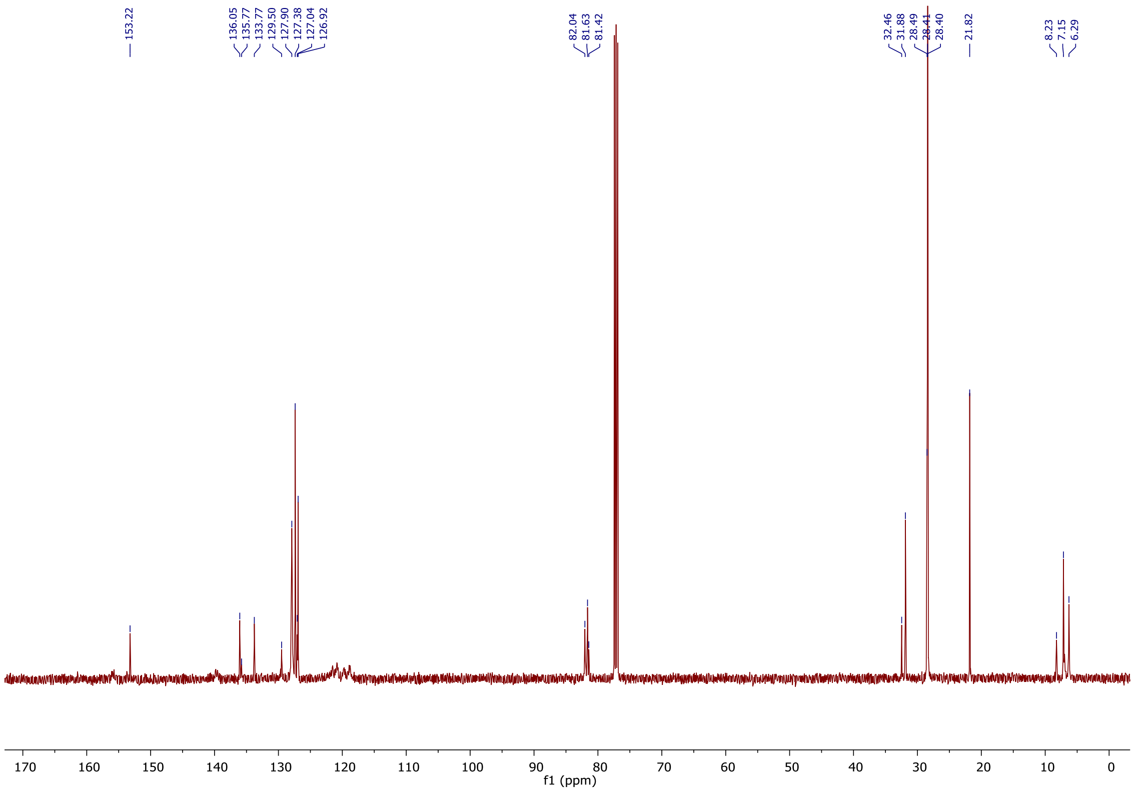

^{1}H and ^{13}C NMR traces of 1c:

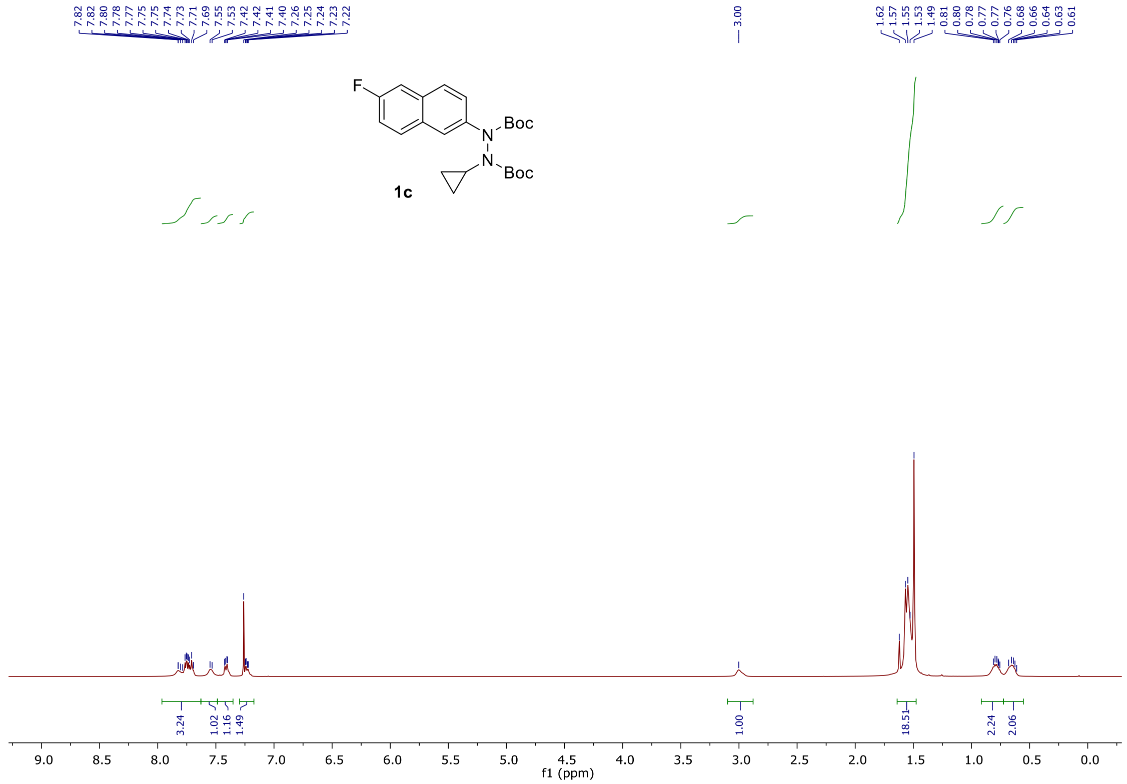

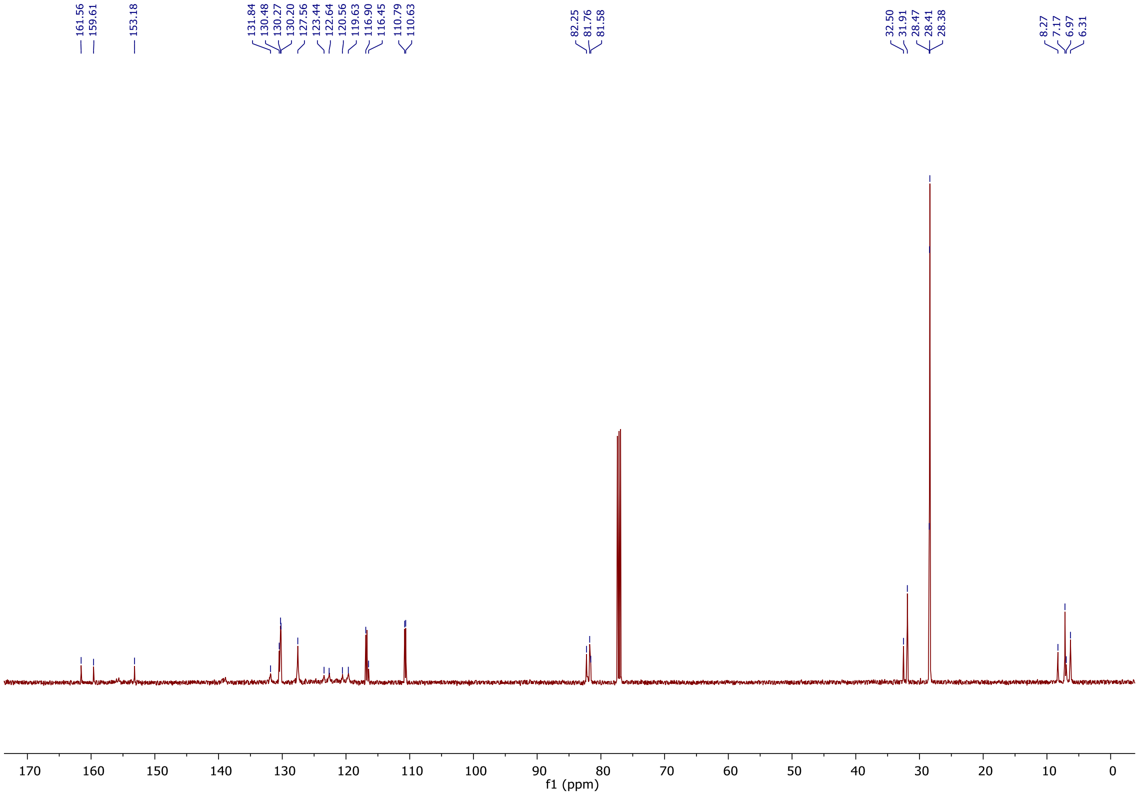

^{1}H and ^{13}C NMR traces of 1d:

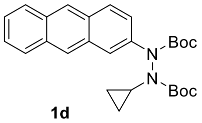

1d

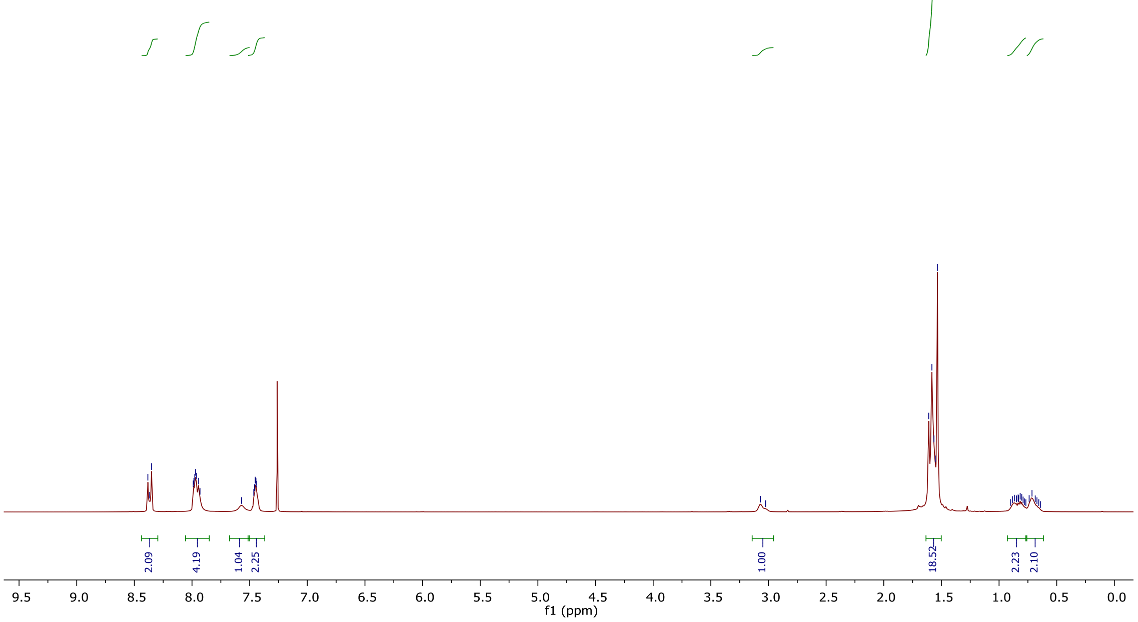

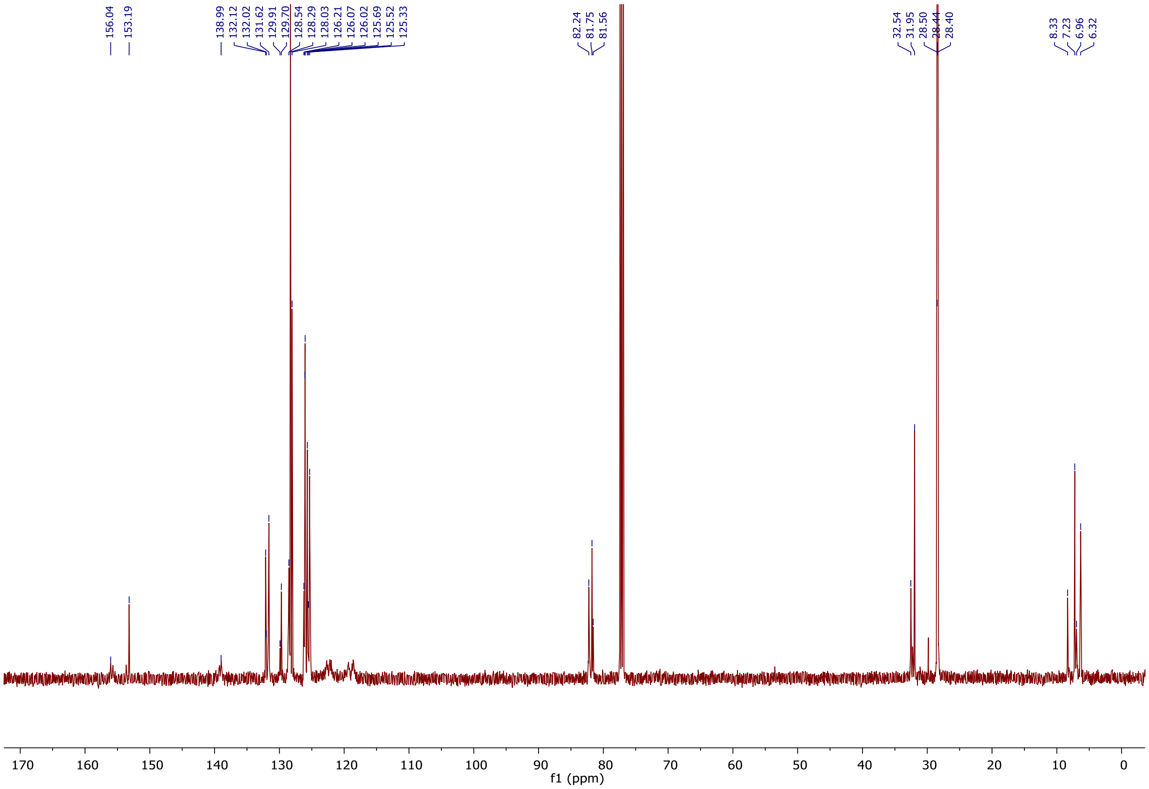

¹H and ¹³C NMR traces of 1e:

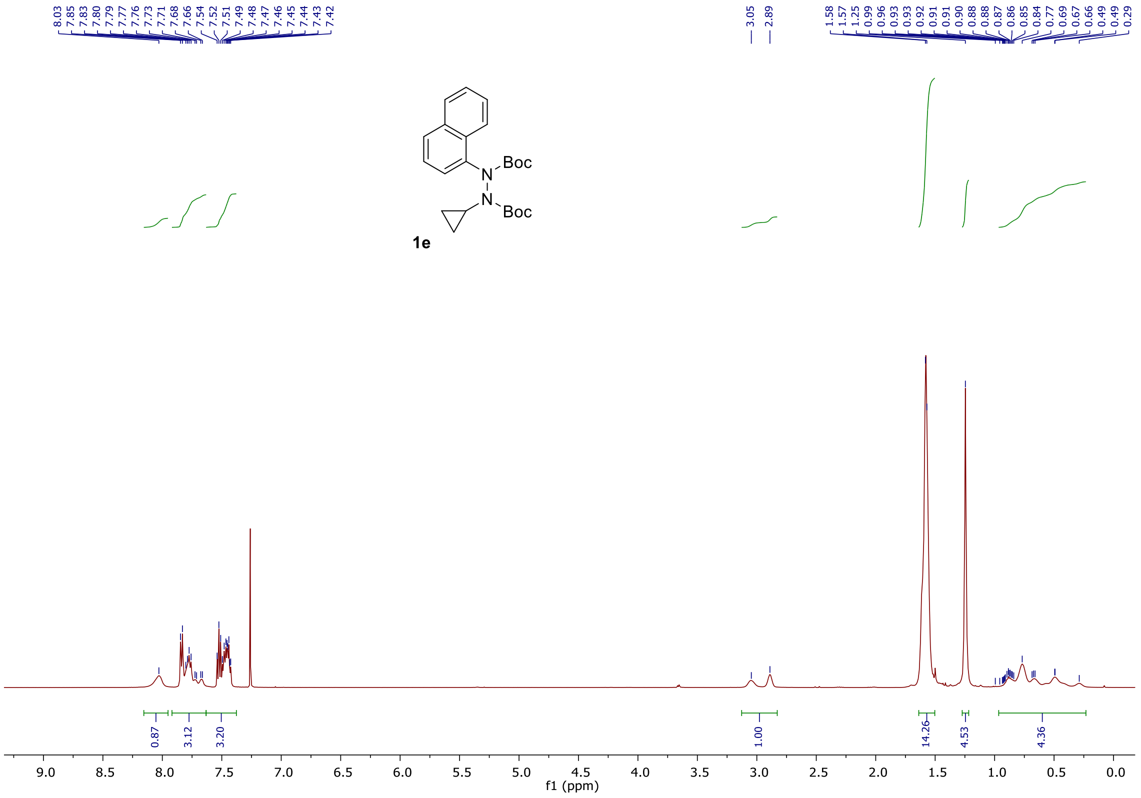

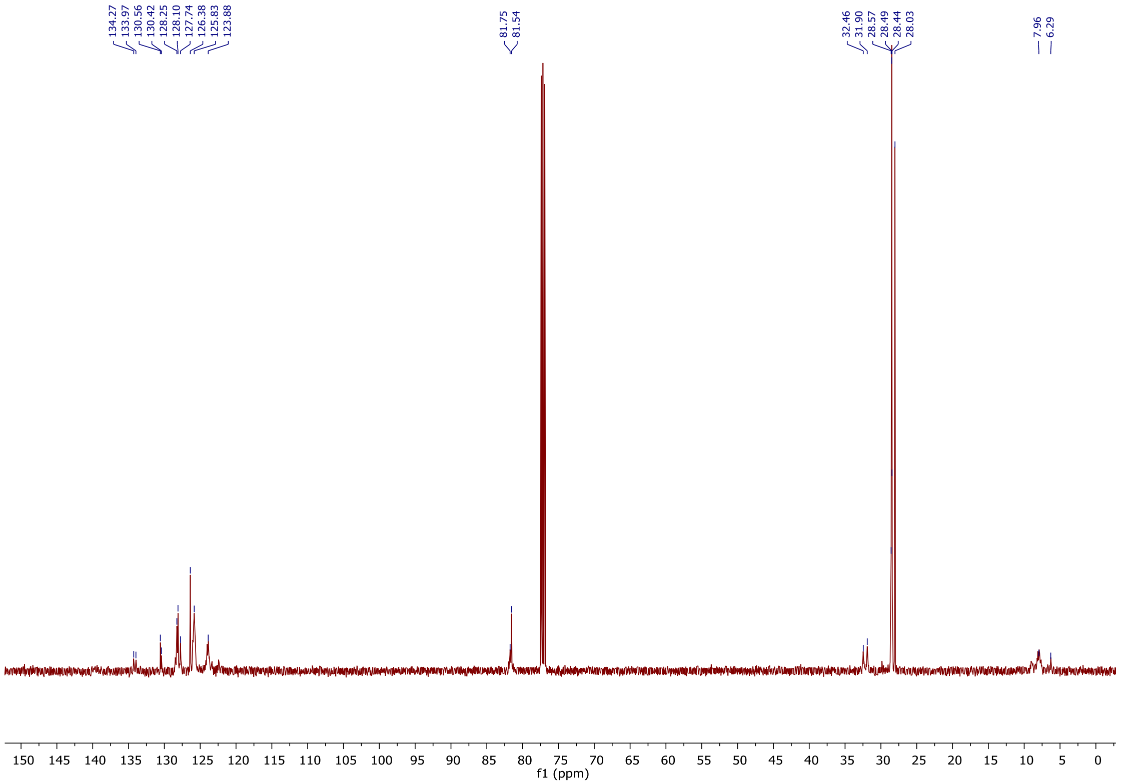

¹H and ¹³C NMR traces of 1f:

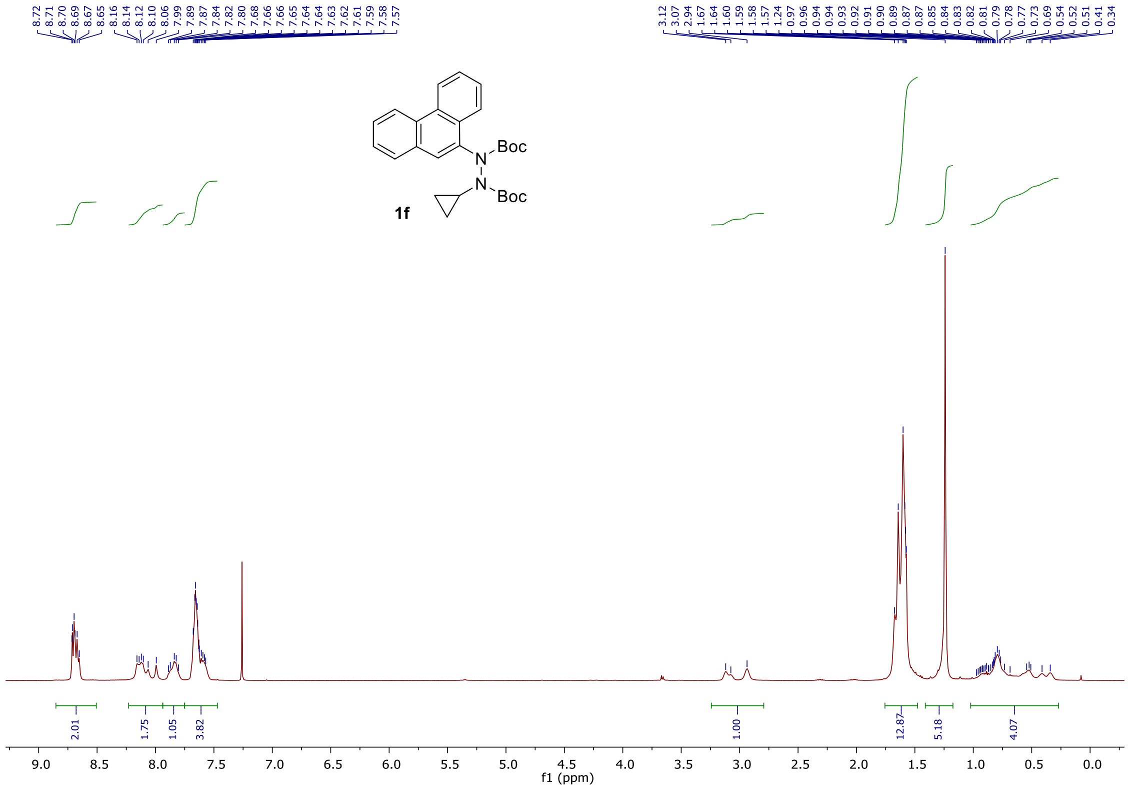

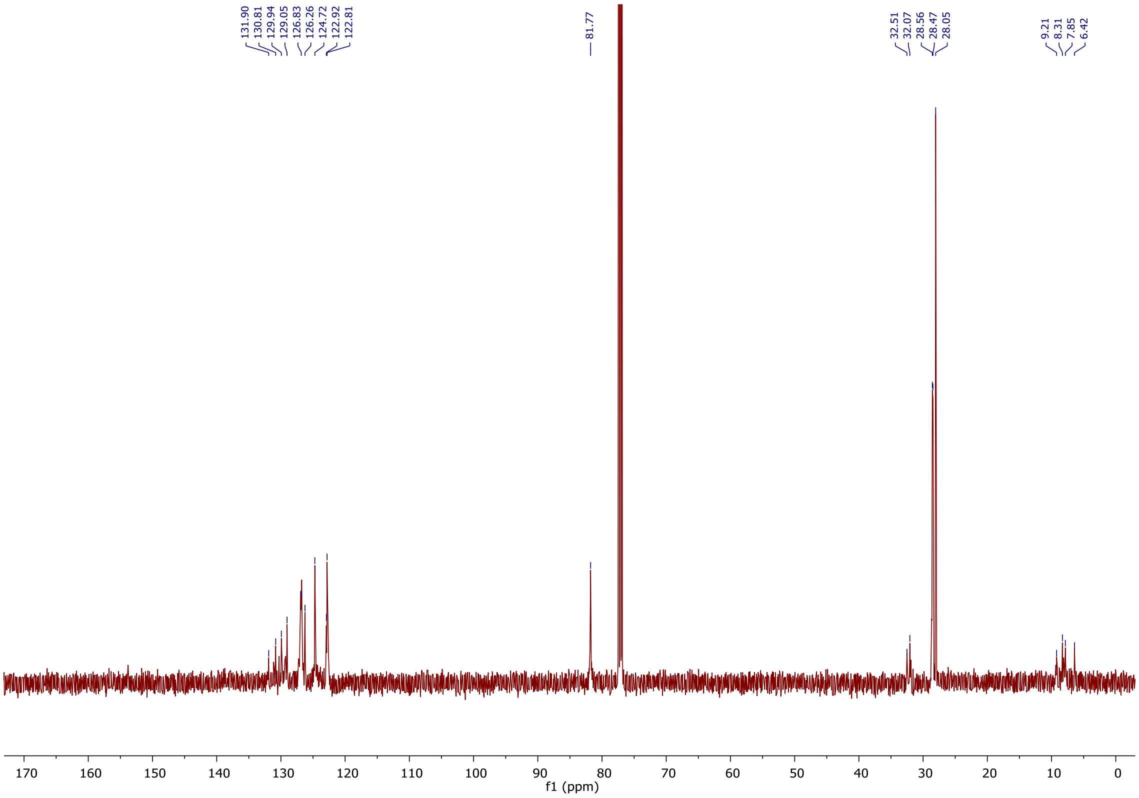

^{1}H and ^{13}C NMR traces of 1g:

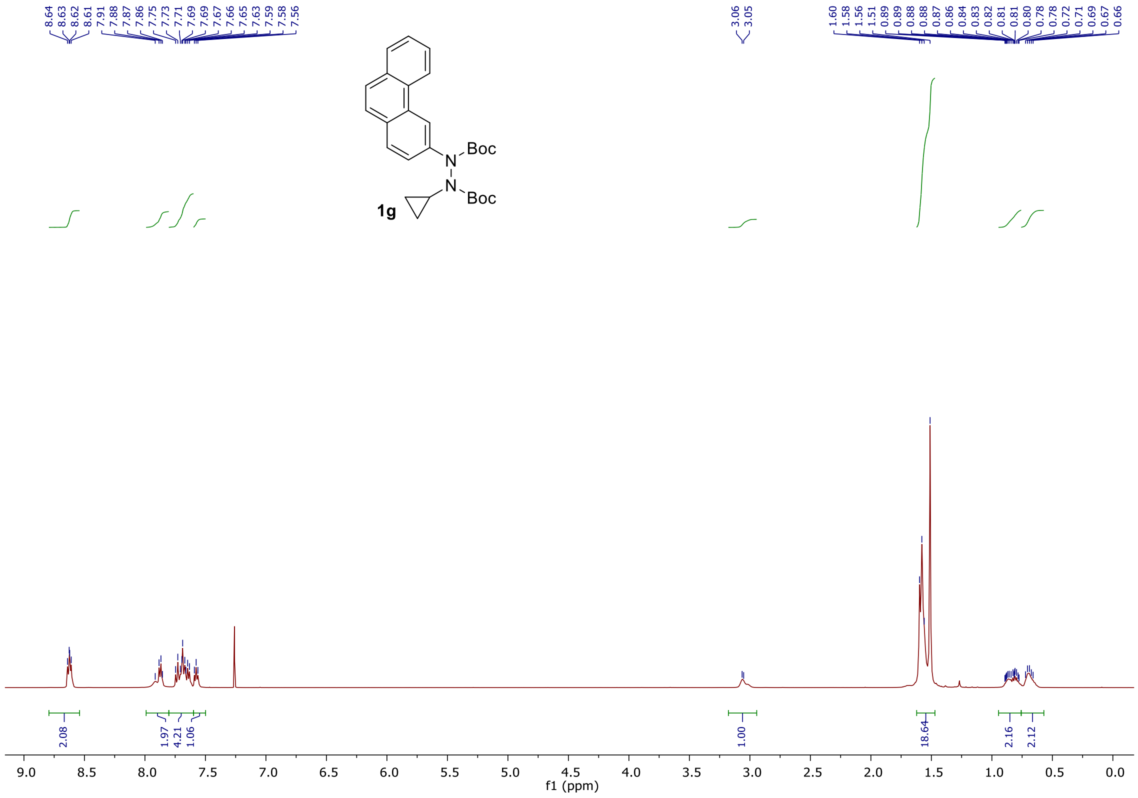

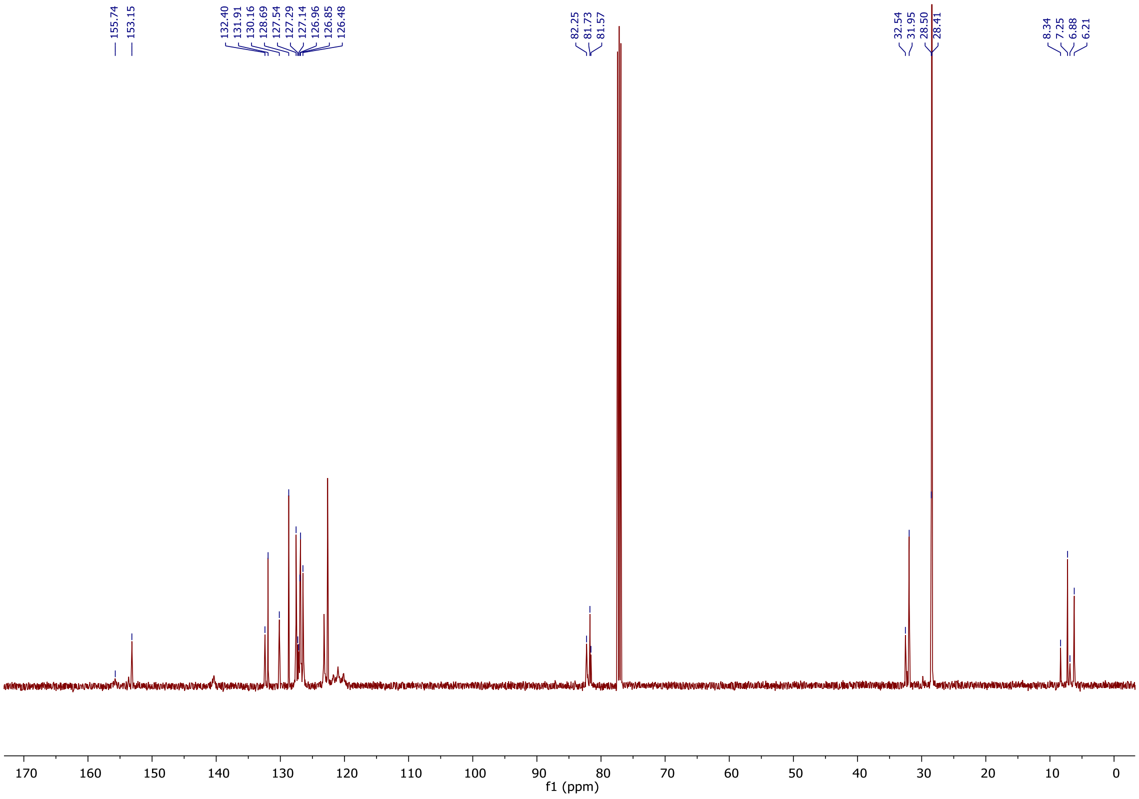

^{1}H and ^{13}C NMR traces of 1h:

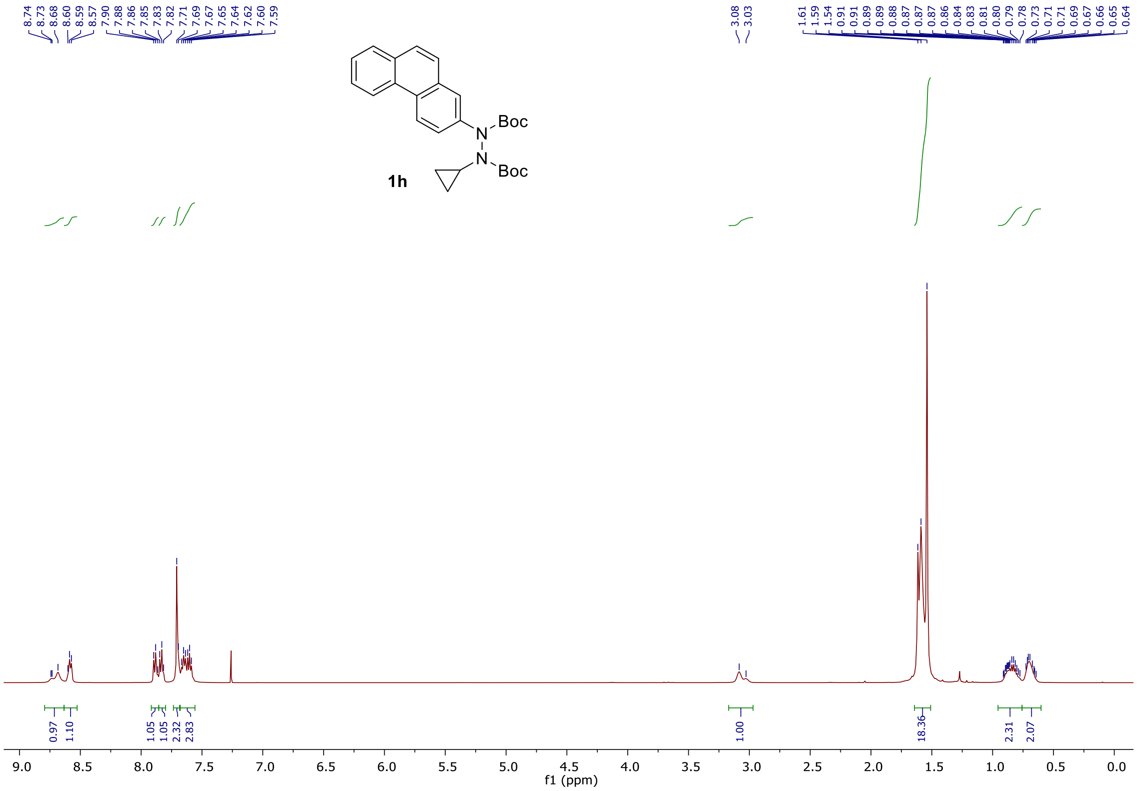

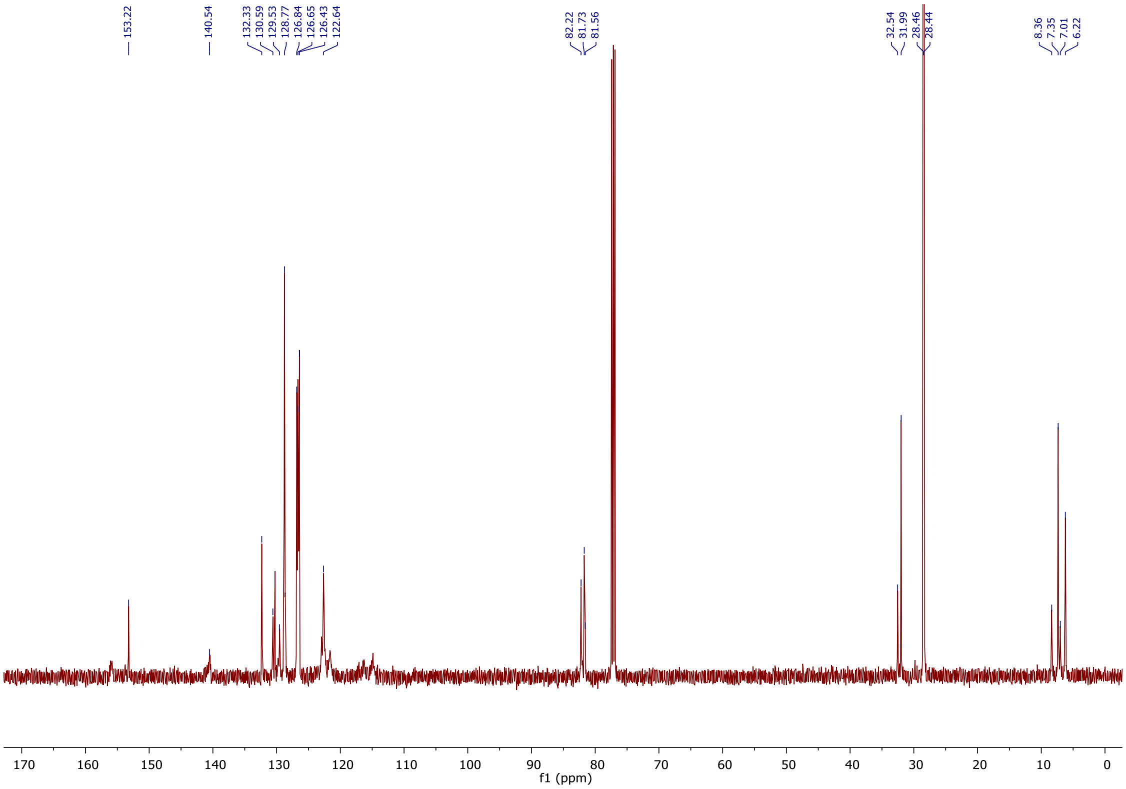

^{1}H and ^{13}C NMR traces of 1i:

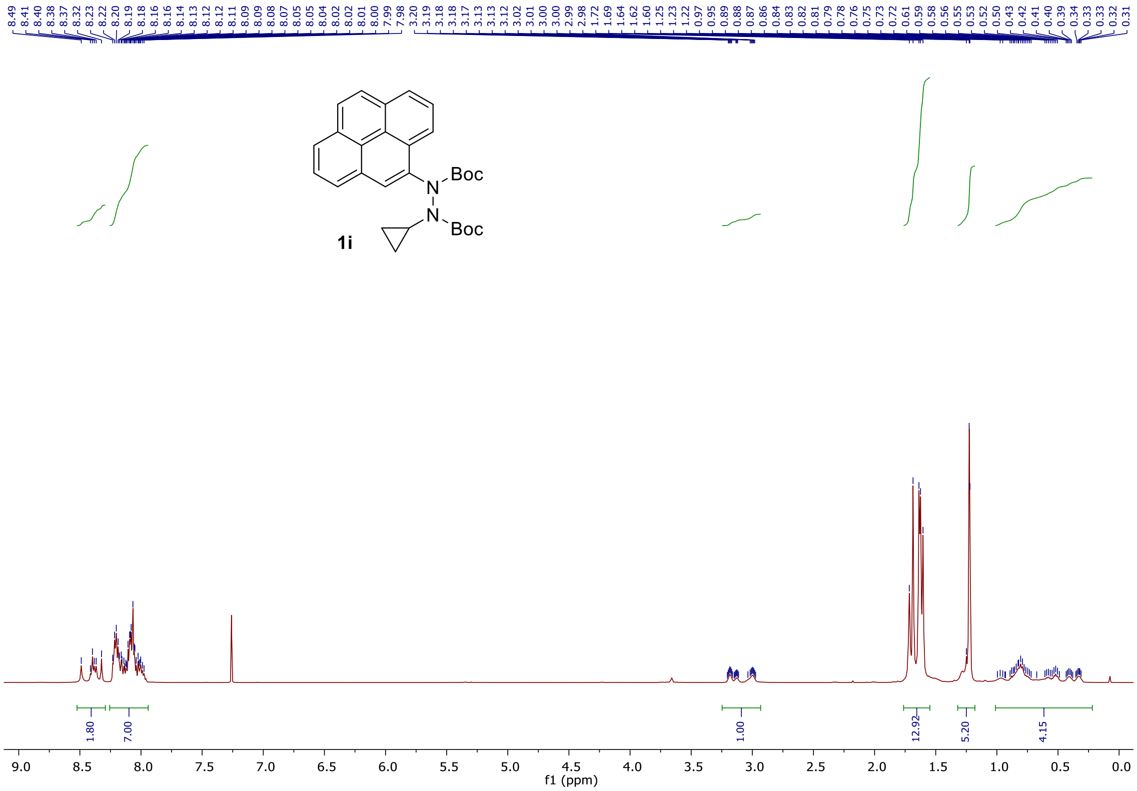

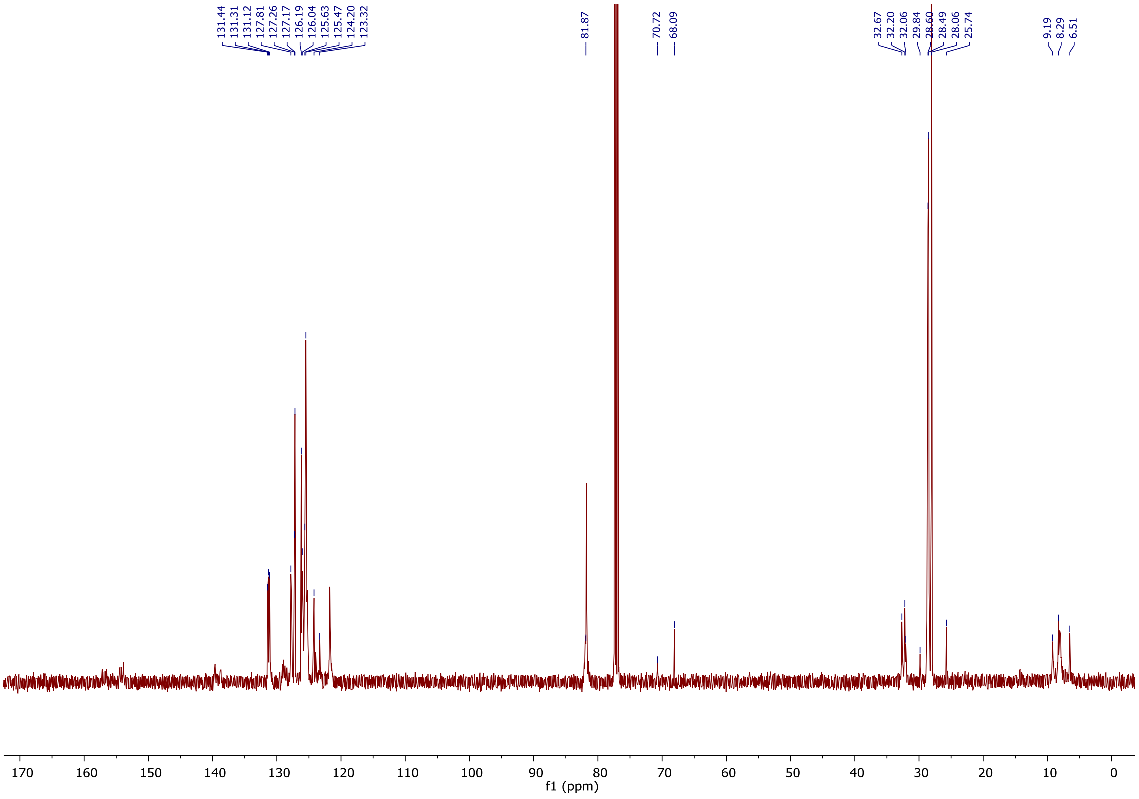

^{1}H and ^{13}C NMR traces of 1j:

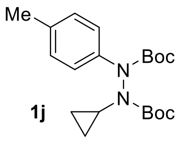

1j

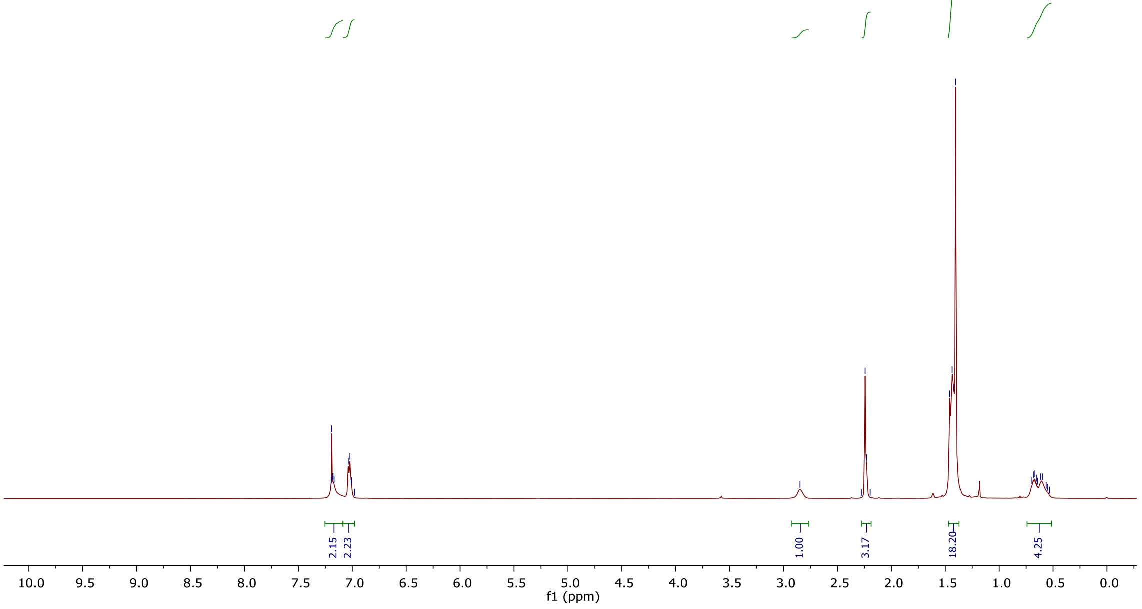

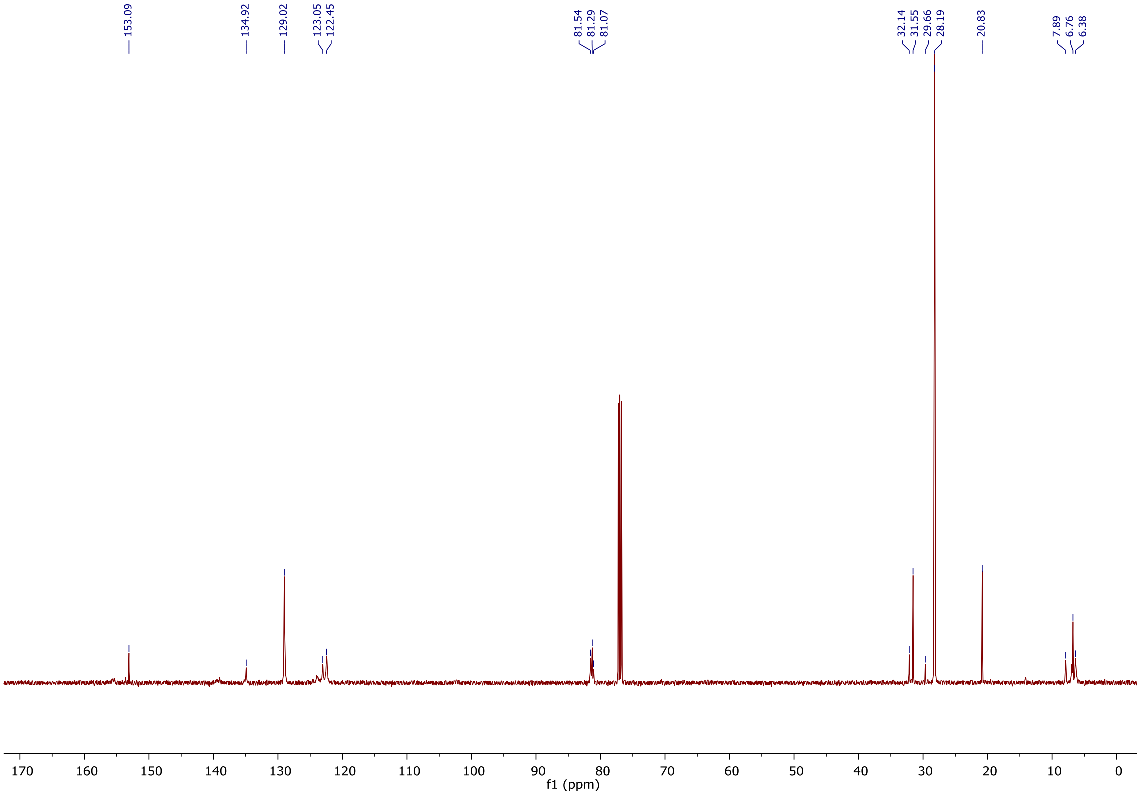

^{1}H and ^{13}C NMR traces of 1k:

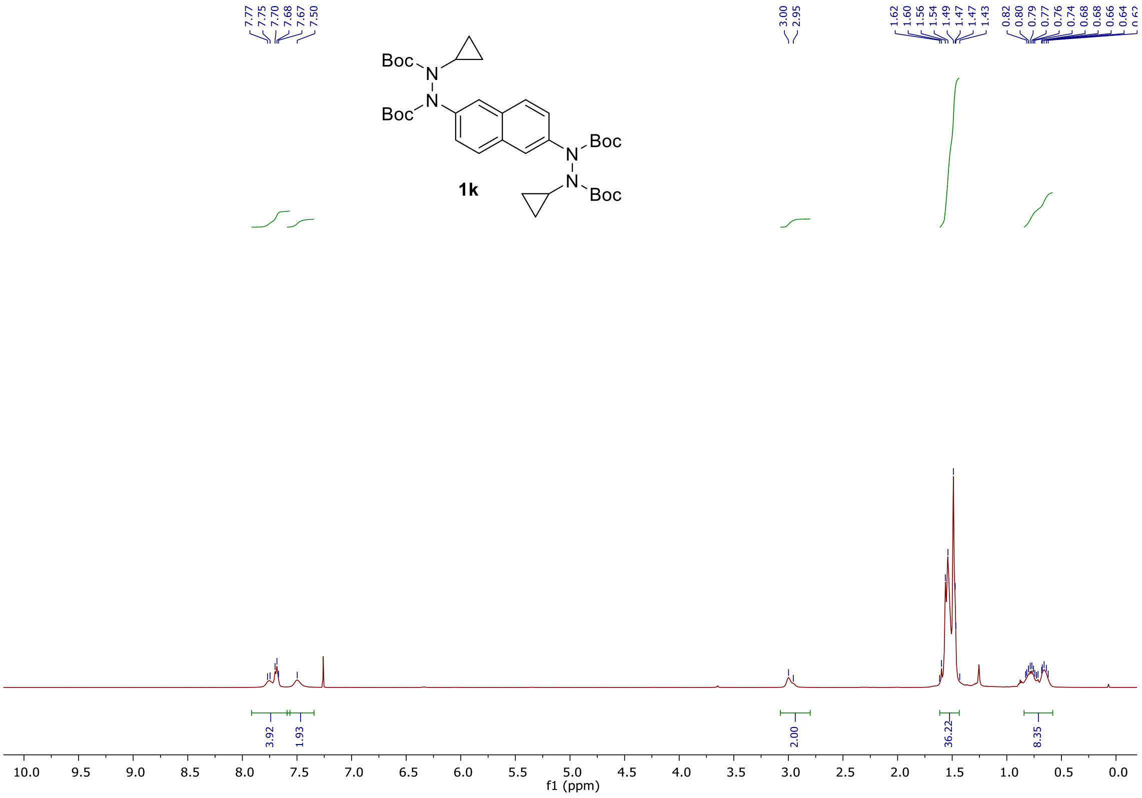

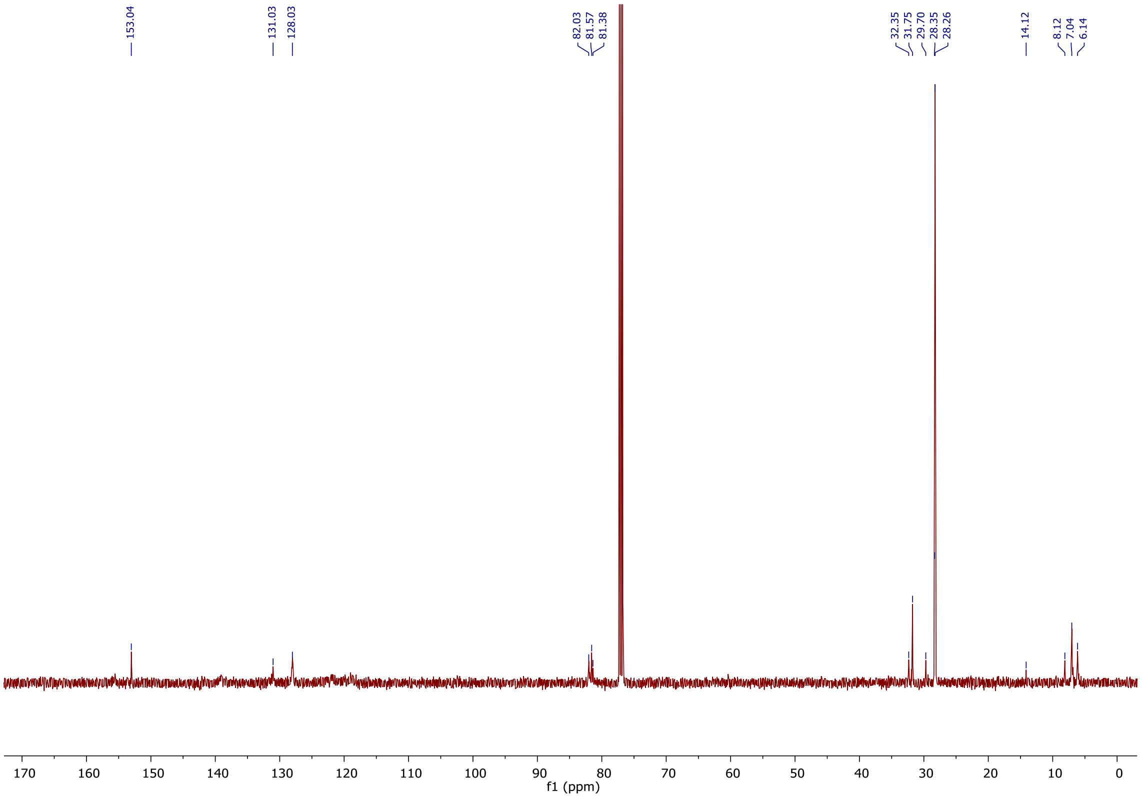

^{1}H and ^{13}C NMR traces of 1l:

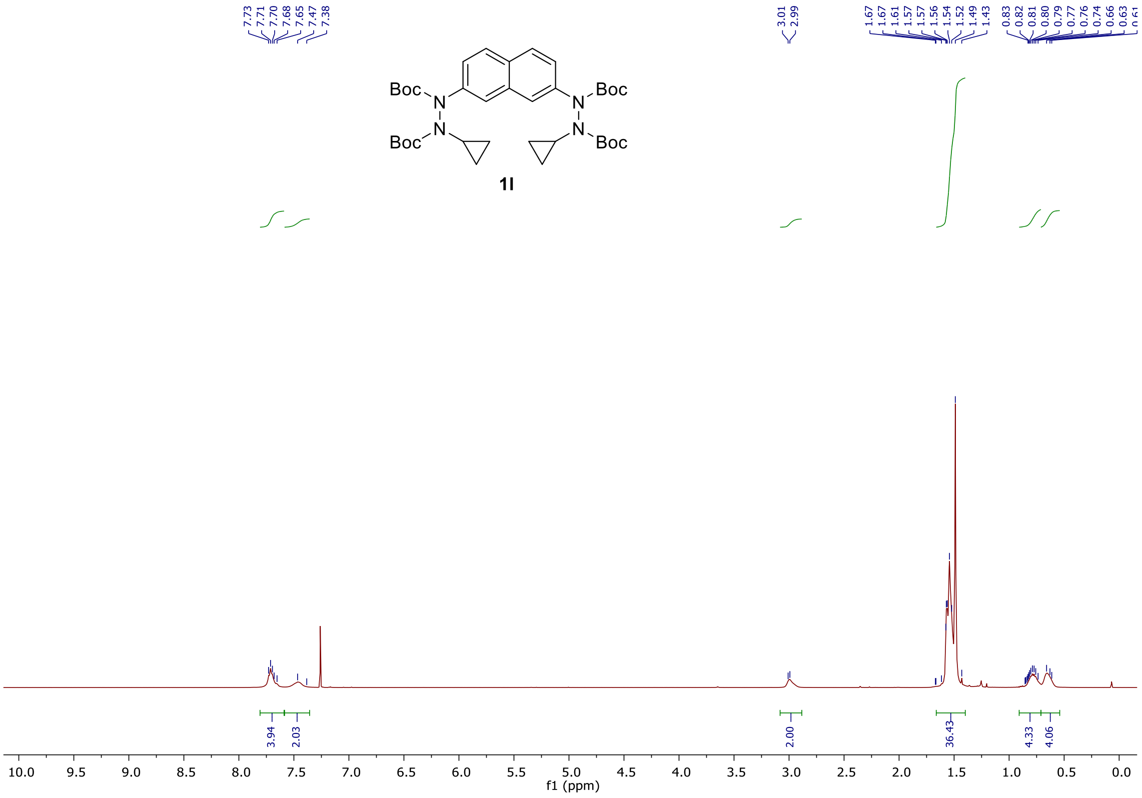

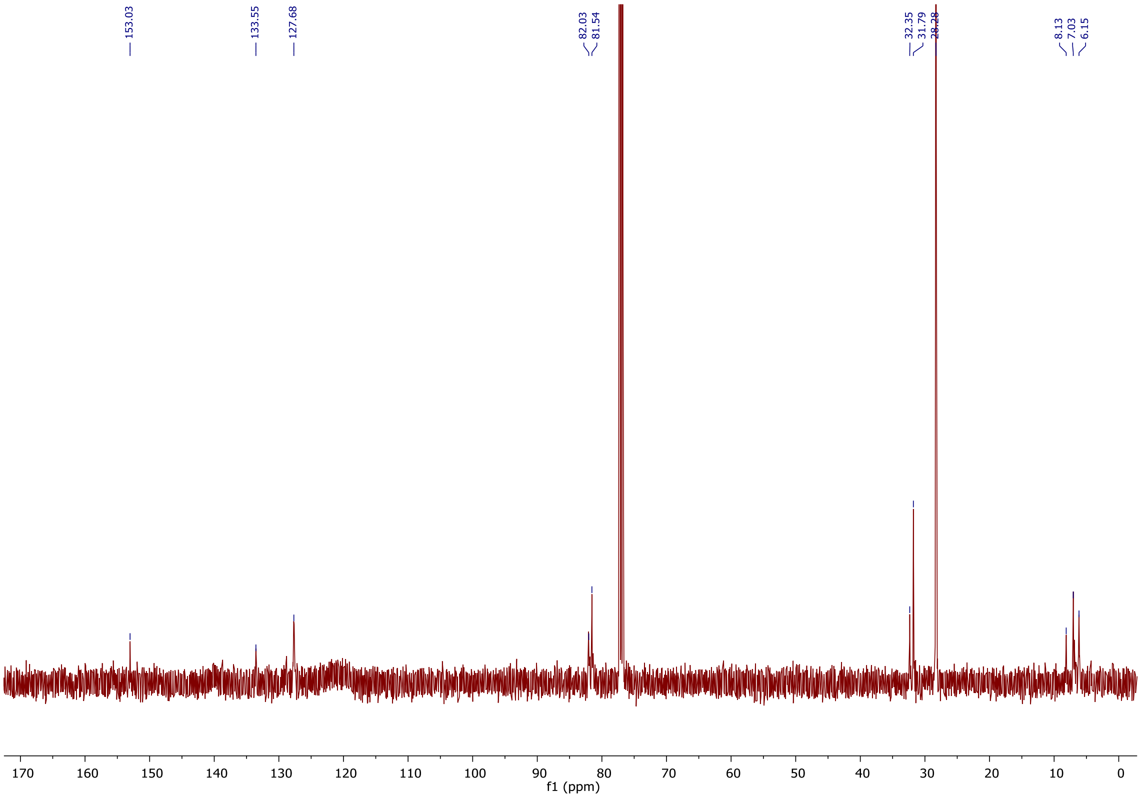

^{1}H and ^{13}C NMR traces of 2a:

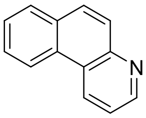

2a

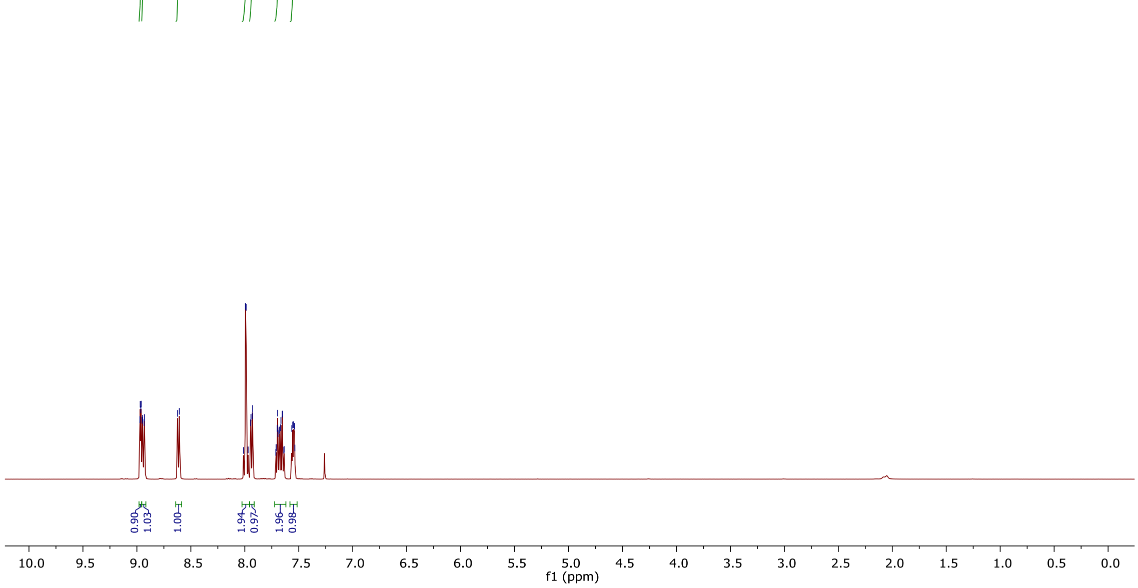

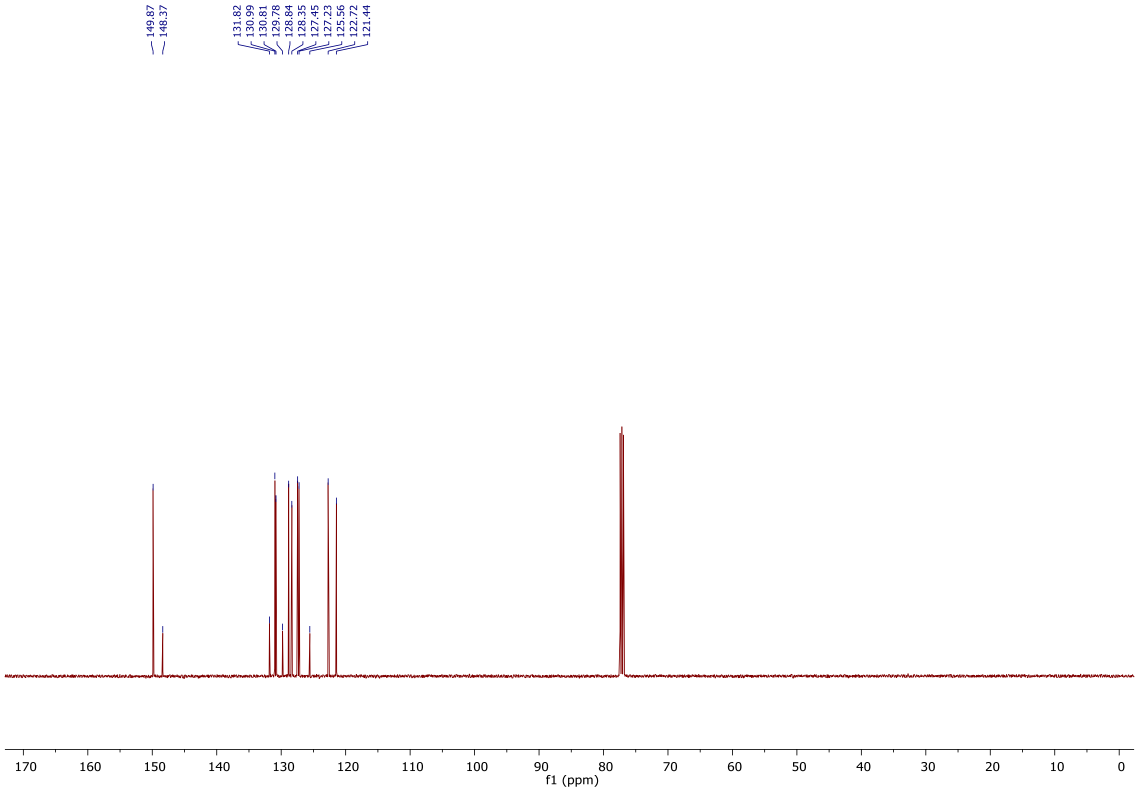

^{1}H and ^{13}C NMR traces of 2b:

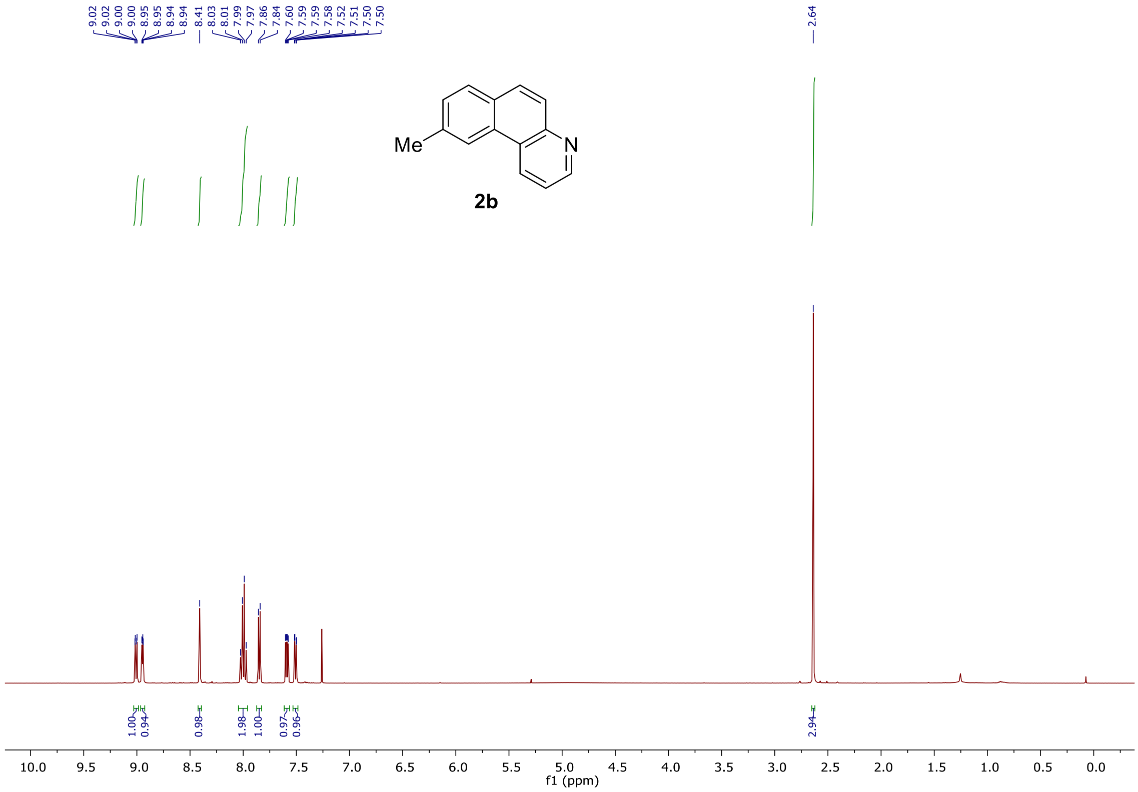

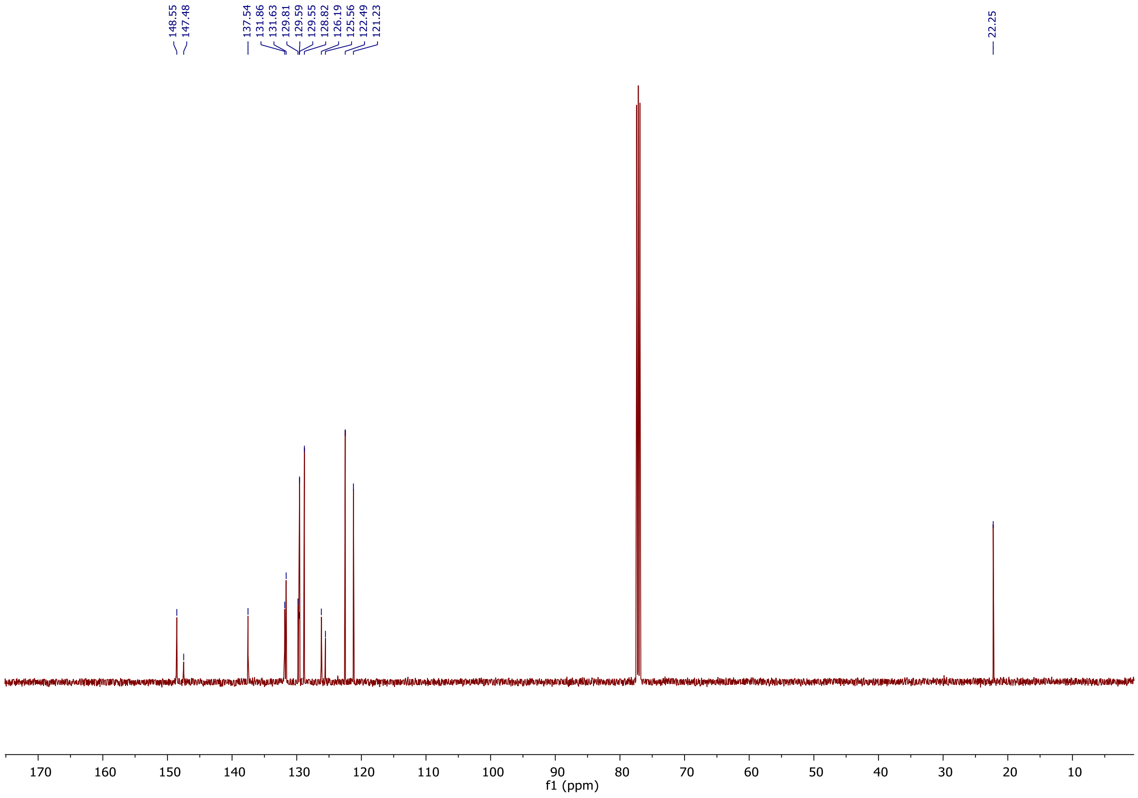

^{1}H, ^{19}F and ^{13}C NMR traces of 2c:

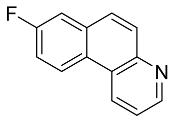

2c

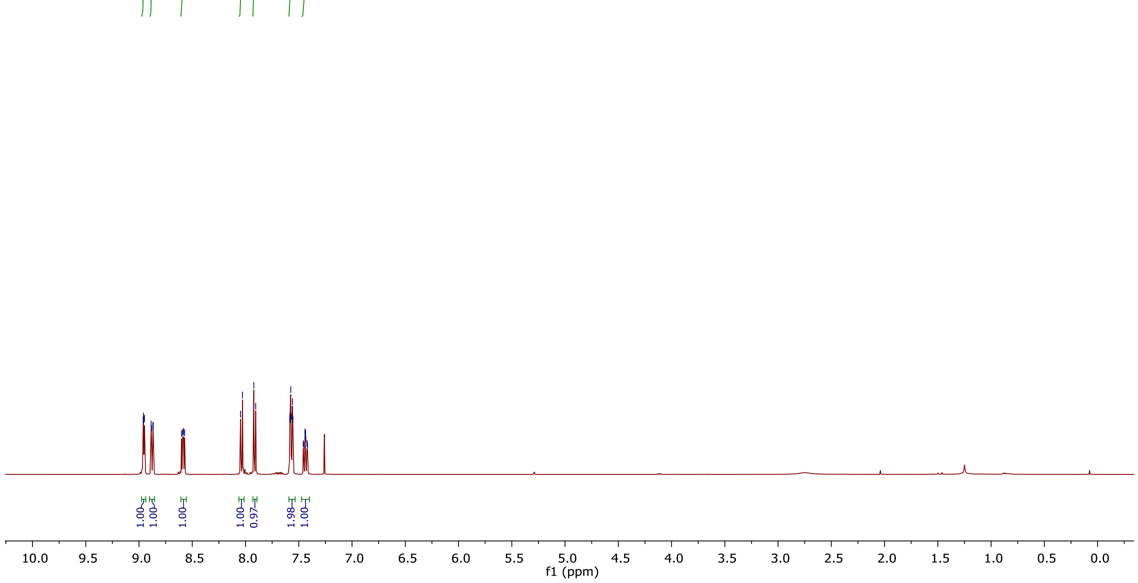

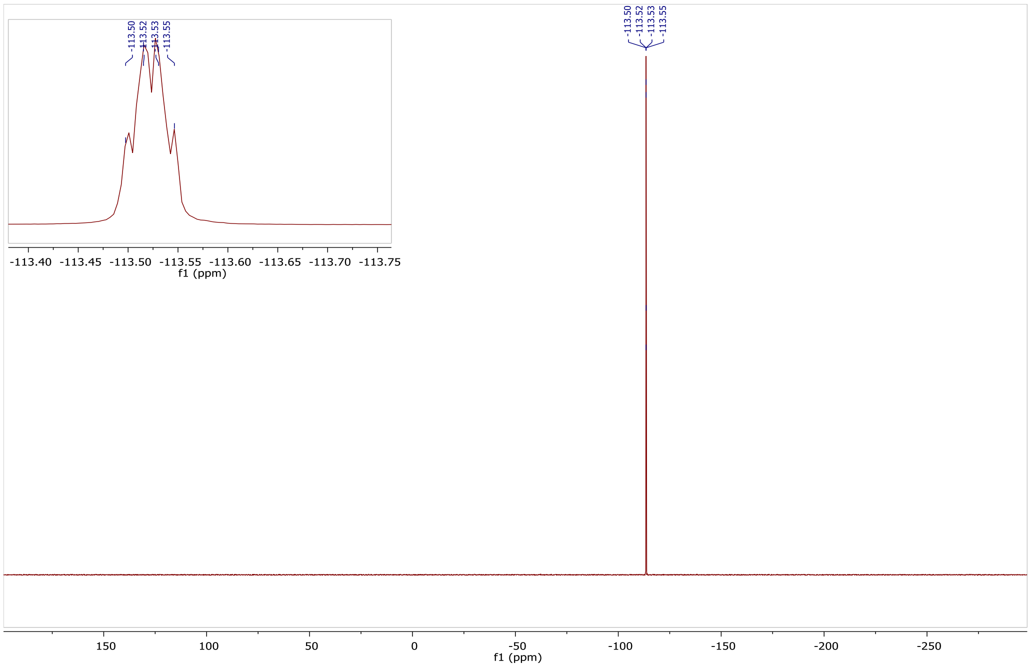

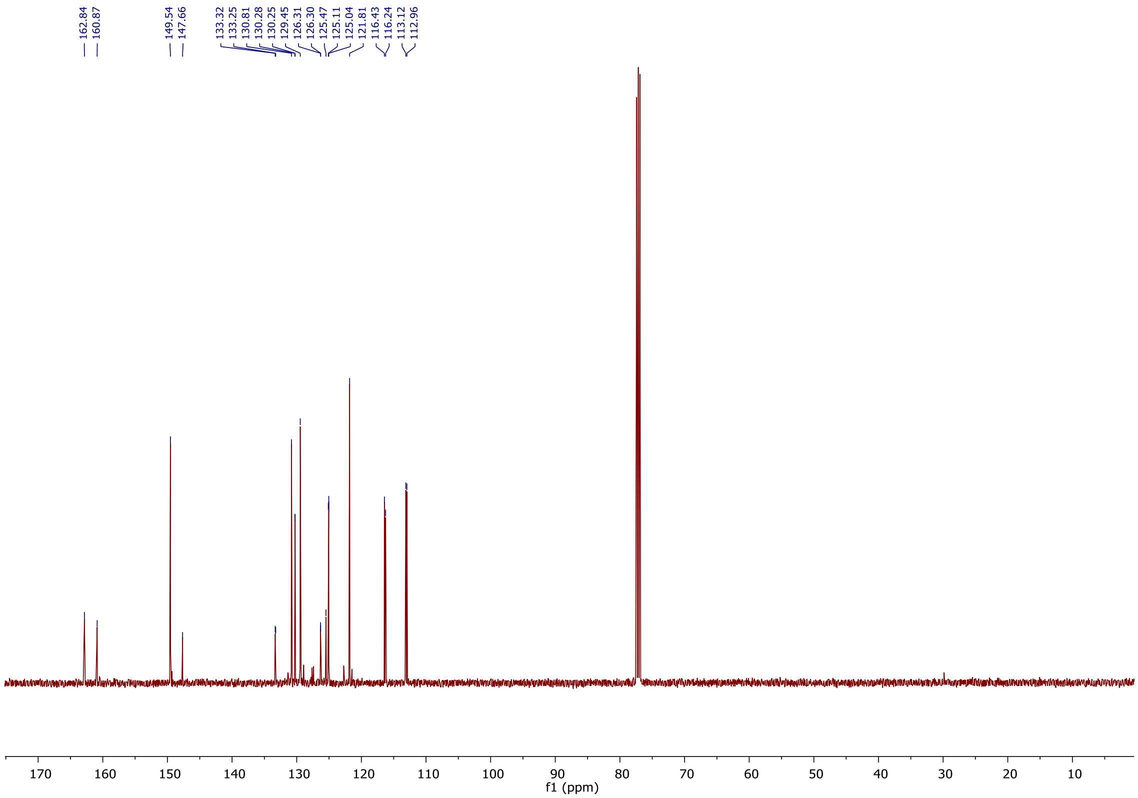
162.84
160.87
149.54
147.66
133.32
133.25
130.81
130.28
130.25
129.45
126.31
126.30
125.47
125.11
125.04
121.81
116.43
116.24
113.12
112.96
170
160
150
140
130
120
110
100
90
80
70
60
50
40
30
20
10
f1 (ppm)

^{1}H and ^{13}C NMR traces of 2d:

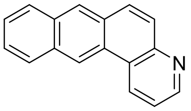

2d

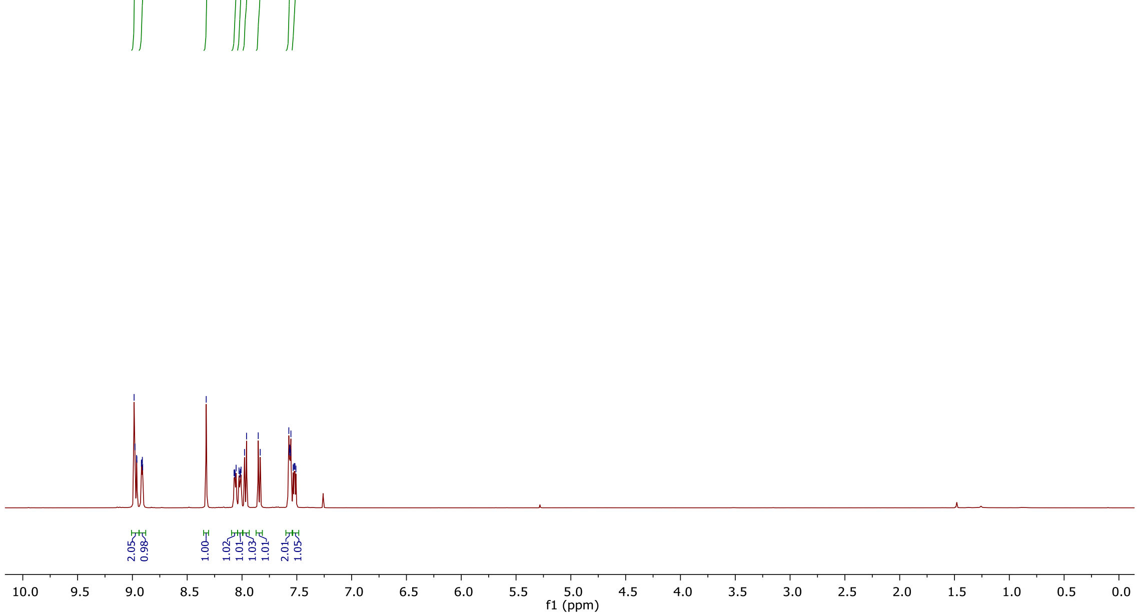

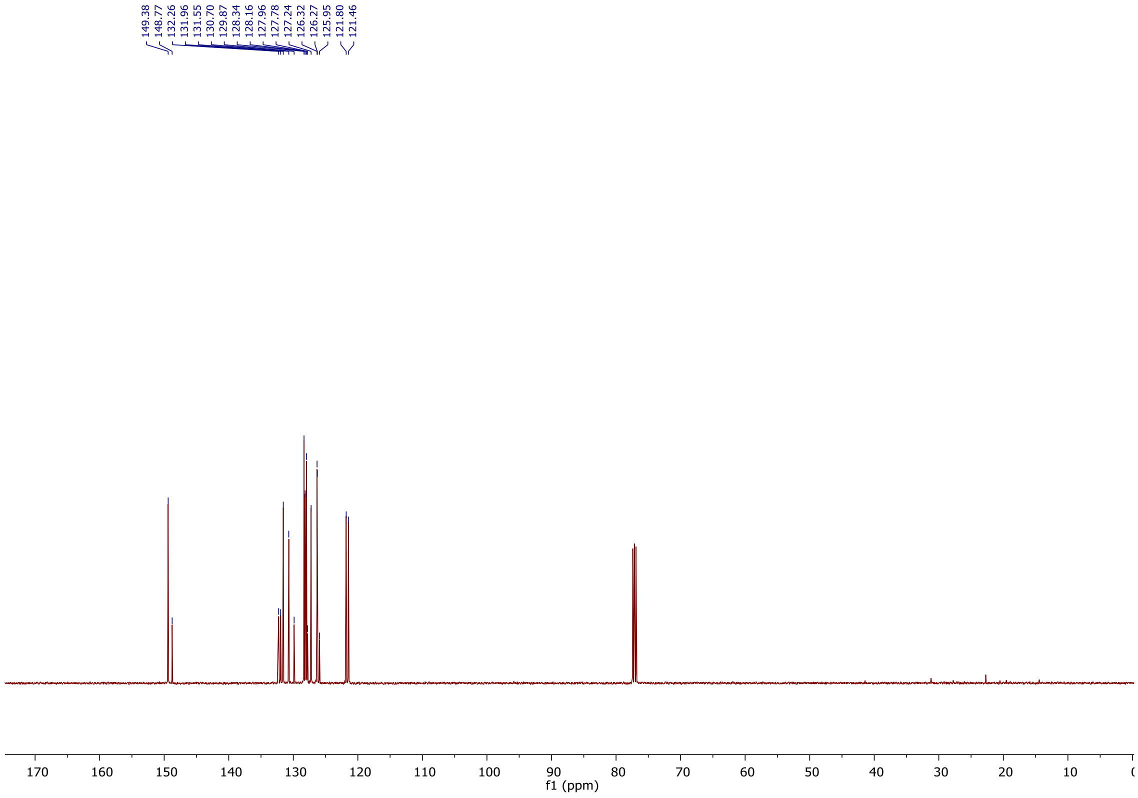

^{1}H and ^{13}C NMR traces of 2e:

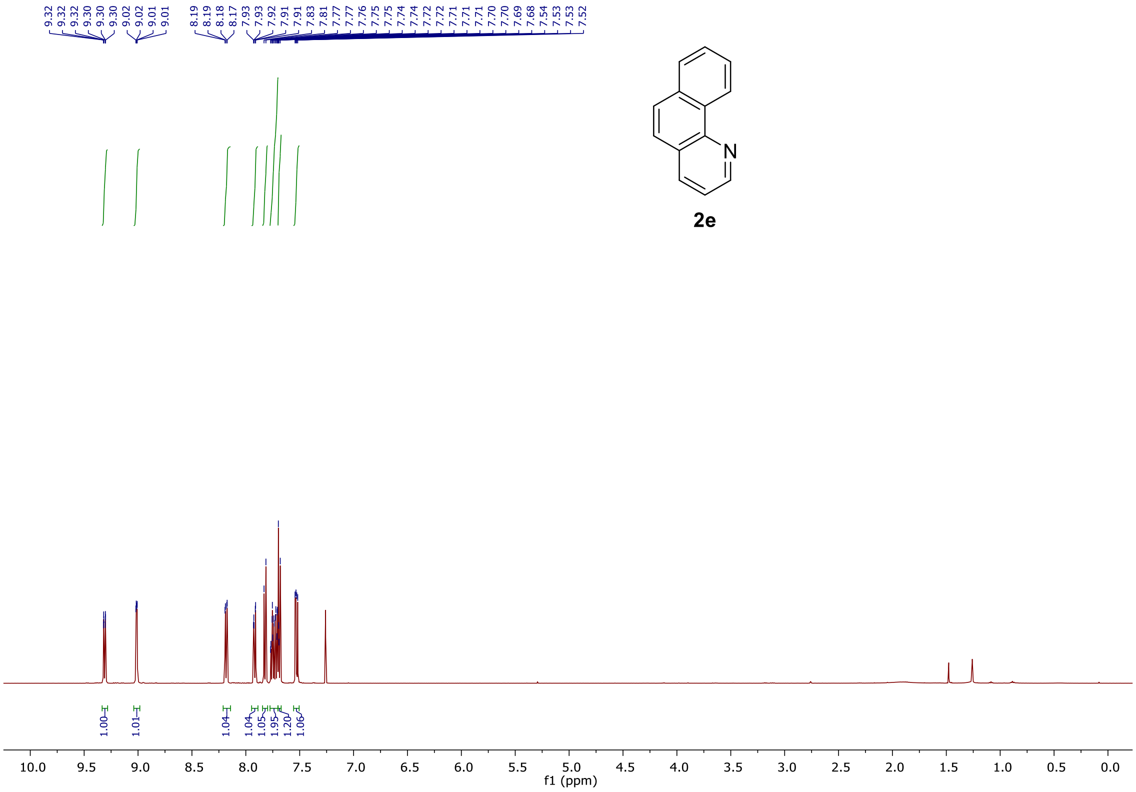

2e

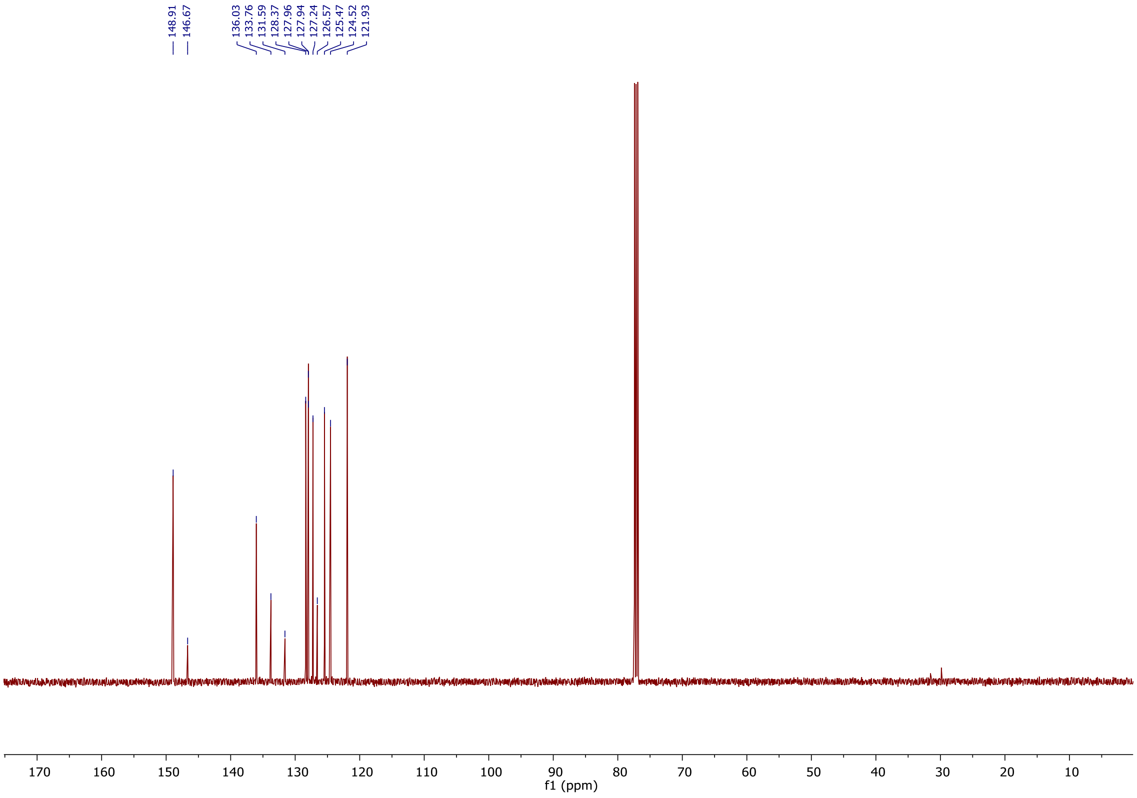

^{1}H and ^{13}C NMR traces of 2f:

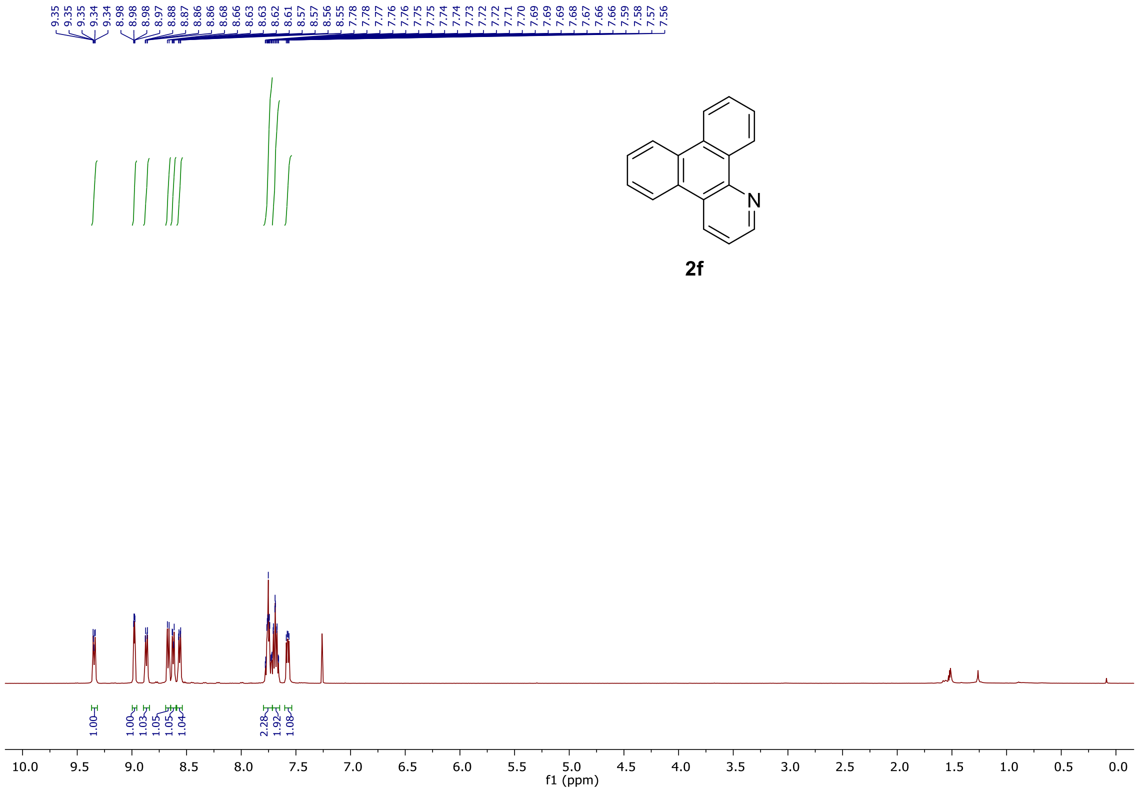

2f

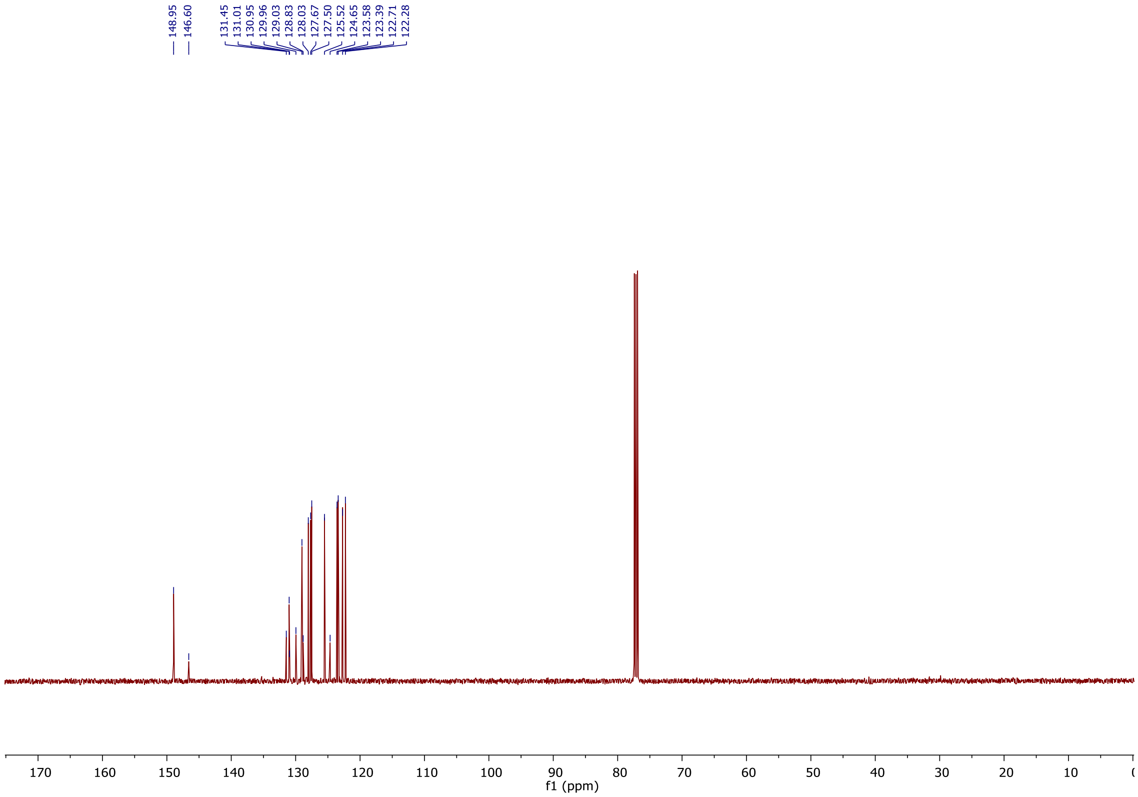

^{1}H and ^{13}C NMR traces of 2g:

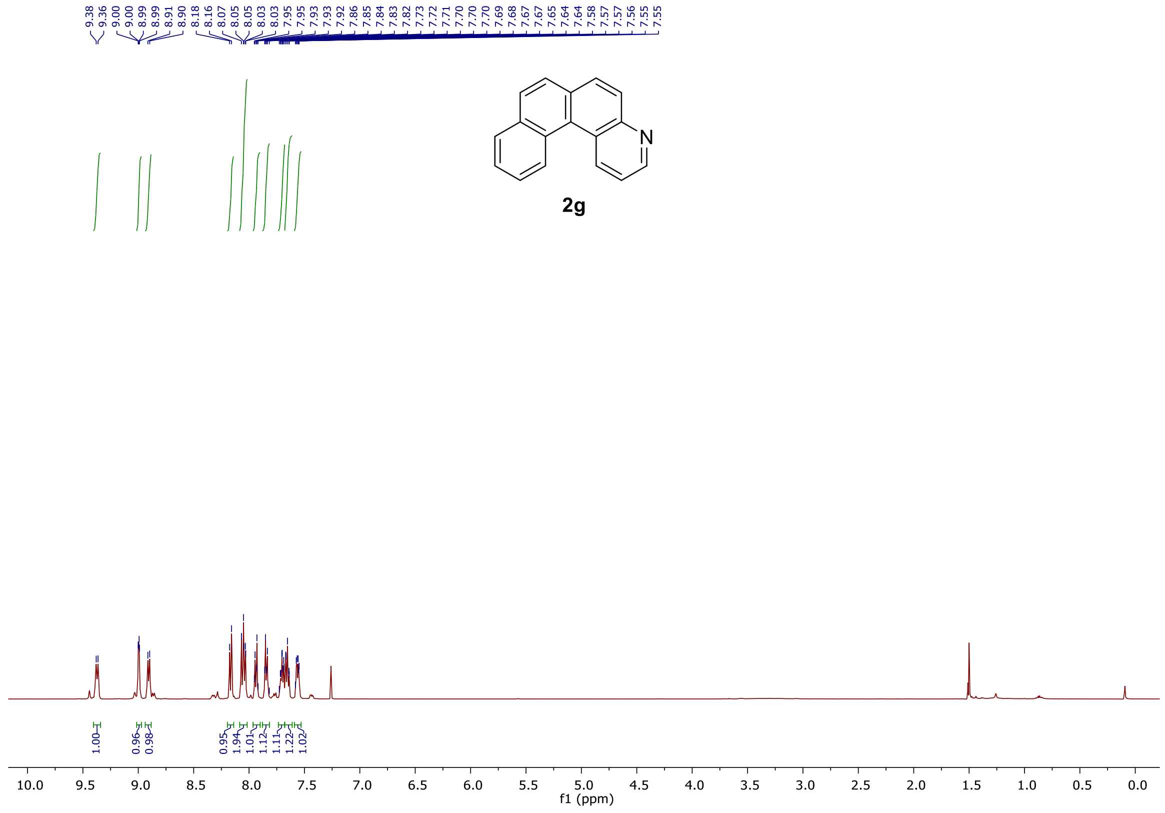

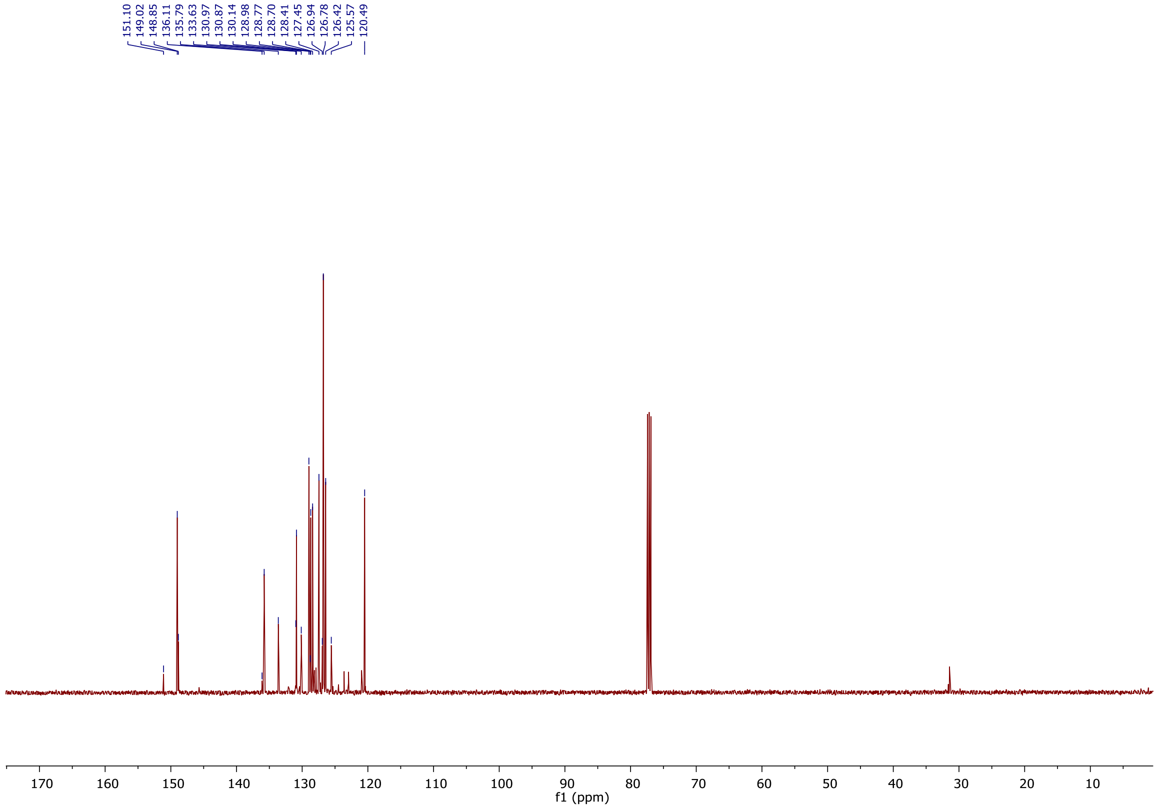

^{1}H and ^{13}C NMR traces of 2h:

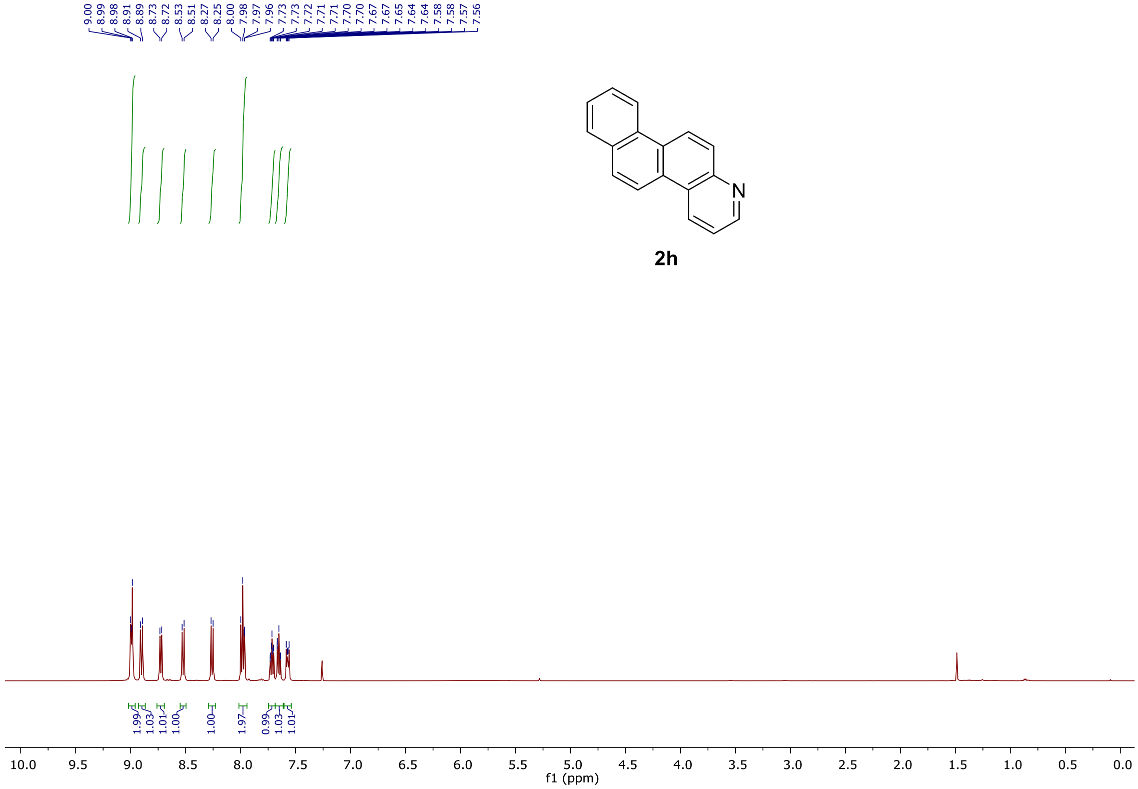

2h

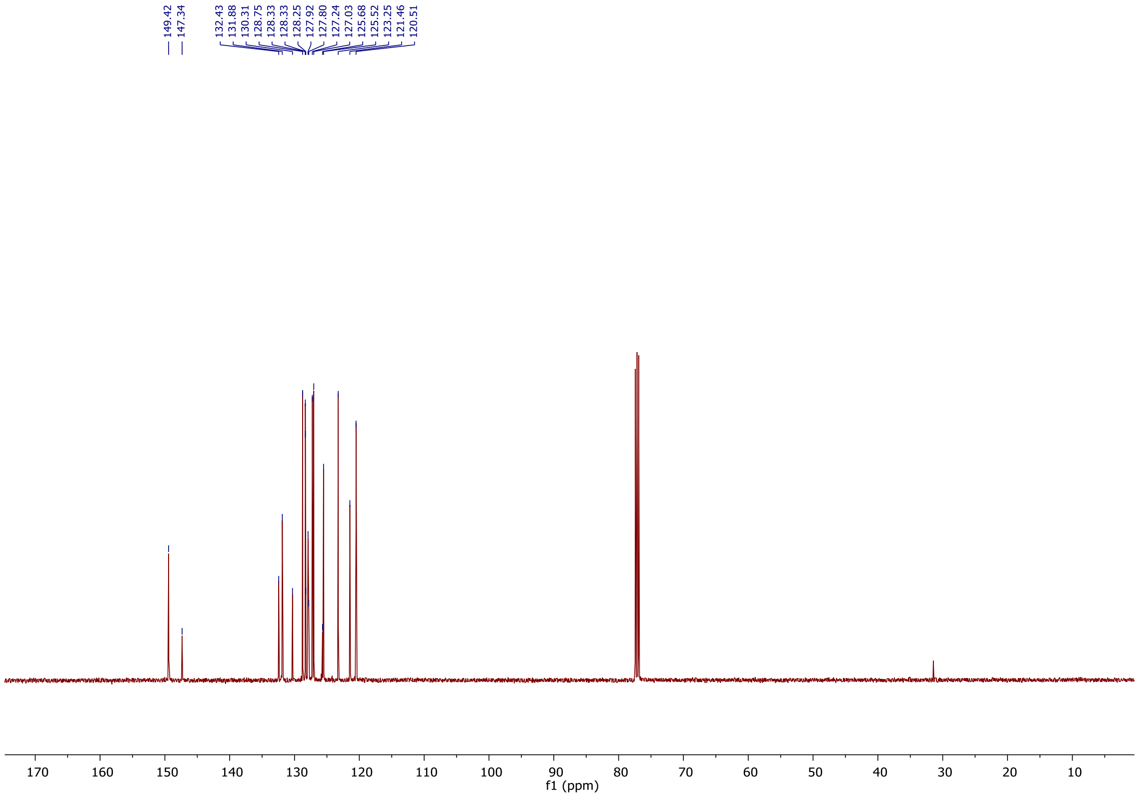

^{1}H and ^{13}C NMR traces of 2i:

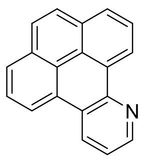

2i

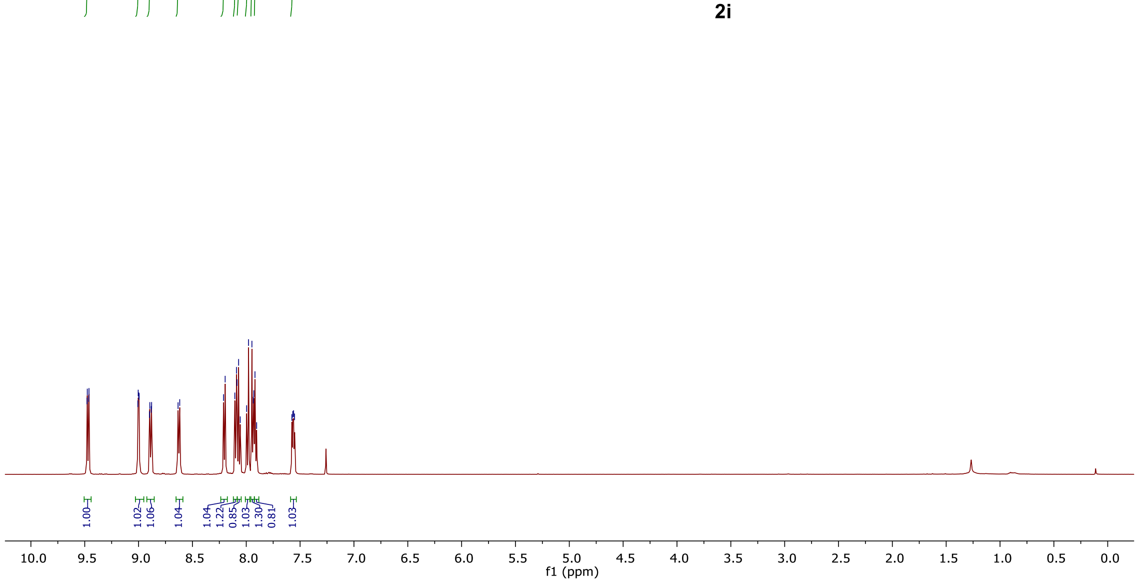

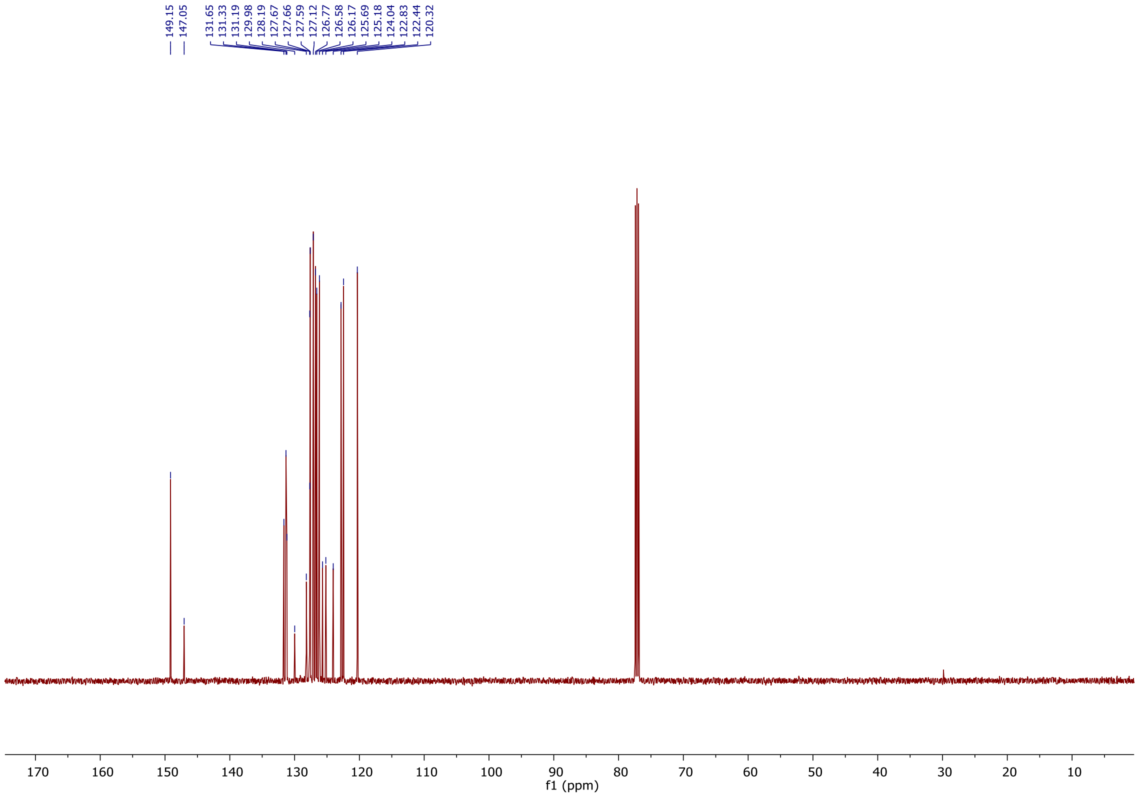

^{1}H and ^{13}C NMR traces of 2j:

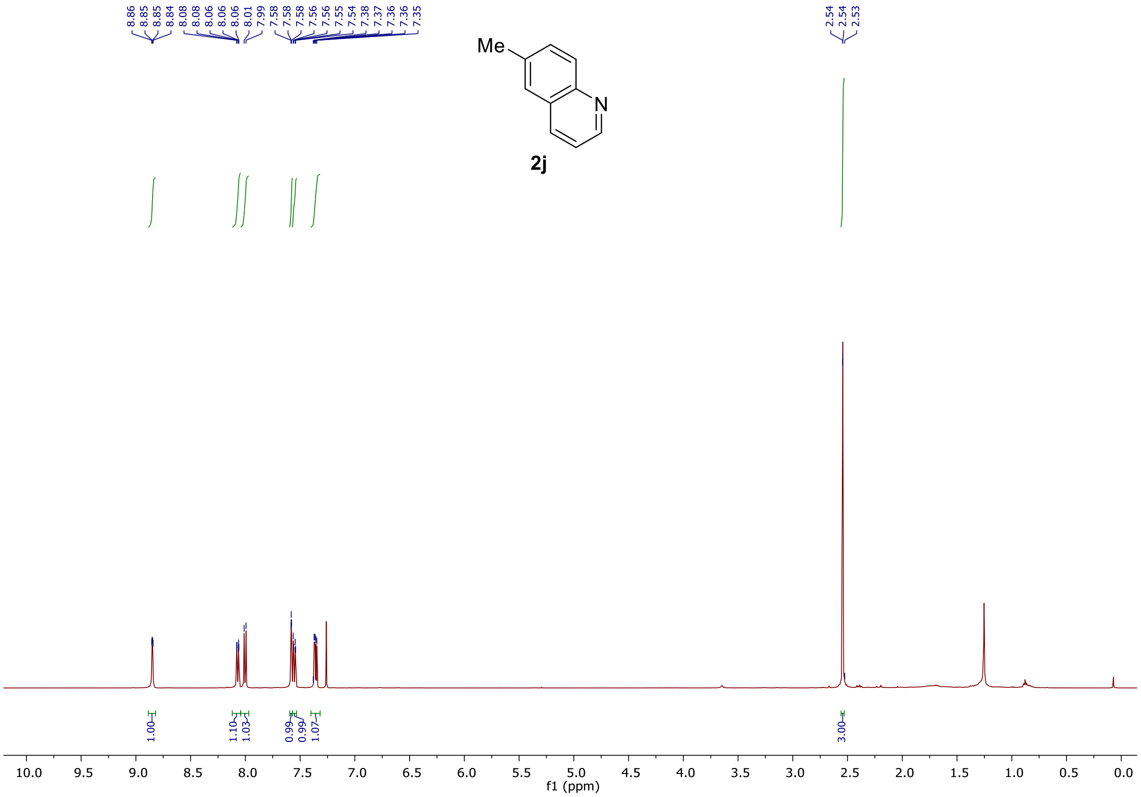

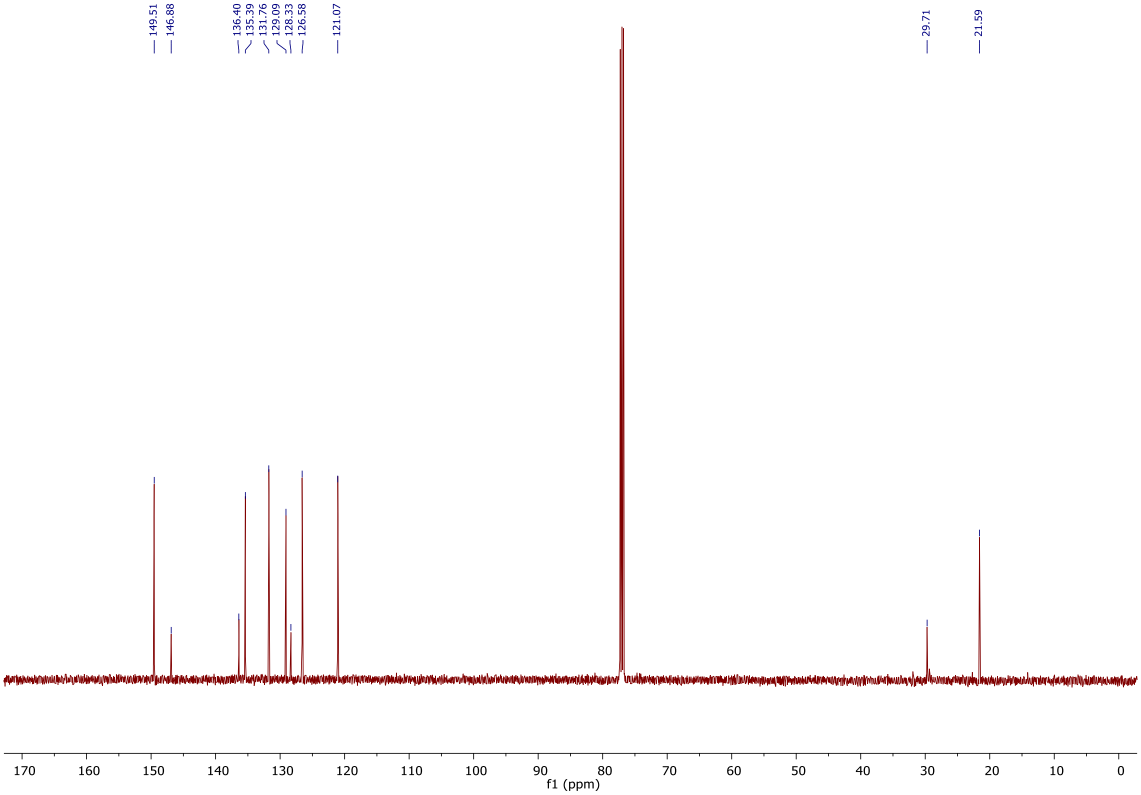

^{1}H and ^{13}C NMR traces of 2k:

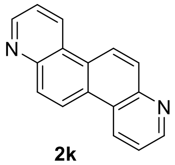

2k

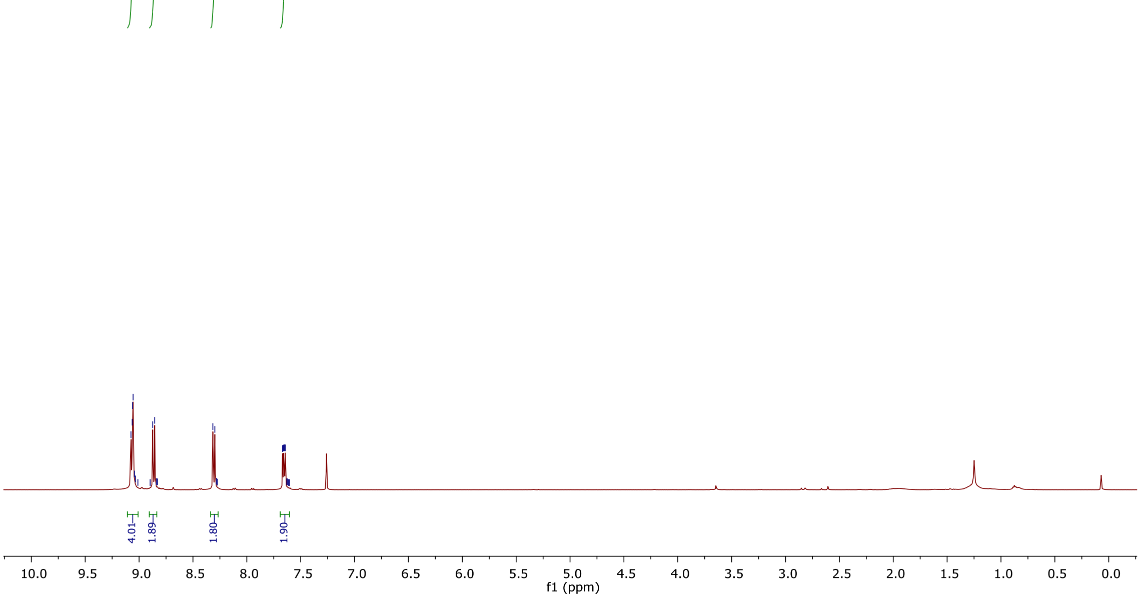

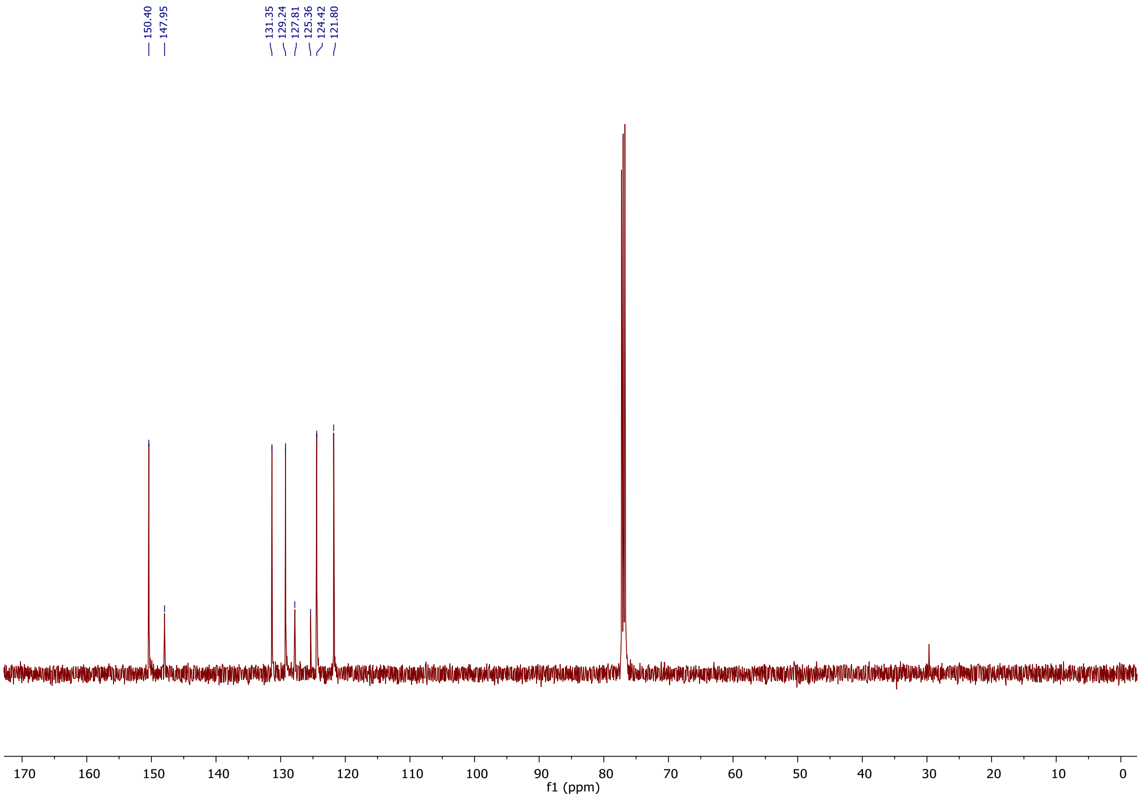

^{1}H and ^{13}C NMR traces of 2l:

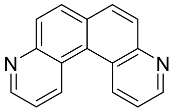

2l

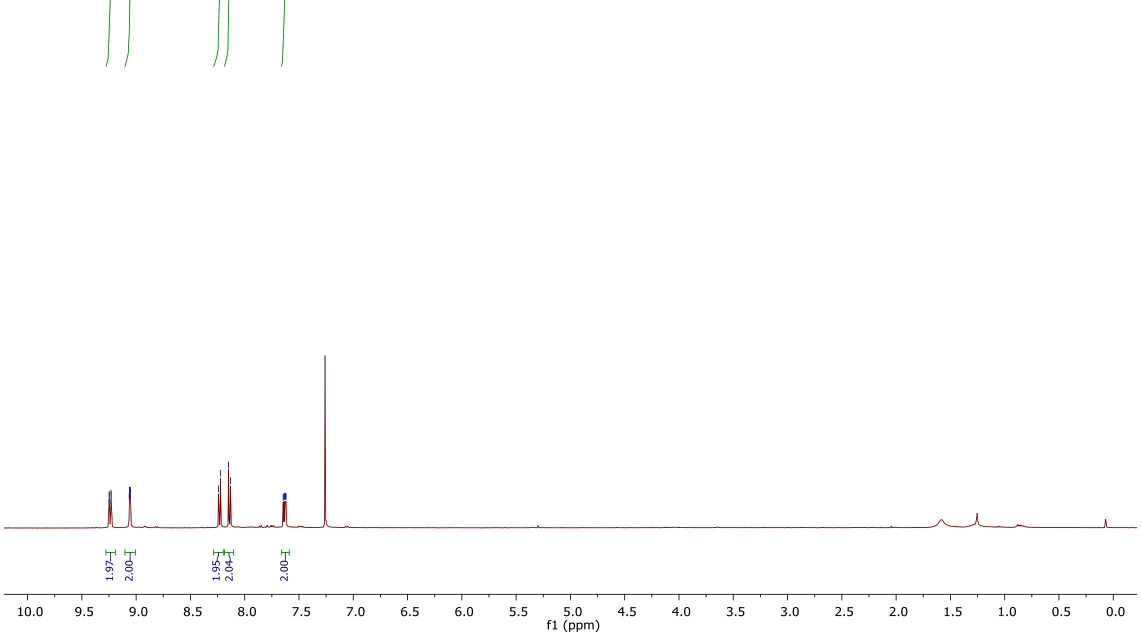

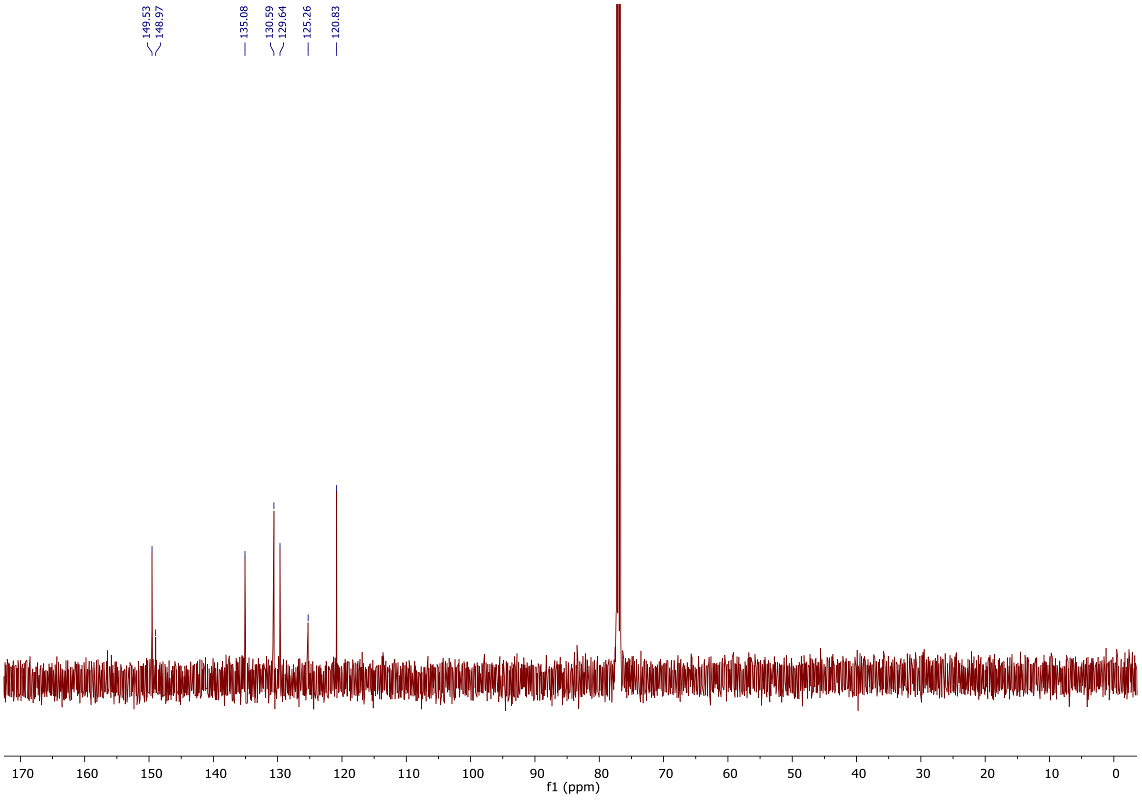

^{1}H and ^{13}C NMR traces of 4

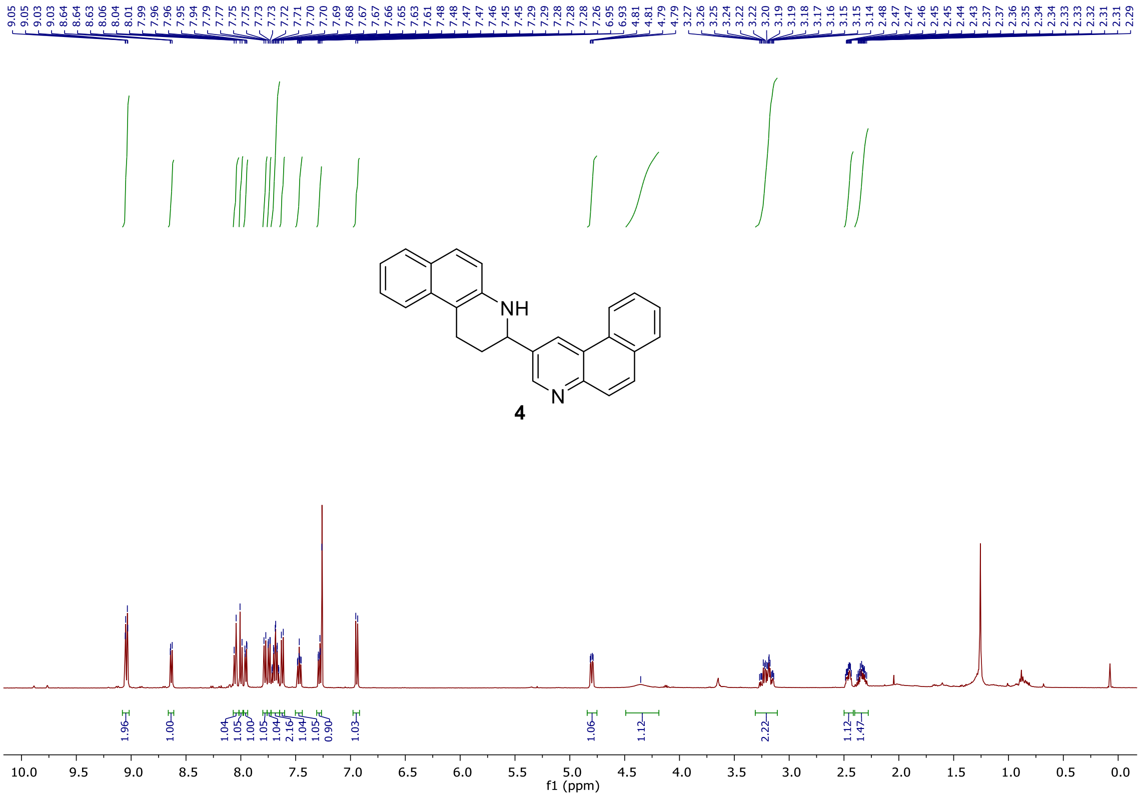

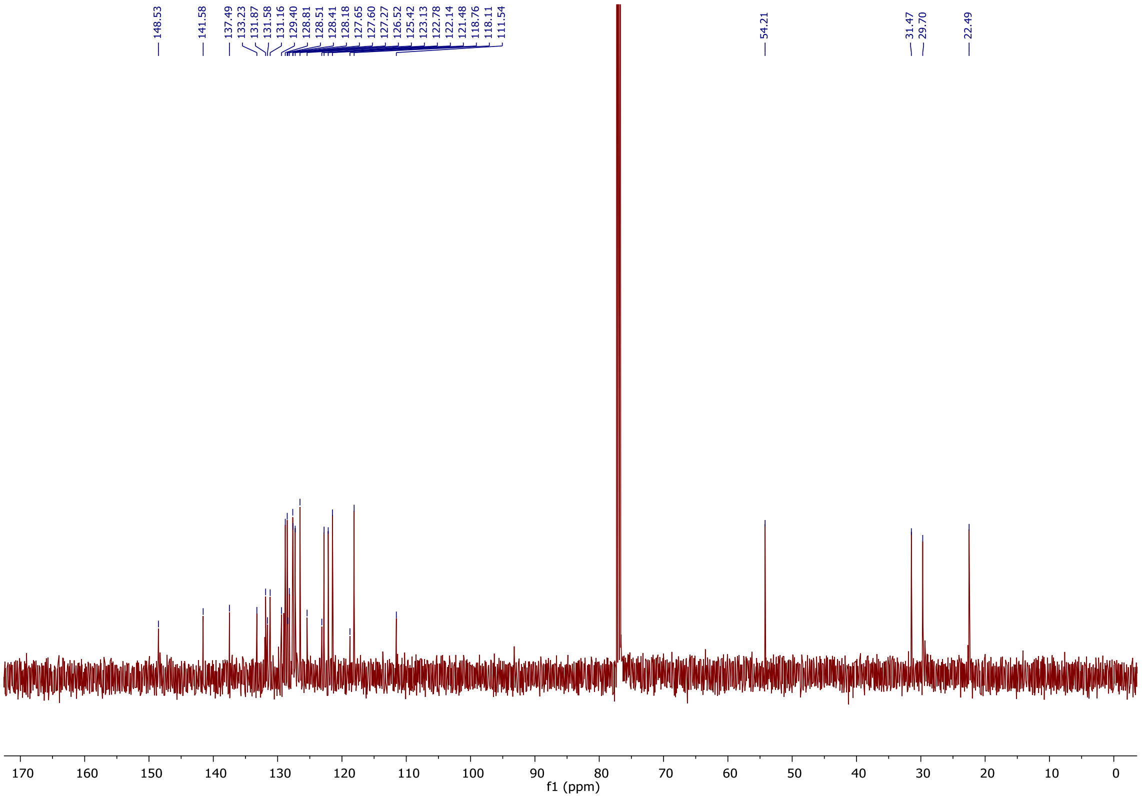

7. Computational Studies

7.1. Method

All calculations presented in this paper were carried out with a development version of the ORCA suite of programs base on version 4.2.[3] Molecular geometries were optimized in the gas-phase using the PBE functional [4] in conjunction with the D3 version of Grimme's dispersion correction with Becke-Johnson (BJ) damping function,[5] using the resolution of identity approximation. The def2- SVP basis set was used for all atoms with matching auxiliary basis. Analytic frequency calculations were performed to verify the nature of all stationary points (minima and transition states) and to calculate free energies and enthalpies at 453 K by using the rigid-rotor harmonic oscillator (RRHO) approximation, as implemented in ORCA. Transition state structures were verified by the presence of a single imaginary vibrational frequency. Single-point energies considering solvation effects (toluene) were also carried] at B3LYP-D3/def2-TZVP and M06-2X/def2-TZVP level respectively.[6] Topological analysis of the electron density distribution is performed using the AIM program [7] taking the wave function generated at the PBE-D3/def2-SVP level of theory. Strength of these hydrogen bonding interactions were also quantified using AIM following the Espionosa protocol[8] that based on the difference in the potential energy densities (V_i) at the two bond critical points following the equation: $\Delta E_{int} = [0.5*(V_{major} - V_{minor})]$ a.u

7.2. Discussion

In order to understand the role of catalyst, reaction energetics, and the nature of the key transition states (TS), we have employed DFT calculations. The activation barrier associated with the crucial uncatalyzed C–C bond forming step in pseudo-sigmatropic rearrangement (TS_4 in Fig 1) is 84.56 kcal/mol at B3LYP-D3/def2-TZVP//PBE-D3/def2-SVP level of theory. Notably, such a high activation barrier is consistent with the breaking of a sigma bond and the dearomatization during the TS. Furthermore, the overall transformation is highly exothermic (–56.85 kcal/mol) in nature, highlighting the importance of the resulting aromaticity in driving the overall transformation. While computing the key transition states (TS) in the presence of the catalyst (H_3PO_4), we considered several possibilities and modes of activation. Presence of a single molecule of H_3PO_4 within the TS framework can lead to two distinct arrangements based on the site of the protonation (TS_2 and TS_3 in Fig 1). Similarly, one can also envision a highly organized di-protonated transition state *via* the involvement of two catalyst molecules (TS_1 in Fig 1). Our calculation has revealed that diprotonated TS_1 is significantly more stable (>5 kcal/mol) than its mono-protonated counterparts. Such an observation is intriguing given the entropic penalty associated with the TS_1 (*similar trend is also observed for M06-2X/def2-TZVP//PBE-D3/def2-SVP level of theory: see Table S1*). A closer perusal of the competing TS structures has identified the several additional non-covalent interactions [9] in the TS_1 compared to TS_2 and TS_3, underscoring the importance of enthalpy in overriding the entropic bias. Moreover, such additional non-covalent stabilization also reduces the activation energy by 20 kcal/mol compared to the uncatalyzed reaction (TS_4), which is in excellent agreement to the experimental observations. Furthermore, our effort to quantify CH..O interaction using AIM indicates the difference is chiefly electrostatic in nature (see Table S2). [10] Additional credence to our computational findings comes from the observed non-linear effect in a similar asymmetric Benzidine rearrangement that had previously suggested the involvement of multiple counterions within the TS assembly.[11] Therefore, contrary to the conventional mono-protonated mechanism [12] our computational analysis has identified a favorable diprotonated pathway and offers a basis for the non-linear effect observed in an analogous asymmetric Benzidine rearrangement.

- Notably, we also wondered the possibility of having a diradical pathway as suggested in Ref 11. However, despite several trials, our efforts to identify a diradical pathway remains futile.

Table S1: Computed Thermochemical Values.

Configuration	RRHO Free Energy Correction	Energy ΔΔ E	Total Free Energy ΔΔ G(TS)	Relative Activation Energy (kcal/mol)
**B3LYP-D3/def2-TZVP // PBE-D3/def2-SVP**				
**H_3PO_4**			-644.176335	0
**Starting Material (Compound A)**			-613.108579	
**NH_3**			-56.54961300	
**H_2**			-1.17278807	
**Product F**			-555.4768221	
**Stability difference between Compound A & F**			613.1991	56.85 (exothermic)
**(A1) Un-Catalyzed Pathway**	0.09978755	-613.07351516	612.97375	84.56
**(I) protonation at Cyclopropyl side**	0.12688873	-1257.293862	-1257.1669	73.95
**(II) protonation at Napthyl side**	-0.13109961	-1257.300251	-1257.1695	72.35
**(A3) Di-protonation**	0.15766125	-1901.51503	-1901.36615	65.01
**M06-2X/def2-TZVP // PBE-D3/def2-SVP**				
**H_3PO_4**			-644.2096228	
**Starting Material (Compound A)**			-613.173783	
**NH_3**			-56.548443954	
**H_2**			-1.16795468	
**Product F**			-555.53373349	
**Stability difference between Compound A & F**			613.25	47.8 Kcal (exothermic)
**Un-Catalyzed Pathway**	0.09978755	-613.112518382	613.0127	101 Kcal
**(I) protonation at Cyclopropyl side**	0.12688873	-1257.3596803	-1257.2328	90.61
**(II) protonation at Napthyl side**	-0.13109961	-1257.3714472	-1257.2404	89.67
**(B3)Di-protonation**	0.15766125	-1901.61987531	-1901.4622	82.01

Table S2: AIM quantification of the CH..O Interactions.

Total values of the potential energy densities (V_i, in a.u.) at the important bond critical points in **TS_{major}** and **TS_{minor}**, along with the corresponding energy difference (kcal/mol)[ΔE_{int}= [0.5*(V_{major}- V_{minor})] a.u.]. This indicates that di protonated pathway enjoys 7.9 kcal more electrostatic stabilization than mono protonated pathway.

Structure	V_{Total} [x 10^{-2} (a.u)]	Δ E (int)
**TS_{major} (di protonated pathway)**	-0.1927	7.93 kcal/mol
**TS_{minor} (mono protonated pathway)**	-0.1674	

Fig 1: Key Optimized TS Structures at PBE-D3/def2-SVP level of theory.

TS for Di-protonated Pathway

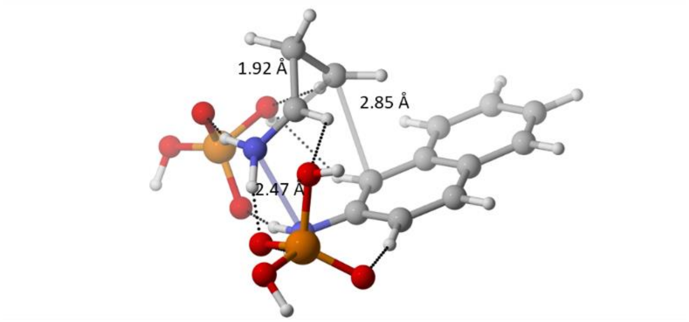

TS_1

$\Delta\Delta$ G = 65.01 kcal/mol (most stable pathway)

Multiple additional CH…O interaction
is evident from the optimized structure

TS for Mono-Protonated (cyclopropyl side) Pathway

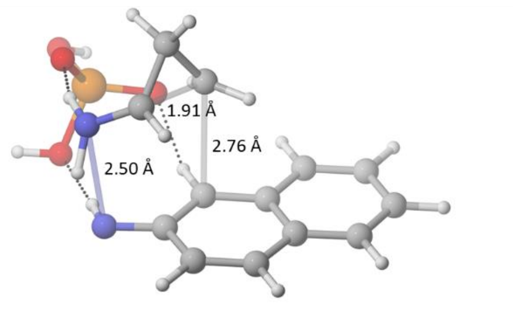

TS_2

$\Delta\Delta$ G = 73.95 kcal/mol

TS for Mono-Protonated (naphthyl side) Pathway

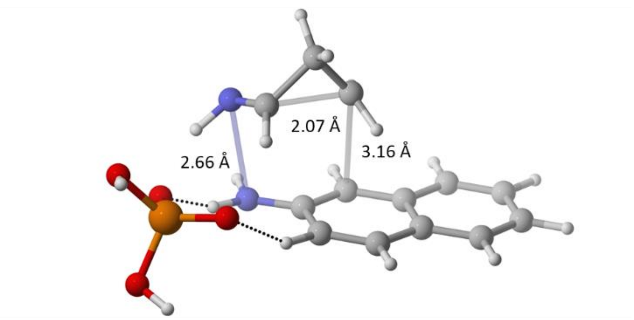

TS_3

$\Delta\Delta$ G = 72.35 kcal/mol

TS for Uncatalyzed Pathway

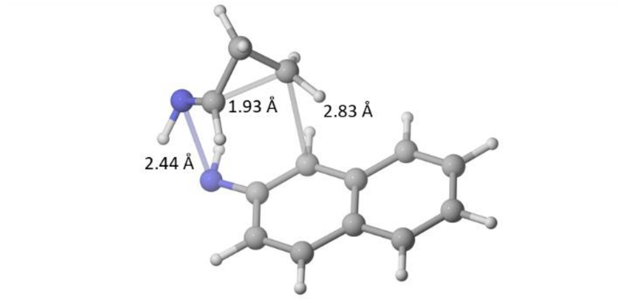

TS_4

$\Delta\Delta$ G = 84.56 kcal/mol

Optimized Cartesian Coordinates PBE-D3/def2-SVP

Optimized H_3PO_4

P	-8.50082744258246	5.67540716103932	3.95729194163040
O	-9.53020122200645	4.47782904204242	4.35180860344468
H	-10.31380724759476	4.52463904640849	3.76989389609325
O	-7.55598134783262	5.73395374710559	5.28157852538270
H	-7.91341329661948	6.40123248029626	5.89954953783979
O	-9.10935887545394	6.96451281660341	3.50774662384922
O	-7.52160902819129	4.91979949558475	2.89887415581273
H	-7.03248613971892	5.59010164091972	2.38273099594718

Optimized NH_3

N	-6.88747412152934	7.61515433752054	2.87859597345947
H	-5.98770167441179	7.42678252808043	3.34499820777943
H	-7.06001969134837	6.76800484136713	2.31720403015222
H	-7.58042568271048	7.55614558303189	3.63947076860888

Optimized H_2

H	-2.36640915007126	1.77125217000000	-2.92142655000000
H	-3.13461792992874	1.77125217000000	-2.92142655000000

Optimized Starting Material (Compound A)

C	-11.62597676000000	6.42622985000000	-2.25101635000000
C	-10.27227053000000	6.18766603000000	-2.52946370000000
C	-9.30692664000000	6.40192761000000	-1.53659752000000
C	-9.69346356000000	6.85080796000000	-0.27689362000000
C	-11.04288229000000	7.09146305000000	0.00949883000000
C	-12.01170420000000	6.87847895000000	-0.98058880000000
H	-7.65516801000000	5.82171768000000	-2.77250000000000
H	-12.36459025000000	6.26321210000000	-3.00783557000000
H	-9.97712451000000	5.84265280000000	-3.49835724000000
C	-7.94784147000000	6.16670556000000	-1.80284769000000
C	-8.60935718000000	7.07199362000000	0.77788933000000
H	-11.33305160000000	7.43641984000000	0.97991451000000

H	-13.04434032000000	7.06065630000000	-0.76757696000000
C	-7.32181686000000	6.82358984000000	0.44642220000000
C	-6.99228621000000	6.37001294000000	-0.84725726000000
H	-8.86392820000000	7.41631793000000	1.75846792000000
H	-5.96699113000000	6.17996587000000	-1.08715747000000
N	-6.25915607000000	7.02782868000000	1.44137876000000
H	-5.50948026000000	6.39022114000000	1.26405759000000
N	-5.78453300000000	8.34203057000000	1.35416451000000
H	-5.41873920000000	8.50420068000000	0.43770624000000
C	-6.88655645000000	9.27931366000000	1.61482663000000
C	-6.71750935000000	10.39501694000000	2.67588952000000
C	-6.65590672000000	10.75031876000000	1.18801237000000
H	-7.83254534000000	8.82830012000000	1.39897527000000
H	-5.83147538000000	10.39674654000000	3.27575683000000
H	-7.56491708000000	10.66492941000000	3.27082430000000
H	-7.45027888000000	11.33073729000000	0.76731390000000
H	-5.71705199000000	11.06141482000000	0.77975873000000

Optimized Pdt F

C	-12.31239696618401	-9.76230093249078	9.39513782009737
C	-10.33884906186404	-8.56577446295262	9.39558463608134
C	-11.02711471995809	-7.30365047329603	9.39852021448542
C	-12.44424647367102	-7.35300431937194	9.39968579521940
C	-13.09446843586648	-8.58195205982706	9.39800600041160
C	-8.90037844393694	-8.58814305428011	9.39425755853445
C	-10.26007936801464	-6.06510398431174	9.40012092686545
C	-8.82516319162245	-6.13782623340372	9.39869995070194
C	-8.17753780837037	-7.42252250765425	9.39575664147046
C	-8.06607554265072	-4.93643730556932	9.40022822887992
H	-6.96723097449708	-5.00876622915131	9.39910883490107
C	-8.68972985006913	-3.69400034354046	9.40307427274791
C	-10.10192868631123	-3.61894754427438	9.40448743841778

C	-10.86743341059121	-4.78173299888754	9.40303616874362
H	-8.41953866849258	-9.57675851862295	9.39201237987342
H	-12.80880432996128	-10.75032376118836	9.39376807208133
H	-13.03311952454927	-6.42437910205820	9.40190250403917
H	-14.19284626338636	-8.64299852050489	9.39887082439493
H	-7.07651495073487	-7.44842934598531	9.39472768166320
H	-8.08949746562557	-2.77174058092867	9.40422751050828
H	-10.59905091082617	-2.63718540270969	9.40674651889425
H	-11.96334145547461	-4.69636854564499	9.40418997386536
N	-10.98466506734182	-9.76478004334566	9.39395301712224

Key Optimized TS structures

(1) Un-Catalyzed TS

Imaginary frequency = - 377.91

C	-11.42936725897651	7.65111600421698	-2.60127209447025
C	-10.17710058504691	7.05066703636358	-2.72823371433128
C	-9.53279514815579	6.46250283377635	-1.61192993067496
C	-10.19518011948071	6.48183603634164	-0.32963177208548
C	-11.47254702652757	7.10745448372084	-0.22861230600083
C	-12.07735384233836	7.68169060588611	-1.34253646322004
H	-7.73235605171028	5.83740602832859	-2.68408027000873
H	-11.91530157263940	8.10307437940874	-3.47900210164290
H	-9.66924730934439	7.02801680122116	-3.70544041271669
C	-8.23382970792130	5.84998323757981	-1.70311291127132
C	-9.53474768559581	5.92195481696906	0.79776458257872
H	-11.97891186704638	7.12225187574476	0.74944254734432
H	-13.06499927473502	8.15793928876415	-1.24731034787061
C	-8.26726758962817	5.27463371166666	0.69784975421544
C	-7.62624356757315	5.28660202702636	-0.60848497291609
H	-10.05809394768488	5.89796098338228	1.76637556452022
H	-6.63777812271697	4.80843552733633	-0.68035220996131
N	-7.60137343982608	4.73536910970591	1.72704428321359

N	-6.72299119738089	6.77571467926405	2.75365099156316
H	-6.23563229188566	5.95586665684906	2.33905655908318
C	-6.93500778921955	7.69735104467314	1.84659235593661
C	-7.48139654996495	9.06621926197376	2.25941119342531
C	-8.72169668202630	8.45046795021788	1.79673197465849
H	-6.64954648692647	7.61333561844688	0.77704940678619
H	-7.07213298346464	9.88894944026002	1.64727958153733
H	-7.38319132489902	9.23216039275039	3.34492650415869
H	-9.39339505005062	7.97822547345520	2.52397266455507
H	-9.08994598095304	8.59044863298465	0.77232461778266
H	-8.19185208628002	4.77520578168490	2.57466185581150

2. Mono-Protonation TS

(A) Cyclopropyl protonation TS

Imaginary incidence = - 289.12

C	-11.34290422483621	7.43140819568620	-2.41472626631580
C	-10.02832828385391	6.97002116432949	-2.51964672808095
C	-9.36055970399594	6.43154727481237	-1.39483525527916
C	-10.06732601839447	6.35345240005109	-0.14111944893151
C	-11.40234652976763	6.84402947654684	-0.05476050753125
C	-12.02981471142717	7.37503841429760	-1.17829173265072
H	-7.45760517887975	6.04624586092267	-2.40690767856665
H	-11.84726501338057	7.84791663984286	-3.29984445261116
H	-9.49668464474227	7.02552102693683	-3.48250119264743
C	-7.99311330645146	5.97419544160476	-1.44684595643833
C	-9.40632233448581	5.83445663546361	1.00297574793453
H	-11.91357482739287	6.79236332954814	0.91909752912524
H	-13.06137069300869	7.75126106587649	-1.10823887257418
C	-8.07153235118337	5.31671609551615	0.92467066386141
C	-7.36689925101517	5.45424014961575	-0.34265959131593
H	-9.95612125029176	5.77191302698947	1.96099571861628
H	-6.33020507213328	5.08791954404044	-0.38585223476185
N	-7.43766568990835	4.78488489624567	1.97274534417708

N	-6.74404299630669	6.92963261262524	3.05437090211015
H	-6.45705296727841	5.99313853545376	2.64287461595710
C	-7.07265381912215	7.89873136545523	2.20346116909057
C	-7.83761183496636	9.11508834918714	2.64671837399265
C	-8.89823694551484	8.39287811866545	1.92224455694727
H	-6.62058799597188	7.89998173685079	1.19868601878148
H	-7.46336356436787	10.05357689166255	2.19865531333615
H	-7.97389275625699	9.14858519206140	3.74225493829424
H	-9.65651293024859	7.84078006436381	2.51120839085592
H	-9.07352538199126	8.59128731441387	0.85544847422345
H	-8.10279399018007	4.67678006749984	2.76802707308697
P	-9.66773561304326	6.23343947753188	4.82102914727183
O	-9.19088529535860	4.64524650735281	4.52912373122024
H	-8.54258686965551	4.38928434196847	5.21485942238138
O	-8.38192142135456	7.04163384223736	5.06520390107081
H	-7.32487143141993	6.91426505233590	3.98703118924325
O	-10.65213979577820	6.56801749689305	3.70954407609032
O	-10.43526928334786	6.14332471142255	6.27424468886890
H	-11.38078709268602	5.98434841369103	6.08725863116744

(B) Napthyl Protonation TS

Imaginary frequency = - 120.27

C	-11.78939228303659	7.66984426168700	-2.53744324511718
C	-10.43239086756352	7.38053891486784	-2.60278962407343
C	-9.76816005903199	6.74190327180194	-1.51930208040632
C	-10.52865366190193	6.38531909083007	-0.33962930129819
C	-11.92244208832041	6.70599781127907	-0.30248251199655
C	-12.53677762606890	7.33191125702208	-1.37581311002856
H	-7.77765957052616	6.74842145523918	-2.41650364630082
H	-12.28997743929940	8.16319217554773	-3.38399418858025
H	-9.84816515807742	7.64331450184508	-3.49859879891716
C	-8.36841627608863	6.45098383123333	-1.53571085940792

C	-9.87324437626632	5.74465528931561	0.73694390663864
H	-12.50290499274842	6.43804069830747	0.59422322166758
H	-13.61090226117246	7.56756639465412	-1.33247075001916
C	-8.49340974212812	5.43211097380736	0.68840110357309
C	-7.73337601141334	5.84081509021791	-0.47234530878760
H	-10.45798933477772	5.46158400978758	1.62731729085478
H	-6.63823002866928	5.69617911736788	-0.48072064438605
N	-7.87664837180949	4.79516421872104	1.70576340047143
H	-6.81991711523419	4.64216924895914	1.75890689110809
N	-6.88490979199910	6.91444713385644	2.97981812433755
H	-6.18666972521259	6.11492130757189	2.90652293834032
C	-6.69341406639241	7.69887220213227	1.98246510894069
C	-7.35587335229607	9.13284229428234	1.97482647876736
C	-8.46953455910258	8.52102362687411	1.30311324257416
H	-6.04250040701238	7.47849309637014	1.09879533208603
H	-6.72159140906010	9.80852225280715	1.37501480416963
H	-7.52721828800770	9.47512498891618	3.00884615591372
H	-9.31622560811411	8.11933385301226	1.87637358149307
H	-8.48758387475191	8.38505705700019	0.21193663959982
H	-8.42218864940006	4.56472407115421	2.53697841743904
O	-5.27563508100579	4.76607082651920	2.18579380919391
O	-4.66791482426705	6.25328493949722	0.06952699056417
O	-3.01774502353871	5.86565527229412	2.08656153848217
H	-2.34444050438040	6.25454730927771	1.49650307345189
P	-4.23868528821705	5.26511692367275	1.15932686010985
O	-3.54080573402313	3.95803650821382	0.42319480637904
H	-3.63253726908305	4.11511267405528	-0.53583899683688

(3) Di protonated TS

Imaginary frequency = - 350.13

C	-11.28598732134313	7.99393729944468	-2.32727115243547
C	-10.07335622575476	7.34350545992558	-2.55518317486783

C	-9.38848168561108	6.69526267344855	-1.49792082302707
C	-9.97910773600849	6.70312740884106	-0.18002240631972
C	-11.21289279293713	7.38942035048541	0.02769878006981
C	-11.85459035671105	8.02414940121102	-1.02894559896077
H	-7.67596539049807	6.03281478690458	-2.68691543963981
H	-11.80390560792989	8.49335520106900	-3.15992906133209
H	-9.63097121240513	7.33009374304262	-3.56336828891951
C	-8.12235659624384	6.03947673641551	-1.67996499020863
C	-9.29757308642017	6.06938581370666	0.88389136020758
H	-11.64104236014918	7.40604985357302	1.04164197338494
H	-12.80599382232648	8.55043131771371	-0.86110967559481
C	-8.05189374438921	5.41447323205273	0.67431505763276
C	-7.45506517606498	5.42695612025378	-0.64456015065723
H	-9.73746836265658	6.03922635728807	1.89154925952008
H	-6.48149255014209	4.92560423174004	-0.81924498635511
N	-7.43381166023635	4.84898689186142	1.72333317174724
H	-7.95002862342559	4.83675435439305	2.66428387017507
N	-6.08701577786383	6.75017838433835	2.54657185768899
H	-5.41347400842432	5.93583662680753	2.24538420601266
C	-6.23784626743607	7.75186764643320	1.69816487593532
C	-6.78438714199785	9.10307602954561	2.11690111798247
C	-7.98358879259875	8.52845130911774	1.50906788034630
H	-5.76614351242663	7.65220672271666	0.70585591337843
H	-6.28401203567419	9.92398844622272	1.57265772972273
H	-6.83746416799300	9.22240882508717	3.21271277913020
H	-8.77759361466024	8.15363317357392	2.17103302220883
H	-8.19430512539398	8.63461228919309	0.43515649896852
H	-6.45806795417805	4.46697929114700	1.60658279107055
O	-4.75810219880937	4.73005298427111	1.62965487945264
O	-4.55226277837189	4.31355120731807	-0.99082526050819
O	-3.73260853721384	6.51908562773304	0.20514734916186
H	-3.30456121434640	6.71528683405152	-0.65040954635920
P	-3.97898652684163	4.86977836914988	0.29690082189756
O	-2.44683454317394	4.30759292743652	0.52525488804287

H	-2.31723488876225	3.60535745004155	-0.14095010597042
P	-8.85945006649941	6.47742098305830	4.62702361893705
O	-9.42432038926326	6.29867975438980	6.16309177845119
H	-9.31283845658341	5.35640855486921	6.39607756842103
O	-7.58423060892418	7.32289361073541	4.57168047678772
H	-6.58142857608990	6.86540863559018	3.51428365567425
O	-8.87844649101174	5.10213475768632	3.94538275960751
O	-10.04302865865626	7.46647040828537	3.99585924813221
H	-10.91775894554677	7.08724265782522	4.20862114140673

8. References

[1] K. Juhl, M. Jessing, M. Langgard, P. J. Vieira Vital, M. Marigo, J. Kehler, L. K. Rasmussen (H. Lundbeck A/S), US-20170291901A1, **2017**.

[2] V. Mamane, F. Louerat, J. Iehl, M. Abboud, Y. Fort, *Tetrahedron* **2008**, *64*, 10699–10705.

[3] F. Neese, Wiley Interdiscip. Rev.: Comput. Mol. Sci. 2011, 2, 73–78.

[4] Y. Zhang, W. Yang, Phys. Rev. Lett. 1998, 80, 890–890.

[5] a) S. Grimme, J. Antony, S. Ehrlich, H. Krieg, J. Chem. Phys. 2010, 132, 154104; b) S. Grimme, S. Ehrlich, L. Goerigk, J. Comput. Chem. 2011, 32, 1456–1465.

[6] Y. Zhao, D. G. Truhlar, *Theor. Chem. Acc*. **2008**, 120, 215–241.

[7] a) R. F. W. Bader, *Accounts Chem. Res.* **1985**, *18*, 9-15;b) R. F. W. Bader, *Chem. Rev.* **1991**, *91*, 893–928;(c) E. Espinosa, E. Molins, C. Lecomte, *Chem. Phys. Lett.* **1998**, *285*, 170–173.

[8] E. Espinosa, E. Molins, C. Lecomte, *Chem. Phys. Lett.* **1998**, *285*, 170–173.

[9] a) R. Maji, P. A. Champagne, K. N. Houk, S. E. Wheeler, *ACS Catalysis* **2017**, *7*, 7332–7339 b) R. Maji, C. S. Mallojjala, S. E. Wheeler, *Chem. Soc Rev.* **2018**, 4*7*, 1142–1158.

[10] a) R. C.Johnston, P. H.-Y. Cheong, *Org. Biomol. Chem.***2013**, *11*, 5057–5064; b) R. Maji, S. E. Wheeler, *J. Am. Chem. Soc.* **2017**, 139, 12441–12449 c) R. Maji, H. Ugale, S. E. Wheeler, *Chem Eu Jr.* **2019**, 25**,** 4452–4459.

[11] C. K. De, F. Pesciaioli, B. List, *Angew. Chem. Int. Ed*. **2013**, *52*, 9293–9295.

[12] T. J. Seguin, T. Lu, and S. E. Wheeler, *Org. Lett*. **2015**, *17*, 3066–3069; b) P. Maity, R. P. Pemberton, D. J. Tantillo, U. K. Tambar, *J. Am. Chem. Soc.* **2013**, 135, 16380–16383, c) N. Çelebi-Ölçüm, B. W. Boal, A. D. Huters, N. K. Garg, K. N. Houk*, J. Am. Chem. Soc*. **2011**, 133, 5752–5755.
